# Stevens–Johnson Syndrome and Toxic Epidermal Necrolysis: A Systematic Review of Ophthalmic Management and Treatment

**DOI:** 10.3390/vision9030078

**Published:** 2025-09-11

**Authors:** Korolos Sawires, Brendan K. Tao, Harrish Nithianandan, Larena Menant-Tay, Michael O’Connor, Peng Yan, Parnian Arjmand

**Affiliations:** 1 Faculty of Medicine, Dalhousie University, Halifax, NS B3H 4R2, Canada; ksawires@dal.ca; 2 Department of Ophthalmology and Vision Sciences, University of Toronto, Toronto, ON M5T 3A9, Canada; brendan.tao@mail.utoronto.ca (B.K.T.);; 3 Retina Division, Cleveland Clinic, Cole Eye Institute, Cleveland, OH 44195, USA; 4 Bathurst Dundas Dental Centre, Toronto, ON M6J 1T8, Canada; 5 Division of Pediatrics and Strabismus, Department of Ophthalmology, University of Ottawa, Ottawa, ON K1H 1C4, Canada; 6 Mississauga Retina Institute, Mississauga, ON L4X 2Z9, Canada; 7 School of Medicine, Toronto Metropolitan University, Toronto, ON M5B 2K3, Canada

**Keywords:** Stevens–Johnson Syndrome, Toxic Epidermal Necrolysis, ophthalmology, corneal disease, ocular complications, multimodal treatment, contact lenses

## Abstract

**Background:** Stevens–Johnson Syndrome (SJS) and Toxic Epidermal Necrolysis (TEN) are rare, life-threatening mucocutaneous disorders often associated with severe ophthalmic complications. Ocular involvement occurs in 50–68% of cases and can result in permanent vision loss. Despite this, optimal management strategies remain unclear, and treatment practices vary widely. **Methods:** A systematic review was conducted in accordance with PRISMA guidelines and prospectively registered on PROSPERO (CRD420251022655). Medline, Embase, and CENTRAL were searched from 1998 to 2024 for English-language studies reporting treatment outcomes for ocular SJS/TEN. **Results:** A total of 194 studies encompassing 6698 treated eyes were included. Best-corrected visual acuity (BCVA) improved in 52.2% of eyes, epithelial regeneration occurred in 16.8%, and symptom relief was reported in 26.3%. Common treatments included topical therapy (n = 1424), mucosal grafts (n = 1220), contact lenses (n = 1134), amniotic membrane transplantation (AMT) (n = 889), systemic medical therapy (n = 524), and punctal occlusion (n = 456). Emerging therapies included TNF-alpha inhibitors, anti-VEGF agents, photodynamic therapy, and 5-fluorouracil. **Conclusions:** Disease-stage-specific therapy is crucial in ocular SJS/TEN. Acute interventions such as AMT may prevent long-term complications, while chronic care targets structural and tear-film abnormalities. Further prospective studies are needed to standardize care and optimize visual outcomes.

## 1. Introduction

Stevens–Johnson Syndrome (SJS) and its more severe variant Toxic Epidermal Necrolysis (TEN) are severe epidermal bullous diseases affecting the skin and mucous membranes [1,2]. While the incidence of SJS and TEN is rare, with approximately two cases per million persons per year, the mortality rates for SJS and TEN remain high: 1–5% and 25–30%, respectively [3,4,5]. A variety of drugs, such as antibiotics, non-steroidal anti-inflammatory drugs, and anticonvulsants, have been reported to induce SJS and TEN [1].

Survivors of these diseases often experience severe eye irritation and reduced vision. The reported incidence of ocular complications in SJS and TEN patients is 50–68% [1]. In the acute stage, defined as the first 2 weeks of disease, SJS or TEN patients manifest systemic prodromes of respiratory dysfunction, fever, and coryzal symptoms resembling the common cold with onset of mucocutaneous lesions [2]. Orbital and ophthalmic manifestations include vesiculobullous skin lesions and corneal or conjunctival epithelial defects due to extensive ocular surface inflammation [3]. In the chronic stage, defined as more than 6 months after the acute onset of symptoms, corneal epithelial stem cell deficiency, ectropion/entropion, trichiasis, and symblepharon and ankyloblepharon formation may complicate the course of disease [6,7]. Severe corneal opacities, persistent corneal defects, and dry eye symptoms may also persist at the chronic stage despite improvement of acute-stage impairments. Unfortunately, patients with SJS or TEN often require life-long management for ocular discomfort or chronic morbidity [8].

Although the exact pathophysiology of SJS and TEN remains unclear, an immune-mediated cytotoxic T-cell response to an external antigen is thought to play a role in the pathogenesis of the disease [9]. The proposed mechanism involves the interaction between soluble or membrane-bound Fas and Fas ligands (FasL/CD95L) on keratinocytes. Elevated FasL levels secreted by mononuclear cells in SJS and TEN patients activate the Fas signaling cascade, which results in widespread keratinocyte apoptosis and subsequent epithelial necrosis [10,11,12]. Inflammatory cytokines such as members of the tumor necrosis factor (TNF) family and interferon-γ may also contribute to further epidermal necrosis [13,14,15]. In addition, a genetic component in susceptibility to SJS or TEN is suspected, as the afflicted have an intrinsically impaired ability to detoxify reactive intermediate drug metabolites [9].

The histopathology of SJS and TEN is characterized by vacuolization of epidermal cells and necrosis of keratinocytes within the epidermis, along with dermoepidermal detachment and perivascular lymphocytic infiltration [9,16]. In SJS, extensive epidermal necrosis with sparse inflammatory cells is observed. In TEN, full-thickness necrosis of the dermoepidermal layer results in subepidermal blistering, which often resembles superficial burns. The percentage of dermoepidermal detachment is a major prognostic factor in predicting future complications, recurrence, and mortality [17].

Loss of corneal epithelial stem cells is commonly observed in SJS and TEN patients with chronic ocular complications. The event is predicated by the loss of palisades of Vogt (POV) in the limbal area [18]. Irreversible loss of corneal epithelial stem cells in the acute stage of SJS or TEN results in the conjunctivalization and neovascularization of the cornea, which, in turn, leads to severe visual loss. In the absence of irreversible complications, the epidermal layer regenerates smoothly upon remission of disease in both SJS and TEN [9].

It is generally agreed that effective management of SJS and TEN involves rapid diagnosis of the disease and identification and immediate cessation of all disease-inducing agents [19]. Currently, there are several treatment methods for SJS and TEN. These include systemic corticosteroids, intravenous immunoglobulin (IVIG), cyclosporines, cyclophosphamides, plasmapheresis, *N*-acetylcysteine, and amniotic membrane transplantation (AMT) [6,19,20]. Additionally, sweeping fornices, use of symblepharon rings, and stem cell transplantation are other means to prevent future scar formation [19]. The use of systemic corticosteroids in patients with SJS and TEN is controversial, despite the beneficial effects during the acute stage [21]. Increased mortality and morbidity by increasing patients’ susceptibility to infection and gastrointestinal bleeding were demonstrated by several studies regarding corticosteroid use [19]. IVIG usage not only decreases mortality but also results in rapid cessation of skin lesions. It is postulated that IVIG works by arresting Fas-mediated immune keratolysis [20]. Theamniotic membrane is the innermost layer of the placental membrane and consists of a thick basement membrane and an avascular stroma [22]. In both SJS and TEN, cryopreserved AMT suppresses inflammation, promotes healing, and prevents ulcer formation. The anti-inflammatory and anti-scarring effects of AMT also promote limbal epithelial stem cell expansion [22].

Although a broad range of treatments exist, no treatment modality has been established as the gold standard for patients with SJS or TEN to date. The aim of this study is to evaluate and compare ophthalmic management and treatment methods for SJS and TEN in the past 26 years.

## 2. Methods

This study was registered on PROSPERO (CRD420251022655) and adheres to the PRISMA guidelines and the tenets of the Declaration of Helsinki. We conducted a comprehensive search of Medline, Embase, and CENTRAL on 1 March 2025, covering studies from 1 January 1998 to 31 December 2024 (Supplementary Table S1). All references were managed in Covidence (Veritas Health Innovation, Melbourne, Australia). Two reviewers (K.S. and B.T.) independently completed the study screening, full-text review, data collection, and risk-of-bias (ROB) assessment using the relevant Joanna Briggs Institute (JBI) checklists. Discrepancies were resolved by a third reviewer. All randomized controlled trials (RCTs), non-randomized controlled trials (NRCTs), cohort studies, cross-sectional studies, case series, and case reports investigating the treatment of ophthalmic manifestations or complications of SJS/TEN were eligible for inclusion. Exclusion criteria included articles with a non-ophthalmic focus, absence of measurable ocular outcomes, or non-English publication.

A data extraction form was created on Microsoft Excel (v16.95.1; Redmond, Washington, United States). From each article, we collected the following data: study characteristics (i.e., year of publication and study design), patient characteristics (i.e., number of SJS/TEN patients, number of eyes treated, treatment modalities), and treatment characteristics (i.e., treatment modalities, changes in best corrected visual acuity (BCVA), changes in ocular symptoms, evidence of epithelial regeneration, and associated complications). For each included study, outcomes were extracted from the final reported follow-up visit, ensuring that the most recent ocular outcomes were captured as the best indicator of treatment efficacy. Given the heterogeneity of study designs and outcome reporting, descriptive statistics were used. Studies’ results were summarized in tables and text to illustrate the efficacy and complications associated with different treatment modalities. No pooled effect estimates or statistical comparisons were generated. For studies where eyes received multiple interventions, those eyes were included in all relevant treatment groups. Given the descriptive nature of the synthesis and absence of pooled statistical analyses, no clustering adjustment was performed.

## 3. Results

**Figure 1:**
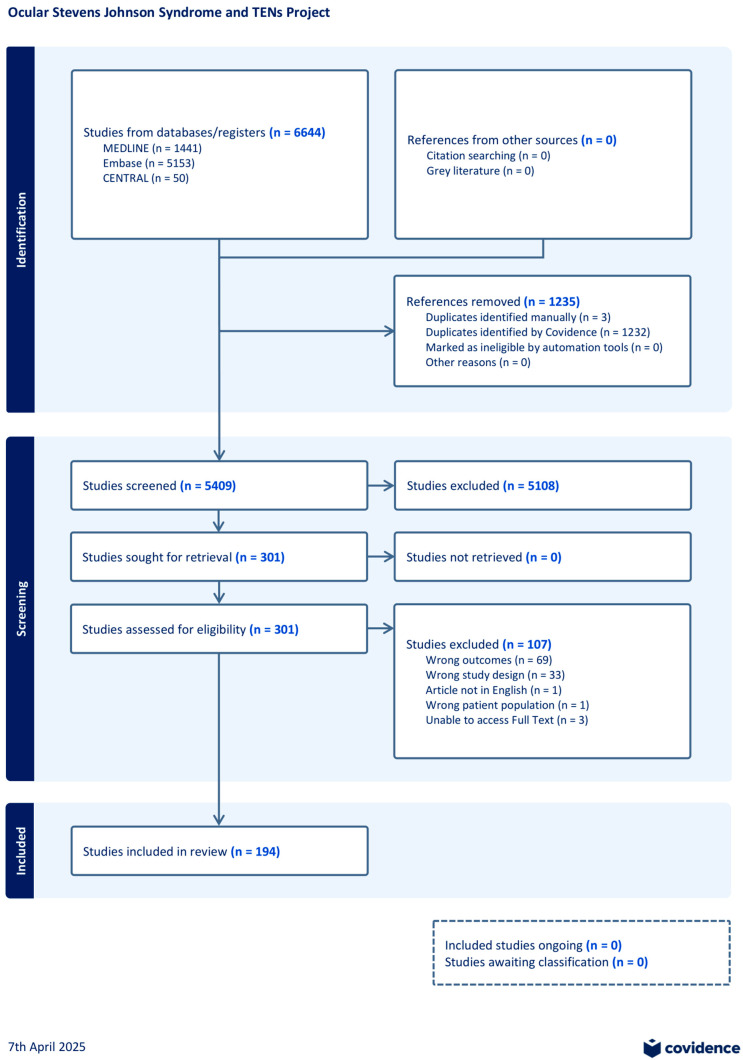
PRISMA flow diagram.

A total of 5409 unique studies were captured in our search, with 301 moving to full-text review; 5108 studies were excluded at the title and abstract stage because they did not focus on ophthalmic SJS/TEN or lacked measurable ocular outcomes. In total, 194 studies were included in the review (Figure 1) [7,23,24,25,26,27,28,29,30,31,32,33,34,35,36,37,38,39,40,41,42,43,44,45,46,47,48,49,50,51,52,53,54,55,56,57,58,59,60,61,62,63,64,65,66,67,68,69,70,71,72,73,74,75,76,77,78,79,80,81,82,83,84,85,86,87,88,89,90,91,92,93,94,95,96,97,98,99,100,101,102,103,104,105,106,107,108,109,110,111,112,113,114,115,116,117,118,119,120,121,122,123,124,125,126,127,128,129,130,131,132,133,134,135,136,137,138,139,140,141,142,143,144,145,146,147,148,149,150,151,152,153,154,155,156,157,158,159,160,161,162,163,164,165,166,167,168,169,170,171,172,173,174,175,176,177,178,179,180,181,182,183,184,185,186,187,188,189,190,191,192,193,194,195,196,197,198,199,200,201,202,203,204,205,206,207,208,209,210]. Key studies for each treatment modality are cited herein, while a full list of all included studies can be found in the Supplementary Materials (Supplementary Table S2). Supplementary Table S2 summarizes all studies investigating the management of ophthalmic manifestations of SJS and TEN from 1998 to 2024.

Table 1 summarizes the overall findings for each treatment modality. A total of 6698 eyes were treated, with BCVA improvement in 3500 eyes (52.2%), epithelial regeneration in 1126 eyes (16.8%), and symptom improvement in 1765 eyes (26.3%). Across studies, the ages of patients ranged from 2 months to 88.5 years. Study-level age distributions are presented in Supplementary Table S2. The most reported management modalities for ophthalmic SJS/TEN included topical therapy (n = 1424), mucosal grafts (n = 1220), contact lenses (n = 1134), amniotic membrane transplant (AMT) (n = 889), medical management (n = 524), and punctal occlusion (n = 456). Additional treatments included keratoprosthesis (n = 225), cultivated oral mucosal epithelial transplantation (COMET; n = 179), limbal stem cell transplant (LSCT; n = 154), IVIG (n = 142), intravenous (IV) steroids (n = 127), salivary gland transplantation (n = 70), TNF-alpha inhibitors (n = 66), lacrimal system irrigation (n = 42), 5-fluorouracil (n = 17), anti-vascular endothelial growth factor (anti-VEGF; n = 14), photodynamic therapy (PDT; n = 8), and debridement (n = 7).

Treatment options for ocular SJS/TEN depend on disease stage and severity [102]. Table 2 provides a comparison of treatment modalities for ocular manifestations and complications of SJS/TEN based on 194 studies. While certain modalities, such as topical therapy, are ubiquitous in the management of ocular SJS/TEN, the literature-pooled measures of treatment efficacy can be used to supplement clinician decision-making.

- Acute Treatment

Topical therapy (n = 1424 eyes) improved BCVA in 34.9% of eyes, although 26.8% experienced complications [57,164,181,206]. Medical management (n = 524 eyes) resulted in BCVA improvement in 39.5% of cases [76,81,149,160]. Parenteral medications such as IVIG and IV steroids have been used acutely. BCVA improvement was seen in only 18.3% of eyes treated with IVIG [20,44,64,163]. IV steroids (n = 127 eyes) improved BCVA in 40.1% of eyes and were associated with complications in 9.4% of eyes [96,181,202]. AMT (n = 889 eyes) improved BCVA in 70.2% of cases and promoted epithelial regeneration in 29.0%; however, complications occurred in 20.2% of eyes [22,159,183,204]. Debridement (n = 7) led to BCVA improvement in four of seven eyes [110,155].

- Chronic Treatment

Mucosal grafting (n = 1220) was the second most used modality and showed BCVA improvement in 38% and symptom relief in 43.5% of eyes [103,135,153,179]. Contact lenses were the third most used treatment (n = 1134 eyes) and improved BCVA in 90.3% and symptom relief in 43.4% of eyes [27,58,103,126,192]. Punctal occlusion (n = 456) showed visual improvement in 48% of eyes and epithelial regeneration in 27.4% [103,179]. Keratoprosthesis (n = 225) resulted in BCVA improvement in 76.9% of cases [103,109,122]. COMET (n = 179) improved BCVA in 51.4%, epithelial regeneration in 38.5%, and symptom improvement in 54.2% of eyes [45,70,86,94,196]. Limbal stem cell transplant (LSCT) (n = 154) led to BCVA improvement in 51.3%, epithelial regeneration in 53.2%, and symptom relief in 26.0% of cases [137,203]. Lastly, salivary gland transplantation (n = 70) demonstrated moderate rates of BCVA improvement (42.9%) and epithelial regeneration (30%) and high rates of symptom improvement (84.3%), although the small sample size limits interpretation [154,205].

- Emerging Treatments

Novel treatments for both the acute and chronic phases of ocular SJS/TEN have been investigated. These include TNF-alpha inhibitors during the acute stage or for cases of corneal melt secondary to ocular SJS/TEN, a combination of anti-VEGF and PDT therapy for patients with refractory corneal neovascularization, lacrimal system irrigation using corticosteroids for patients with epiphora and lacrimal duct obstruction, and lastly, 5-fluorouracil as an anti-fibrotic agent for conjunctival scarring and chronic dry eye [127,128,144,147,157,175,188,201,202].

- Quality Assessment

JBI critical appraisal checklists were used to perform the ROB assessment [211]. These checklists were chosen as they allow for consistent appraisal across a wide spectrum of study types included in our analysis. Item-level judgments (yes/no/unclear/not applicable) for each study are provided in Supplementary Table S3. The ranges of checklist items satisfied across study designs are as follows: 6/8 to 8/8 for case reports, 3/10 to 10/10 for case series, 4/8 for cross-sectional studies, 2/11 to 7/11 for cohort studies, 5/10 for case–control studies, 3/13 to 5/13 for RCTs, and 4/9 to 6/9 for NRCTs. Overall, case reports were generally at a low risk of bias. Case series, cohort studies, case–control studies, and non-randomized controlled trials demonstrated moderate-to-high risk of bias, with concerns related to selection and follow-up. The few RCTs available were at a high risk of bias. ROB results were not used to exclude studies.

## 4. Discussion

- Acute Treatment

Acutely, patients often receive topical therapy to provide adequate lubrication, reduce epithelial injury, prevent infection, and decrease inflammation. Topical therapies, most commonly in the form of lubricants, corticosteroids, and antibiotics, improved BCVA in a modest proportion of eyes and are typically used in combination with other treatment modalities [57,164,181,206]. Certain topical therapies, such as lubricating drops, may be used in the long term to manage chronic dry eye in SJS/TEN. Medical management with oral corticosteroids, antibiotics, and immunosuppressants helps to manage the systemic disease burden of SJS/TEN and limit the progression of mucocutaneous damage. Interestingly, medical management improved BCVA in a greater proportion of eyes compared to topical therapy (39.5% vs. 34.9%) [76,81,149,160]. Parenterally administered treatments such as IVIG, IV steroids, and TNF-alpha inhibitors have also been used. IVIG is thought to block the Fas–Fas ligand-mediated apoptosis of keratinocytes; however, BCVA improvement was seen in only 18.3% of eyes and was associated with complications such as corneal opacification and a higher incidence of ocular complications in pediatrics [20,44,64,163]. Indeed, a study by Kim et al. found that early intervention with IVIG improved BCVA and ocular symptoms in adults but not in pediatric patients [81]. Conversely, IV steroids improved BCVA in 40.1% of eyes and were associated with half as many complications as IVIG (9.4%) [96,181,202]. As such, the efficacy of systemic steroids, both oral and parenteral, should be considered and weighed against the increased risk of infection from immunosuppression [21]. TNF-alpha inhibitors such as infliximab and etanercept have been used in 66 eyes across five studies [147,157,175,201,202]. The largest of these studies, a post hoc analysis by Yan et al., revealed that eyes treated with etanercept (n = 58) had better outcomes than those treated with prednisolone in almost all Ocular Surface Grading Score (OSGS) categories, BCVA, and the Schirmer test, illustrating the utility of these corticosteroid-sparing medications.

Corneal and conjunctival epithelial defects are seen acutely in patients with moderate to very severe disease and can lead to sight-threatening chronic complications such as corneal neovascularization, opacification, ulceration, conjunctival keratinization, symblepharon formation, and limbal stem cell deficiency (LSCD) [102]. As such, prompt treatment with AMT, ideally within 7–10 days of the epithelial defect, promotes re-epithelialization and decreases the risk of chronic scarring sequelae [102]. AMT demonstrated strong visual and regenerative outcomes across studies. Although complications occurred in 20.2% of eyes, this was likely in part due to the severity and burden of disease in patients receiving AMT [22,159,183,204]. In total, 110 of the 889 AMT eyes received a special type of AMT called ProKera, a sutureless amniotic membrane graft clipped into a symblepharon ring system. In this subgroup of patients, ProKera improved BCVA in 88.2% of cases when used alone or with systemic corticosteroids, subconjunctival triamcinolone acetonide, and a scleral shell (“Triple Ten” therapy). An advantage of this procedure is that it does not require the use of general anesthetic and may be completed at the bedside. However, it showed limited impact on peripheral conjunctival inflammation that was not covered by the ProKera device, suggesting a higher efficacy in milder cases of SJS/TEN [61,87,134,177].

Alternative treatments for acute ocular manifestations of SJS/TEN have been considered. Most patients with ocular SJS/TEN experience dry eye; however, a smaller proportion experience epiphora secondary to nasolacrimal duct occlusion. A study by Xiang et al. found that lacrimal system irrigation with dexamethasone drops significantly decreased lacrimal passage obstruction and rates of epiphora in 42 eyes of patients with chronic SJS/TEN [127]. As such, this route of administration of corticosteroids may be useful for this subset of patients.

Lastly, pseudomembrane formation in the acute phase can predispose patients to symblepharon formation. As such, debridement is an important part of preventing long-term complications. Debridement (n = 7) led to BCVA improvement in four of seven eyes [110,155].

- Chronic Treatment

Patients surviving the acute phase of SJS/TEN are still at risk of developing chronic ocular sequelae such as lid margin keratinization, limbal stem cell deficiency (LSCD), symblepharon formation, trichiasis, lacrimal and meibomian gland dysfunction, dry eye, corneal neovascularization, and conjunctivalization 6 months after the onset of acute symptoms [7]. The incidence of chronic ocular SJS/TEN is 21–29% in pediatric patients and 27–59% in adult patients, and it is thought to be caused by persistent ocular surface inflammation and repetitive ocular surface trauma from adnexal changes [100,103,212].

Lid margin keratinization (LMK) occurs secondary to posterior migration of the mucocutaneous junction approximately three months after the acute phase. Untreated LMK causes progressive corneal microtrauma and damage. Mucosal grafting is a definitive treatment for LMK that involves the surgical removal of the keratinized lid margin and transplant of healthy mucosal tissue [213]. Mucosal grafting showed BCVA improvement in 38% and symptom relief in 43.5% of eyes [103,135,153,179]. Conversely, contact lenses do not directly address the underlying pathology of chronic SJS/TEN but, rather, prevent damage by acting as a shield between the lid margin and the cornea. Use of contact lenses resulted in the highest proportion of eyes with improved BCVA (90.3%) and symptom relief in 43.4% of eyes [27,58,103,126,192]. It is important to note that although the majority of therapeutic contact lenses are used in the chronic phase of ocular SJS/TEN, certain lenses, like bandage contact lenses, are used acutely. Despite high efficacy, disadvantages of contact lenses include high cost and a complex fitting process that can result in lower patient compliance [214].

Loss of POV precipitates LSCD, corneal conjunctivalization, vascularization, and opacification in chronic SJS/TEN. In cases of LSCD, LSCT may be performed. LSCT improved BCVA and regenerated epithelium in approximately half of all treated eyes and provided symptom relief in 26.0% of cases [137,203]. There are limitations of LSCT. In unilateral cases, harvesting healthy limbal stem cells from the contralateral eye can potentially induce LSCD [215]. In bilateral cases, allogenic LSCT requires long-term immunosuppression, increasing the risk of infection and malignancy. COMET is another treatment for LSCD and is a useful alternative to LSCT, particularly in patients with bilateral disease where no healthy autologous limbal tissue is available for transplantation. First described by Nakamura et al. in 2004, COMET involves transplanting autologous oral epithelial cells grown on an amniotic membrane onto the affected eye [45]. As COMET uses autologous tissue, there is no need for continuous immunosuppression [45,70,86,94,196]. Patients with advanced LSCD who have refractory corneal neovascularization may benefit from a combination of anti-VEGF and photodynamic therapy (PDT). In 2019, Yoon et al. investigated the use of PDT with verteporfin and anti-VEGF (bevacizumab) in eight eyes of SJS/TEN patients who had corneal neovascularization refractory to topical corticosteroids [128]. Complete remission of corneal neovascularization was achieved in five eyes and partial remission in three eyes after six months of treatment, suggesting potential efficacy of combined treatment for refractory patients [128].

Lacrimal and meibomian gland secretions are necessary for adequate tear production, as they produce the aqueous and lipid portion of tears, respectively. Dysfunction of these glands, often seen in chronic SJS/TEN, results in aqueous deficiency and evaporative dry eye [216,217,218]. Punctal occlusion with either punctal plugs or cautery prevents tear drainage through the nasolacrimal duct, ensuring an adequate tear film over the ocular surface. Across studies involving punctal occlusion, vision improved in 48% and epithelial regeneration in 27.4% of eyes, suggesting a role in ocular surface stabilization [103,179]. Another option to treat dry eye is salivary gland transplantation, whereby salivary glands are grafted within the fornices [216]. Salivary gland transplantation demonstrated moderate rates of BCVA improvement, epithelial regeneration, and high rates of symptom improvement (84.3%), although the small sample size limits interpretation [154,205]. A newer non-surgical treatment for dry eye and conjunctival scarring in ocular SJS/TEN involves injections of the anti-metabolite 5-fluorouracil into either the fornices or the subconjunctival palpebral lobe. Therapy with 5-fluorouracil is thought to act by inducing apoptosis of fibroblasts and preventing their proliferation, thereby decreasing the scarring around the lacrimal gland and conjunctiva. 5-Fluorouracil improved visual function and ocular surface disease index (OSDI) scores, and decreased corneal scarring, across 17 eyes of SJS/TEN patients in two studies [144,188].

Despite the numerous treatment options available, some patients experience severe refractory ocular surface changes. Visual rehabilitation using keratoprosthesis is a useful option for patients with end-stage ocular SJS/TEN. Across studies, keratoprosthesis demonstrated strong visual rehabilitation, with BCVA improvement in 76.9% of cases [103,109,122].

We must share several study limitations. Firstly, across studies, and particularly in case reports, patients frequently received more than one treatment modality, which may complicate the attribution of BCVA improvement, symptom relief, or epithelial regeneration to a single treatment. However, a multimodal strategy, particularly when initiated early, was associated with better visual and ocular surface outcomes [147,149,159]. Additionally, as some eyes received multiple treatments, they were counted in more than one treatment group. This overlap may complicate the interpretation of treatment-specific outcomes, although the descriptive nature of our synthesis reduces the risk of misleading statistical inference. Secondly, outcome definitions and reporting varied across studies. For example, “symptom improvement” or “complications” were often not standardized, introducing bias into what is considered to constitute symptom improvement or a complication. Similarly, interpretation of pooled results is limited by heterogeneity in the timing of outcome assessment, with outcomes such as BCVA, epithelial regeneration, and symptom improvement measured at different follow-up intervals. A further limitation is that outcomes may be confounded by differences in baseline disease severity and duration. For example, keratoprosthesis is typically used in advanced ocular SJS/TEN, where visual prognosis is inherently poor. Thus, observed outcomes may not solely represent the effect of the intervention but may also reflect underlying disease severity and duration. Data on age and gender subgroups were insufficiently reported, precluding any meaningful assessment of whether treatment outcomes differed between children and adults or between males and females. Lastly, certain novel treatments, such as 5-fluorouracil, anti-VEGF, and salivary gland transplantation, had small sample sizes. Although these options may be promising, larger sample sizes are necessary to increase confidence in these modalities.

In conclusion, the findings of this systematic review underscore the importance of early identification and comprehensive intervention in managing ocular complications of SJS/TEN. Clinicians should tailor treatment to disease stage and individual patient characteristics, with an emphasis on preventing chronic sequelae through prompt intervention. Patients may benefit from a discussion on all available and potentially synergistic treatment modalities.

## Figures and Tables

**Table 1:** Summary of 194 studies investigating the management of ocular manifestations and complications of SJS/TEN.

Treatment Modality	Total N (Eyes)	BCVA Improvement (n = Eyes)	Epithelial Regeneration	Improved Ocular Symptoms	Reported Complications (n = Eyes)
Topical Treatment (corticosteroids, antibiotics, lubricants, beta blockers, maternal serum drops, plasma drops, retinol, vitamin A, Wharton’s jelly drops, cyclosporine)	1424	497 (34.9%)	263 (18.5%)	264 (18.5%)	382 (26.8%)
Mucosal Graft	1220	464 (38%)	139 (11.4%)	531 (43.5%)	24 (2%)
Contact Lens	1134	1024 (90.3%)	71 (6.3%)	492 (43.4%)	55 (4.9%)
Amniotic Membrane Transplantation (AMT)	889 (110 ProKera)	624 (70.2%; 97 ProKera)	258 (29%; 22 ProKera)	181 (20.4%; 18 ProKera)	180 (20.2%; 5 ProKera)
Medical Management (oral corticosteroids, oral immunosuppressants, oral antihistamines, oral antibiotics)	524	207 (39.5%)	43 (8.2%)	36 (6.9%)	63 (12%)
Punctal Occlusion	456	219 (48%)	125 (27.4%)	-	2 (0.4%)
Keratoprosthesis	225	173 (76.9%)	-	-	43 (19.1%)
Cultivated Oral Mucosal Epithelial Transplantation (COMET)	179	92 (51.4%)	69 (38.5%)	97 (54.2%)	11 (6.1%)
Limbal Stem Cell Transplant	154	79 (51.3%)	82 (53.2%)	40 (26%)	35 (22.7%)
Intravenous Immunoglobulin (IVIG)	142	26 (18.3%)	12 (8.5%)	24 (16.9%)	26 (18.3%)
IV Steroids	127	51 (40.2%)	35 (27.6%)	2 (1.6%)	12 (9.4%)
Salivary Gland Transplantation	70	30 (42.9%)	21 (30%)	59 (84.3%)	-
TNF-Alpha Inhibitor	66	62 (93.9%)	64 (97%)	-	2 (3%)
Lacrimal System Irrigation	42	-	-	-	4 (9.5%)
5-Fluorouracil	17	-	-	17 (100%)	-
Anti-VEGF	14	6 (42.9%)	-	14 (100%)	2 (14.3%)
Photodynamic Therapy (PDT)	8	-	-	8 (100%)	2 (25%)
Debridement	7	4 (57.1%)	2 (28.6%)	-	2 (28.6%)

**Table 2:** Comparison of treatment modalities for ocular manifestations and complications of SJS/TEN based on 194 studies.

Treatment Modality	SJS/TEN Stage	Indications	Efficacy	Potential Complications
Amniotic Membrane Transplantation (AMT)	Acute	To promote epithelial regeneration and healing of corneal defects	BCVA improvement = 70.2% Epithelial regeneration = 29.0% Symptom improvement = 20.4%	Membrane displacement, infection, persistent epithelial defect, corneal melt, scarring, conjunctivalization
Debridement	Acute	Prevention of adhesions via removal of fibrin, pseudomembranes, and necrotic epithelium from the ocular surface	BCVA improved in 4 of 7 eyes and promoted epithelial regeneration in 2 of 7 eyes	Mechanical trauma, inflammation, subconjunctival hemorrhage
Intravenous Immunoglobulin (IVIG)	Acute	Manage systemic disease burden and limit progression of mucocutaneous damage	BCVA improvement = 18.3% Epithelial regeneration = 8.5% Symptom improvement = 16.9%	Thromboembolic events, renal dysfunction, hemolytic anemia, infusion reactions (fever, headache, nausea, hypotension)
IV Steroids	Acute	Reduce systemic inflammation and limit progression of mucocutaneous damage	BCVA improvement = 40.1% Epithelial regeneration = 27.6% Symptom improvement = 1.6%	↑ Risk of infection, impaired wound healing, GI bleeding, insomnia, mood alteration, osteoporosis, ↑ IOP/glaucoma, cataract
Lacrimal System Irrigation	Acute	Epiphora secondary to nasolacrimal duct occlusion	Significantly decrease lacrimal passage obstruction and rates of epiphora	Not reported
Medical Management	Acute	Manage systemic disease burden and limit progression of mucocutaneous damage	BCVA improvement = 39.5% Epithelial regeneration = 8.2% Symptom improvement = 6.9%	Adverse drug reactions
Contact Lens	Acute and Chronic	Severe dry eye, enhancing corneal healing, and protecting the ocular surface from damage caused by sequelae of SJS/TEN (e.g., posterior eyelid margin changes, trichiasis, distichiasis, and other adnexal changes)	BCVA improvement = 90.3% Epithelial regeneration = 6.3% Symptom improvement = 43.4%	Infection, corneal hypoxia, corneal neovascularization, epithelial defects (abrasion)
TNF-Alpha Inhibitor	Acute and Chronic	1. Acute SJS/TEN as an alternative to corticosteroids 2. Corneal melt	Etanercept-treated eyes demonstrated superior outcomes compared to those treated with prednisolone in nearly all aspects of the OSGS, BCVA, and Schirmer test	Infection, ↑ risk of malignancy, infusion site reaction, cytopenia
Topical Treatment	Acute and Chronic	To provide adequate lubrication, reduce epithelial injury, prevent infection, and decrease inflammation	BCVA improvement = 34.9% Epithelial regeneration = 18.5% Symptom improvement = 18.5%	Overall minimal Steroid: ↑ IOP, cataract with prolonged use ABX: microbial resistance with prolonged use
Anti-VEGF + Photodynamic Therapy (PDT)	Chronic	Refractory corneal neovascularization	At 3 and 6 months after treatment, all eyes showed regression of corneal neovascularization; complete regression was achieved in five eyes (62.5%) and partial regression in three eyes (37.5%)	Corneal edema, hemorrhage around neovascularization
Cultivated Oral Mucosal Epithelial Transplantation (COMET)	Chronic	Limbal stem cell deficiency	BCVA improvement = 51.4% Epithelial regeneration = 38.5% Symptom improvement = 54.2%	Graft failure, infection, recurrent LSCD, persistent epithelial defect, corneal neovascularization
Keratoprosthesis	Chronic	Visual rehabilitation in end-stage cases with ocular surface changes such as severe corneal opacification, conjunctivalization, or keratinization refractory to other treatments	BCVA improvement = 76.9% Epithelial regeneration = not reported Symptom improvement = not reported	Retroprosthetic membrane (RPM), elevated intraocular pressure/glaucoma, endophthalmitis, retinal detachment (RD), device extrusion
Limbal Stem Cell Transplant	Chronic	Limbal stem cell deficiency	BCVA improvement = 51.3% Epithelial regeneration = 53.2% Symptom improvement = 26.0%	Graft rejection/failure, infection, recurrent LSCD, persistent epithelial defect, corneal neovascularization
Mucosal Graft	Chronic	Lid margin keratinization	BCVA improvement = 38.0% Epithelial regeneration = 11.4% Symptom improvement = 43.5%	Graft failure, graft displacement, infection, scarring, persistent keratinization
Punctal Occlusion	Chronic	Dry eye secondary to meibomian/lacrimal gland dysfunction	BCVA improvement = 48% Epithelial regeneration = 27.4% Symptom improvement = Not reported	Epiphora, infection (especially with punctal plugs), extrusion (punctal plugs), recanalization
Salivary Gland Transplantation	Chronic	Dry eye secondary to meibomian/lacrimal gland dysfunction	BCVA improvement = 42.9% Epithelial regeneration = 30% Symptom improvement = 84.3%	Graft failure, infection, epiphora
5-Fluorouracil	Chronic	Dry eye secondary to conjunctival scarring of lacrimal glands located in the supertemporal fornix	Improved visual acuity, ocular surface disease index (OSDI) scores, and decreased corneal scarring	Not reported

## Data Availability

Available upon reasonable request to the corresponding author. The original data presented in this study are openly available in Medline, Embase, and CENTRAL.

## Supplementary Materials

The following supporting information can be downloaded at https://www.mdpi.com/article/10.3390/vision9030078/s1: Table S1: Search strategy and results; Table S2: Study characteristics for all 194 included studies; Table S3: Risk-of-bias assessments.
