# Supplementary material for: Stevens–Johnson Syndrome and Toxic Epidermal Necrolysis: A Systematic Review of Ophthalmic Management and Treatment
Source: Vision (Basel). 2025 Sep 11;9(3):78. doi: 10.3390/vision9030078 (PMC12452647; doi:10.3390/vision9030078)
Supplement: Supplementary file 1 [file vision-09-00078-s001.zip › vision-3830431-supplementary/Table S1-edited.pdf]

Supplementary Table S1: Search strategy for Medline, Embase, and CENTRAL, with results per query indicated. The entire search strategy was included in the final table row. Search Date: March 1, 2025

Entry	Medline	Embase	CENTRAL
1: exp Stevens-Johnson Syndrome/ or Stevens Johnson Syndrome.mp. or toxic epidermal necrolysis.mp.	8767	20295	255
2: exp Eye Diseases/ or exp Orbital Diseases/ or exp Conjunctivitis/ or exp Keratitis/ or exp Dry Eye Syndromes/ or exp Uveitis/ or ophthalmic.mp. or ocular.mp. or orbital.mp. or conjunctiva*.mp. or cornea*.mp. or keratitis.mp. or blepharitis.mp. or meibomian.mp. or limbal stem cell.mp. or symblepharon.mp. or trichiasis.mp. or vision.mp. or visual acuity.mp. or dry eye.mp. or tear film.mp.	1044316	1668213	69434
3: 1 and 2	1441	5153	50

**Entire Search Strategy:**	[exp Stevens-Johnson Syndrome/ or Stevens Johnson Syndrome.mp. or toxic epidermal necrolysis.mp.] AND [exp Eye Diseases/ or exp Orbital Diseases/ or exp Conjunctivitis/ or exp Keratitis/ or exp Dry Eye Syndromes/ or exp Uveitis/ or ophthalmic.mp. or ocular.mp. or orbital.mp. or conjunctiva*.mp. or cornea*.mp. or keratitis.mp. or blepharitis.mp. or meibomian.mp. or limbal stem cell.mp. or symblepharon.mp. or trichiasis.mp. or vision.mp. or visual acuity.mp. or dry eye.mp. or tear film.mp.]	['stevens johnson syndrome'/exp OR 'stevens johnson syndrome' OR 'toxic epidermal necrolysis'/exp OR 'toxic epidermal necrolysis'] AND ['eye disease'/exp OR 'eye disease' OR 'orbit disease'/exp OR 'orbit disease' OR ophthalmic OR 'orbital'/exp OR orbital OR 'ocular'/exp OR 'ocular' OR 'conjunctivitis'/exp OR 'conjunctivitis' OR 'keratitis'/exp OR 'keratitis' OR 'dry eye syndrome'/exp OR 'dry eye syndrome' OR (conjunctiva* OR cornea* OR keratitis OR blepharitis OR meibomian OR limbal stem cell OR symblepharon OR trichiasis OR vision OR visual acuity OR dry eye OR tear film):ti, ab, kw]	['stevens-johnson syndrome' (MeSH) OR 'stevens johnson syndrome' OR 'toxic epidermal necrolysis' (MeSH) OR 'toxic epidermal necrolysis'] AND ['eye disease' (MeSH) OR 'eye disease' OR 'orbit disease' (MeSH) OR 'orbit disease' OR ophthalmic OR 'orbit' (MeSH) OR orbital OR 'ocular' OR 'conjunctivitis' (MeSH) OR 'conjunctivitis' OR 'keratitis' (MeSH) OR 'keratitis' OR 'dry eye syndrome' (MeSH) OR 'dry eye syndrome' OR (conjunctiva* OR cornea* OR keratitis OR blepharitis OR meibomian OR limbal stem cell OR symblepharon OR trichiasis OR vision OR visual acuity OR dry eye OR tear film):ti, ab, kw]
