# Supplementary material for: Stevens–Johnson Syndrome and Toxic Epidermal Necrolysis: A Systematic Review of Ophthalmic Management and Treatment
Source: Vision (Basel). 2025 Sep 11;9(3):78. doi: 10.3390/vision9030078 (PMC12452647; doi:10.3390/vision9030078)
Supplement: Supplementary file 1 [file vision-09-00078-s001.zip › vision-3830431-supplementary/Table S2-edited.pdf]

Supplementary File: **Stevens–Johnson Syndrome and Toxic Epidermal Necrolysis: A Systematic Review of Ophthalmic Management and Treatment**

Supplementary Table S2. Studies comparing ophthalmic management and treatment methods of SJS and TEN, in chronological order (n = 194).

**Study ID**	**Design**	**Patient Demographics**	**Treatments**	**Results**
Tseng et al. 1998[1]	Prospective case series	SJS 2 patients (2 eyes)<br><br>TEN: 1 patient (2 eyes)<br><br>Mean Age: 31	AMT with transplantation (ALT)<br>AMT with ALT and penetrating keratoplasty (PKP)	Significant vision improvement in all eyes (>3 lines on the Snellen chart).<br><br>One eye developed acute irreversible PKP rejection with bacterial graft ulcer at 17 months and had a repeat PKP.
Dua et al. 1999[2]	Interventional case series	SJS: 1 patient (1 eye)<br><br>Age: 31	LSCT<br>medical management (tacrolimus)	The corneal surface was completely epithelialized within 2 weeks, and there was a substantial improvement in vision and symptoms.
Rao et al. 1999[3]	Retrospective non-comparative case series.	SJS: 2 patients (2 eyes)<br><br>Mean Age: 38.5	Limbal stem cell transplant	BCVA (worsened in 1 eye and stayed the same in the other).<br><br>At Presentation: CF (both patients).<br><br>At Follow-Up: CF, LP.<br><br>Ocular discomfort improved in both patients, and corneal vascularization decreased.<br><br>Epithelial healing occurred in one patient at day 19 post-operation.
Tsubota et al. 1999[4]	Case series	SJS: 4 patients (5 eyes)<br><br>Mean Age: 9 years	LSCT + AMT	BCVA improved in 3 eyes.<br><br>Cornea epithelialized in 3 eyes.<br><br>Treatment failure occurred in 2 eyes.
Honavar et al. 2000[5]	Prospective interventional case series	SJS: 10 patients (10 eyes)<br><br>Mean Age: 22 ± 11.8 years	AMT	BCVA<br>At Presentation: In nine eyes there was accurate projection of rays; it was 20/200 in one eye.<br><br>At Follow-Up: Visual acuity improved in six of nine (66.7%) eyes with accurate projection of rays preoperatively to counting fingers at 1 to 3 m; it remained at the preoperative level in three eyes with total corneal opacification. One eye with preoperative 20/200 vision improved to 20/50 at the final follow-up.<br><br>Complete corneal reepithelization occurred in all eyes between 1 and 6 weeks.<br><br>One patient had a central corneal melt.

Romero-Rangel et al. 2000[6]	Prospective and retrospective case series	SJS: 33 patients (54 eyes)<br><br>TEN: 2 patients (4 eyes)<br><br>Mean Age: 44.6 ± 20.5	Gas-permeable scleral contact lens therapy	Improvement in best corrected visual acuity (gain of 2 or more Snellen lines) observed in 53% of eyes.<br><br>53% of eyes with active corneal epithelial defects at the time of lens fitting had their defects healed.<br><br>92% of patients reported improvement in their quality of life as a result of reduction in photophobia and discomfort.
Rosenthal et al. 2000[7]	Retrospective chart review	SJS: 6 patients (7 eyes)<br><br>Mean Age: 19.7	Contact lens	Persistent corneal epithelial defects of five of the seven eyes with Stevens–Johnson syndrome re-epithelialized.
Shimazaki et al. 2000[8]	Retrospective non-comparative case series	SJS: 29 eyes<br><br>Mean Age: 35.7 ± 20.7 years	AMT<br><br>LSCT<br><br>Topical therapy (steroid, ABX, lubricant)	The ocular surface was successfully covered by corneal epithelium in 13 eyes (44.8%).<br><br>Fourteen eyes (48.3%) improved their visual acuity by more than 2 lines on the Snellen acuity chart. The mean corrected visual acuity recovered from 0.0039 to 0.017.<br><br>PED was the most common complication, which developed in 12 eyes.
Daya et al. 2001[9]	Retrospective non-comparative case series	SJS: 3 patients (3 eyes)<br><br>Mean Age: 42.7	LSCT<br><br>Medical management (cyclosporine)	BCVA improved in all 3 eyes.<br>Cornea epithelialized in all 3 eyes by 11 days post-op.<br><br>One patient experienced punctate epithelial keratopathy, and one patient experienced corneal vascularization.
Koizumi et al. 2001[10]	Retrospective non-comparative case series	SJS: 7 eyes<br><br>Mean Age: 28.2	Limbal stem cell transplant<br><br>AMT	Visual acuity improved in all eyes after surgery.<br><br>All eyes were free from epithelial defects 48 hours after surgery.<br><br>One patient (2 eyes) experienced rejection 6 months postoperatively. The definition of epithelial rejection used here was the sudden onset of corneal epithelial damage with conjunctival inflammation.
Tappin et al. 2001[11]	Case series	SJS: 1 patient (1 eye)<br><br>Age: 49 years	Scleral lens	BCVA:<br>At Presentation: 6/24.<br>At Follow-Up: 6/12.<br><br>The patient reported a significant subjective improvement in her sight and a sustained reduction in the symptoms.
John et al. 2002[12]	Interventional case series	TEN: 2 patients (4 eyes)<br><br>Mean Age: 7 years	AMT	Patient 1: At follow-up, there was no symblepharon, and there was good ocular uncorrected bilateral vision of 20/20.<br><br>Patient 2: At 34 months' follow-up, the vision was 20/30 OD and 20/40 OS.
Samson et al. 2002[13]	Retrospective case series	6 patients (8 eyes) with SJS<br><br>Mean Age: 19.2 years	Limbal stem cell transplantation (LSCT) from HLA-matched living donors<br><br>Autologous LSCT	Long-term corneal stability was achieved in 50% of the SJS study eyes.<br><br>Prognosis of LSCT in patients with SJS is significantly worse than in patients with other ocular disease causes.<br><br>45% LSCT failure rate due to microbial infection,

				graft rejection, or both.
Shimakazi et al. 2002[14]	Non-comparative retrospective interventional case series	SJS: 8 patients (8 eyes)<br><br>Mean Age; 44.3 years	LSCT<br><br>AMT	BCVA improved in 6 of 8 eyes.<br><br>Cornea epithelialized in 3 of 8 eyes.
Solomon et al. 2002[15]	Retrospective non-comparative interventional case series	SJS: 3 patients (3 eyes)<br><br>Mean Age: 42.7 years	AMT	BCVA (improved in 2 of 3 eyes):<br>At Presentation: HM, HM, 20/60.<br>At Follow-Up: CF (1 foot), HM, 20/40.<br><br>All 3 AMT procedures were successful (surgical success was defined as the cessation of aqueous leak, formation of a deep chamber, complete epithelialization of the AM's outermost layer, and formation of a visible stromal thickness at the operated site by the first month of follow-up).<br><br>One patient experienced a secondary ulcer due to an exposed suture.
Tsubota et al. 2002[16]	Case report	SJS: 1 patient (1 eye)<br><br>Age: 9 years	LSCT<br><br>Medical management (cyclosporine)<br><br>Topical therapy (autologous serum, steroid)	BCVA improved from HM to 20/800.<br>Cornea epithelialized in one month.
Geerling et al. 2003[17]	Case report	SJS: 1 patient (2 eyes)<br><br>Age: 29 years	Keratoprosthesis (OOKP; 1 eye)<br><br>Salivary gland transplant (1 eye)	BCVA (improved in one eye):<br>At Presentation: CF OD and HM OS.<br>At Follow-Up: 6/6 and N12 unaided OS.<br><br>Dry eye symptoms improved significantly in the right eye.
Gomes et al. 2003[18]	Prospective non-comparative interventional case series	SJS: 10 patients (10 eyes)<br><br>Mean Age: 30.5 years	Limbal stem cell transplant<br><br>AMT	Satisfactory ocular surface reconstruction was obtained in two eyes, with reduced inflammation and vascularization and a mean epithelialization time of 3 weeks.<br><br>Surgical failure was observed in four eyes and complications in four eyes.<br><br>Visual acuity improved in four eyes, remained stable in five eyes, and decreased in one eye.
Park et al. 2003[19]	Case report	SJS: 1 patient (2 eyes)<br><br>Age: 28 years	AMT	The graft completely epithelialized, and 3-month follow-up showed no further signs of keratinization, with improved ocular irritation.
Solomon et al. 2003[20]	Non-comparative interventional case series	SJS/TEN: 2 patients (3 eyes)<br><br>Mean Age: 43.5 years	AMT for reconstruction of the conjunctival fornices	Focal keratinization and corneal epithelial defect (1 eye), re-operation 2.5 years with AMT (1 eye) and 1 recurrence.<br><br>Improved visual acuity in all 3 eyes (>2 lines on the Snellen chart).<br><br>Low AMT efficacy in SJS/ TEN compared to trauma-induced symblepharon due to diffuse conjunctival keratinization or conjunctival epithelial stem cell depletion.
Goldberg et al. 2004[21]	Interventional case report	SJS: 1 patient (2 eyes)<br><br>Age: 62 years	Medical Management: Oral steroid<br><br>Topical therapy	On presentation, vision was 20/200 OU.<br><br>Despite aggressive medical management, the patient developed corneal melting, membranous conjunctivitis, persistent episcleritis, and

			(steroid, ABX, lubricant)	symblepharon formation, followed by chronic symblepharons, corneal neovascularization, and cicatricial ectropions.
Kaido et al. 2004[22]	Retrospective non-comparative case series	SJS: 18 patients (31 eyes)<br><br>Mean Age: 39.6 ± 17.5 years	Punctal occlusion	Mean logarithmic VA showed significant improvement, from 0.64 0.87 to 0.52 0.86 (P 0.05).<br><br>Symptomatic improvement was observed in 19 eyes.
Lam et al. 2004[23]	Retrospective case series<br><br>75% of SJS and 100% of TEN was induced by drugs	SJS: 8 patients<br><br>SJS/TEN: 2 patients<br><br>TEN: 1 patient<br><br>Mean Age: not reported	Steroid and supportive care (2 pts)<br><br>Steroid, antihistamines, and supportive care (4 pts)<br><br>Antihistamine and supportive care (1 pt)<br><br>IVIG and steroid (3 pts)<br><br>Antihistamine, surgical debridement, and supportive care (1 pt)	Early use of the short-term IV or prednisolone 1-2mg/kg/day for 3-5 days lacked any significant side effects or increase in mortality and morbidity.<br><br>Use of IVIG, IVIG and steroids, or surgical debridement and supportive care was reported to be satisfactory in the treatment of SJS and TEN.<br><br>75% of SJS and 100% of TEN cases were induced by drugs: most commonly by carbamazepine within the first 2 weeks of treatment.
Nakamura et al. 2004[24]	Prospective interventional case series	SJS: 2 patients (3 eyes)<br><br>Mean Age: 19 years	COMET<br><br>AMT<br><br>Topical therapy (steroid, ABX)	BCVA improved in all 3 eyes (postoperative visual acuity improved by two or more lines).<br><br>Cornea epithelialized within 48 hours of treatment.
Fukuda et al. 2005[25]	Case report	SJS: 1 patient (1 eye)<br><br>Age: 49	Keratoprosthesis (OOKP)	Decimal BCVA improved from HM OS to 1.2 OS at 1.5 months after the surgery.
Yip et al. 2005[26]	Retrospective historically controlled study	Total: 28 TEN patients divided into two groups<br><br>IVIG group: 8 patients (16 eyes)<br><br>No IVIG group (historical cohort): 15 patients (30 eyes)<br><br>Mean Age: 54.6 ± 19.9 years	IVIG (16 eyes)<br><br>Systemic steroids (14 eyes)<br><br>Topical therapy (steroid, ABX, lubricant)	8 patients in the IVIG group had ocular complications.<br>8 patients in the no-IVIG group had ocular complications.<br><br>There was no difference in the severity of visually significant ocular complications between the treatment and historical cohorts (P = 0.1198).
Kobayashi et al. 2006[27]	Case report	TEN: 1 patient (2 eyes)	AMT	Complete corneal and conjunctival epithelialization was observed 14 days after amniotic membrane transplantation in the left eye, and after 28 days in the right eye.

		Age: 6 years		Corrected visual acuity improved to 20/16 in both eyes 6 months postoperatively.<br><br>Minimal symblepharon and peripheral scarring were observed only in the right eye.
Nakamura et al. 2006[28]	Retrospective non-comparative case series.	SJS: 2 patients (2 eyes)<br><br>Mean Age: 30 years	Limbal stem cell transplant<br><br>AMT<br><br>Topical therapy (steroid, ABX)	Cornea epithelialized withing 5 days of treatment<br><br>All eyes demonstrated an improvement in visual acuity by ≥ 2 lines.<br><br>During the follow-up period, the corneal surface of all patients remained stable and transparent, without significant complications
Ang et al. 2007[29]	Case report	SJS: 1 patient (2 eyes)<br><br>Age: 32 years	Limbal stem cell transplant (LSCT)<br><br>Topical therapy (ABX, steroid)<br><br>Medical management (systemic steroid, immunosuppressant)	Complete corneal epithelialization was achieved 48 hours after cultivated LSCT (OD) and 3 weeks after conventional LSCT (OS)<br><br>BCVA at presentation was 20/40 OU<br>Four years after surgery, visual acuity was 20/30 in the right eye and 20/100 in the left eye, with more severe corneal scarring and opacification noted in the left eye.
Chang et al. 2007[30]	Retrospective chart review	SJS: 83 patients (166 eyes)<br><br>TEN: 30 patients (60 eyes)<br><br>Mean Age: 47.8 ± 23.8	Topical therapy (188 eyes)<br><br>Debridement (2 eyes)<br><br>Contact lenses (2 eyes)	Ocular sequelae occurred during the 3-month follow-up<br>period after 23 of the 128 attacks with ocular manifestations (23 patients).
De Rojas et al. 2007[31]	Retrospective case series	SJS: 24 patients<br><br>TEN: 10 patients<br><br>30 patients total (60 eyes)<br><br>Median Age: 38 years	Systemic immunosuppressive therapy (systemic corticosteroids and steroid-sparing immunosuppressive agents): prednisolone (12 patients), intravenous methylprednisolone (2 patients), dapsone (4 patients), sulphapyridine (1 patient), azathioprine (5 patients), mycophenolate (5 patients), adalimumab (1 patient), ciclosporin (5 patients), infliximab (2 patients), and cyclophosphamide (1 patient)	Immunosuppressive therapy successfully controlled inflammation in 10 patients with SJS with mucous membrane pemphigoid (MMP), SJS with recurrent episodic inflammation, SJS with scleritis, and TEN.<br><br>Visual acuity did not change in 16 eyes, increased in 19 eyes, and decreased in 21 eyes.
Tandon et al. 2007[32]	Case report	TEN: 1 patient (2 eyes)	AMT	After 12 weeks, the eyelid skin appeared well healed, and Snellen visual acuities were 6/6 OU.

		Age: 12 years		There were no conjunctival adhesions other than a small amount of symblepharon in the inferolateral aspect of OD and mild foreshortening of both inferior fornices.<br><br>The tear-film breakup time appeared to be within normal limits.<br><br>The inferior limbus of each cornea showed keratinization and punctate keratopathy.
Yip et al. 2007[33]	Retrospective cohort study	Total: 44 patients (88 eyes)<br><br>Mean Age: 52.2 ± 18.6 years	Systemic steroid (32 patients)<br><br>Topical therapy (steroid, ABX, lubricant) (37 patients)	Complications:<br><br>Severe dry eyes (20 patients);<br>Trichiasis (7 patients);<br>Symblepharon (6 patients);<br>Distichiasis (6 patients);<br>Visual loss (2 patients);<br>Entropion (2 patients);<br>Ankyloblepharon (1 patient);<br>Lagophthalmos (1 patient);<br>Cornea ulceration (1 patient).<br><br>The use of topical antibiotic was found to be a significant risk factor for late ocular complications.<br><br>Patients with dry eye were more likely to have used topical antibiotics.
Jain et al. 2008[34]	Case report	SJS: 1 patient (2 eyes)<br><br>Age: 35 years	Topical therapy (lubricant)<br><br>AMT	BCVA:<br>At Presentation: 20/200 OD and 20/400 OS.<br>At Follow-Up (2 months): 20/40 OD and 20/60 OS.<br><br>Cornea epithelialized within 10 days of treatment.
Sayegh et al. 2008[35]	Retrospective non-comparative interventional case series.	SJS: 15 patients (16 eyes)<br><br>Mean Age: 50 ± 18 years	Keratoprosthesis	Twelve eyes (75%) achieved a visual acuity of 20/200 or better after surgery. Visual acuity was maintained at 20/200 or better over a mean period of 2.5 2.0 years.<br><br>At last follow-up, seven eyes (44%) retained good-to-excellent vision of 20/70 or better.<br><br>Five eyes that had initial improvements in visual acuity subsequently deteriorated to less than 20/200 vision because of the following complications: corneal melting and leak in one eye, retinal detachment in two eyes, end-stage glaucoma in one eye, and vitreous hemorrhage in one eye.
Uy et al. 2008[36]	Retrospective interventional case series	SJS: 2 patients (3 eyes)<br><br>Mean Age: 46.5 years	Anti-VEGF (bevacizumab)	Both patients completed the 3-month observation period<br>and reported that it significantly improved ocular comfort. At the end of the study period, visual acuity improved in all 3 eyes; all eyes were observed to have decreased ocular surface neovascularization, corneal opacification, and conjunctival injection. No serious adverse events were reported.
Araki et al. 2009[37]	Prospective case series	SJS/TEN: 5 patients (10 eyes)<br><br>Mean Age: 32.8 years	Intravenous pulse therapy with methylprednisolone (steroid pulse therapy; 500 or 1000 mg/day for 3 to 4 days)	Skin eruptions dramatically improved after steroid pulse therapy.<br><br>Pseudomembranes disappeared and conjunctival epithelium regenerated within 6 weeks.<br><br>Five eyes showed superficial punctuate keratopathy.<br><br>All eyes had clear corneas with POV in the chronic stage.<br><br>One eye had slight fornix shortening.
Sotozono et al.	Cross-sectional	Total: 94 patients (188	Topical steroids during the first	Coryzal symptoms preceded skin eruptions in 75 patients; acute conjunctivitis, oral involvement, and

2009[38]	study	eyes)<br><br>Steroid Group: 33 patients (66 eyes)<br><br>No-Treatment Group: 31 patients (62 eyes)<br><br>Indeterminate Group: 30 patients (60 eyes)<br><br>Mean Age: 41.6 ± 18.5 years	week of the acute stage	fingernail loss occurred in all patients.<br><br>Visual outcomes were significantly better in the group receiving topical steroids (33/ 94) compared with those of the no-treatment group (P<0.00001).<br><br>Topical steroid treatment from disease onset seems to be important for the improvement of visual prognosis.
Tougeron-Brousseau et al. 2009[39]	Retrospective case series	SJS/TEN: 39 patients (67 eyes)<br><br>Mean Age: 35.8 ± 13.9 years	Scleral lenses	Scleral lens fitting failed in 3 patients.<br><br>The mean follow-up time was 33.3 ± 17.6 months, and 36 patients had improvement in the visual acuity of their better eye (from 0.73 to 0.50 logMAR; P = 0.0001) 6 months after scleral lens placement. Among fitted patients, VA in the better eye (36 eyes) progressed from 0.73 to 0.50 logMAR (P = 0.0001) 6 months after scleral lens fitting. Among fitted patients, VA in the worse eye (28 eyes) progressed from 0.86 to 0.54 logMAR (P = 0.0001) 6 months after scleral lens fitting.<br><br>Mean ocular surface disease index (OSDI) improved from 76.9 ± 22.8 to 37.1 ± 26.7 (P = 0.0001).<br><br>No serious adverse events attributable to the scleral lenses occurred.
Iyer et al. 2010[40]	Retrospective case series	SJS: 31 patients (54 eyes)<br><br>Mean Age: 21.3 years	Mucous membrane grafting (MMG)	In 50 of 54 eyes (92.6%), there was improvement or stabilization in patient comfort, conjunctival hyperemia, ocular surface staining characteristics, and best corrected visual acuity over a mean follow-up period of 6 months.
Marinho et al. 2010[41]	Prospective interventional case series	SJS: 14 patients (14 eyes)<br><br>Mean Age: 36 years	Salivary gland transplant	All patients expressed improvement in their ocular discomfort. Nine eyes showed a slight best corrected visual acuity improvement.
Shammas et al. 2010[42]	Retrospective interventional case series	SJS/TEN: 6 patients (12 eyes)<br><br>Mean Age: 34.7	Sutureless AMT(ProKera) and short-term use of intensive corticosteroid medication or ProKera alone	Four patients whose entire ocular surface was treated with AM retained visual acuities of 20/40 or better and an intact ocular surface.<br><br>The two patients treated with only a ProKera device or unsutured AM sheets developed more significant ocular surface abnormalities, and 1 developed a corneal perforation.<br><br>All patients exhibited good ocular comfort with minimal symptoms of photophobia, and all have resumed their normal activities.<br><br>10 eyes epithelialized.

Shay et al. 2010[43]	Case report	TEN: 1 patient (2 eyes)<br><br>Age: 5 years	ProKera	Complete re-epithelialization, clear corneas, and 20/20 BCVA at 7 months with residual conjunctival inflammation, trichiasis, and entropion formation.
Das et al. 2011[44]	Case report	SJS: 1 patient (2 eyes)<br><br>Age: 18 years	MMG<br>Topical therapy (steroid, ABX)<br>Medical management (ABX, NSAID)	BCVA:<br><br>At Presentation: Light Perception OU.<br>At Follow-Up: 20/80 OU.<br><br>The re-epithelialization of the corneal epithelium was completed by 10 days.
Finkelstein et al. 2011[45]	Retrospective case series	SJS/TEN:<br>Systemic ABX: 37 patients<br>Systemic Corticosteroids: 22 patients<br>Antivirals: 15 patients<br>IVIG: 21 patients<br>Corticosteroids Plus IVIG: 8 patients<br><br>Mean Age: 9.6 ± 4.8 years	Systemic antibiotics<br>Systemic corticosteroids<br>Antiviral drugs<br>IVIG<br>Corticosteroids plus IVIG	Children administered with IVIG had a higher incidence of ocular complications compared to those who were not.
Gregory, D.G 2011[46]	Prospective case series	SJS/TEN: 10 patients (20 eyes)<br><br>Mean Age: 16.2 years	AMT during acute phase of SJS or TEN (first 10 days), and repeated every 10-14 days if there was persistent epithelial sloughing/ocular surface inflammation	All patients had best corrected visual acuity of ≥20/30, with 9 of the 10 seeing 20/20. Dry eye was moderate or less in all patients.<br><br>Overall ocular surface and eyelid scarring was mild to moderate in all patients.<br><br>All pts had epithelialized corneas at follow-up.
Lau et al. 2011[47]	Case report	SJS: 1 patient (2 eyes)<br><br>Age: 49 years	AMT<br><br>Topical therapy (autologous serum, steroid, ABX)<br><br>Punctal occlusion<br><br>Bandage contact lens	UVA at Presentation: 20/125 OD and CF OS.<br><br>BCVA at Follow-Up: 20/50 OD and 20/200 OS.<br><br>Cornea epithelialized in both eyes.
Liu et al. 2011[48]	Retrospective case series	SJS: 1 patient (1 eye) with total limbal stem cell deficiency due to SJS<br><br>Age: 29 years	Autologous oral mucosal graft + AMT	In 30 ± 19.8 months, pain and photophobia were resolved, a stable epithelium with regressed corneal vascularization was exhibited, and visual acuity improved.
Onaran et al. 2011[49]	Case report	SJS: 1 patient (2 eyes)<br><br>Age: 20 years	IV corticosteroid<br><br>Topical therapy (steroid, ABX, lubricant, cyclosporine)	Reepithelization occurred after 14 days.<br><br>Patient recovered without any severe ocular complications. The final ophthalmic examination in the 4th month revealed bilateral visual acuity of 20/20, clear cornea, Schirmer test value >15 mm, and only 2 mm of symblepharon in the lower fornix

			IVIG	laterally.
Pujari et al. 2011[50]	Retrospective interventional case series	29 eyes (11 patients, 12 eyes) with corneal blindness secondary to SJS/ TEN<br><br>Mean Age: 50.5 ± 18.2 years	Boston keratoprosthesis type II	Visual acuity after surgery improved to 20/200 or better in 23 eyes (79.3%), and to 20/30 or better in 10 eyes (34.5%).<br><br>In patients with at least 1 year of follow-up, visual acuity of 20/200 or better was maintained in 12 eyes (57.1%) (8 had BCVA of 20/200 or better in the SJS/TEN cohort).<br><br>One eye developed endophthalmitis and one had a retinal detachment following treatment in the SJS/ TEN cohort).
Satake et al. 2011[51]	Non-comparative retrospective interventional case series	SJS: 12 eyes<br><br>Mean Age: 58.5 years	Cultivated oral mucosal epithelial sheet transplantation (COMET)<br><br>Topical therapy (steroid, ABX, lubricant, autologous serum)<br><br>Medical management (oral steroid)	Epithelial defects resolved in 8 eyes post-COMET.<br><br>Epithelial defects persisted in 3 eyes post-COMET.<br><br>One eye experienced fibrovascular tissue invasion.<br><br>Fornix reconstruction was successful in 4 eyes and recurred in 2 eyes.
Sc et al. 2011[52]	Case report	SJS: 1 patient (1 eye)<br><br>Age: 35 years	Keratoprosthesis (OOPK	BCVA improved from HM OD to 6/6 OD
Uy et al. 2011[53]	Case report	SJS: 1 patient (1 eye)<br><br>Age: 40 years	Anti-VEGF (bevacizumab)	Visual acuity in the bevacizumab-treated eye improved from 20/40 to 20/25.<br><br>The bevacizumab-treated eye was observed to have gradual improvement in ocular comfort, accompanied by a decrease in ocular surface neovascularization and conjunctival injection.
Yagi et al. 2011[54]	Case report	SJS: 1 patient (2 eyes)<br><br>Age: 59 years	Medical management (steroid pulse therapy)	After steroid pulse therapy, our patient's general condition, including her skin rash, had markedly improved, and serum cytokine levels decreased coordinately.<br><br>Conjunctival epithelium began to regenerate and healed completely by day 23.<br><br>Her visual acuity 2 years after disease onset was 20/20 in both eyes, and no cicatricial changes remained.
Barua et al. 2012[55]	Case report	TEN: 1 patient (2 eyes)<br><br>Age: 15 months	AMT<br>IV steroid<br>IV ABX<br>Topical therapy (steroid, ABX, lubricant)	At 22 months' follow-up there were no signs of delay in the patient's visual development and apparent visual acuity of at least 6/9 (equivalent with Kay's picture charts) with both eyes open, evidence of binocular vision, and stereopsis.<br><br>Mild punctate staining was present OS.<br><br>Lower tarsal conjunctiva of both eyelids showed mild scarring.
Hess et al. 2012[56]	Case report	SJS: 1 patient (2 eyes)<br><br>Age: 44 years	AMT<br>IV steroid<br>IV antibiotics<br>Topical therapy (ABX, steroid)	BCVA<br><br>At Presentation: Counting fingers OU.

				At Follow-Up (1 Year): 20/25 OU.<br><br>Re-epithelialization occurred within 48 hours OU.<br><br>Biomicroscopy showed no signs of corneal vascularization or scarring. Fornices showed no signs of symblepharon. There were no signs of trichiasis, and the patient had no pain or photophobia.
Hsu et al. 2012[57]	Retrospective case–control study	Medical Management: 17 patients (33 eyes)<br><br>AMT: 13 patients (25 eyes)<br><br>Mean Age: Not reported	Medical management (33 eyes)<br>AMT in acute SJS and TEN	1 of 23 eyes with moderate or severe (i.e., epithelial defects greater than or up to 50% of cornea, moderate-to-severe conjunctival injection, and extensive pseudomembranes) presentation treated with early AMT (4.3%) resulted in a poor outcome (i.e., < 20/200 BCVA with persistent corneal irritation) within 3 months, compared with 8 of 23 eyes (34.8%) that were medically managed (P=0.022).
Huang et al. 2012[58]	Retrospective observational case series	SJS: 7 patients (7 eyes)<br><br>Median Age: 67 years	Keratoprosthesis	BCVA:<br><br>At Presentation: Hand motion (4 eyes), light perception (3 eyes).<br><br>At Follow-Up: Increased in 6/7 eyes (20/25, 20/70, LP, 20/100, 20/40, 20/80, 20/100).<br><br>Notably, visual acuity decreased over follow-up. At the final follow-up, VA was (20/100, 20/80, NLP, NLP, 20/100, 20/80, 20/100).
Kesarwani et al. 2012[59]	Case report	SJS: 1 patient (2 eyes)<br><br>Age: 14 years	Anti-VEGF (bevacizumab)<br><br>Topical therapy (steroid, ABX, lubricant)	BCVA:<br><br>At Presentation: Light perception OD and counting fingers OS.<br><br>At Follow-Up: 20/125 OD and 20/100 OS.<br><br>Improvement in symptoms bilaterally.
Sant'Anna et al. 2012[60]	Prospective, non-comparative interventional case series	SJS: 19 patients (19 eyes)<br><br>Mean Age: 31.5 ± 13.9 years	MMG + minor salivary gland transplantation<br><br>Topical therapy (steroid, ABX)	BCVA improved significantly in eight eyes.<br><br>The symptoms questionnaire revealed improvement in foreign body sensation in 53.6% of the patients, in photophobia in 50.2%, and in pain in 54.8%.<br><br>Corneal transparency improved significantly in 11 (72.2%) eyes, and corneal neovascularization improved significantly in 5 eyes (29.4%).
Ciralsky et al. 2013[61]	Retrospective case series	SJS: 1 patient (2 eyes)<br><br>Age: 70 years	Application of AMT during the hyperacute phase (<72 hours) in the left eye and in the acute phase (day 6) in the right eye	AMT destabilized the inflammatory process and enhanced re-epithelialization of the ocular surface in the left eye.<br><br>The right eye (treated later in the course of disease) required additional surgical procedures and exhibited more advanced ocular surface pathology, eye pain, and visual difficulties than the left.
Kim et al. 2013[62]	Retrospective observational case series	Total: 51 patients (102 eyes) divided into two groups<br><br>Pediatric Group: 17	Systemic steroid (9 pediatric and 30 adult patients)<br><br>IVIG (9 pediatric and 9 adult patients)	At the final follow-up, the logMAR and ocular involvement scores (OISs) had improved significantly in the adult group (p = 0.0002, p = 0.023, respectively), but not in the pediatric group.<br><br>Early intervention with IVIG (6 patients) or corticosteroids (14 patients) significantly improved the mean BCVA and OIS in the adult group (p =

		patients (34 eyes)<br><br>Adult Group: 34 patients (68 eyes)<br><br>Mean Age Pediatric Group: 7.5 ± 4.8<br><br>Mean Age Adult Group: 46.2 ± 14.2	AMT (4 pediatric and 5 adult patients)	0.043 and p = 0.024, respectively, for IVIG; p = 0.002 and p = 0.034, respectively, for corticosteroid).<br><br>AMT was found to be associated with a significantly improved BCVA or OIS in the late treatment group (3 patients) or the group with a better initial OIS (2 patients) (p = 0.043 and p = 0.043, respectively, for BCVA; p = 0.042 and p = 0.041, respectively, for OIS).
Kolomeyer et al. 2013[63]	Case report	SJS: 1 patient (2 eyes)<br><br>Age: 19 years	ProKera	Complete healing; no complications.
Ling et al. 2013[64]	Retrospective case series	SJS: 1 patient (1 eye)<br><br>Age: 54 years	Scleral lens (PROSE)	BCVA (remained the same):<br><br>At Presentation: 20/80.<br><br>At Follow-Up: 20/80.<br><br>Subjective and objective improvement was observed after treatment with PROSE.<br><br>Epithelial defects healed within 1 month of starting PROSE.
Md Noh et al. 2013[65]	Case report	SJS: 1 patient (2 eyes)<br><br>Age: 42 years	AMT<br><br>Topical therapy (steroid, ABX)<br><br>Medical management (oral steroid)	BCVA :<br><br>At Presentation: LP OD and HM OS.<br><br>At Follow-Up: 1/60 OD and 6/60 OS.<br><br>Patient exhibited non-healing epithelial defect (2 eyes).
Prabhasawat et al. 2013[66]	Prospective non-comparative interventional case series.	SJS: 30 patients (59 eyes)<br><br>Mean Age: 48 years	Topical therapy (cyclosporine)	Seventeen patients (56.67%) completed the study. All 17 cases demonstrated significant improvement in dry eye symptoms, conjunctival injection, corneal staining, Schirmer I test, and FCT (P < 0.05).<br><br>Eight patients withdrew from the study because of intolerable side effects of CsA, which included pain, redness, and eyelid swelling.<br><br>Five cases were lost to follow-up.
Sotozono et al. 2013[67]	Non-comparative retrospective interventional case series	SJS: 21 eyes<br><br>Median Age: 43 years	COMET	In SJS, logMAR BCVA improved significantly during the 24 weeks after surgery.<br><br>The ocular surface grading score also improved significantly at 4, 12, and 24 weeks after surgery.
Tomlins et al. 2013[68]	Prospective case series	TEN: 2 patients (4 eyes)<br><br>Mean Age: 31.5 years	“Triple TEN” Management: (1) subconjunctival triamcinolone administered to each fornix, (2) placement of AM mounted on a	Ocular surface inflammation resolved within 4 weeks, with no progression of conjunctival cicatrization.<br><br>No evidence of conjunctival scarring or limbal stem cell failure or recurrence at the 1-year follow-up.<br><br>No long-term complications of the Triple-TEN

			ProKera, and (3) insertion of a steeply curved acrylic sclera shell spacer to vault the lids away from the globe and prevent symblepharon formation	regimen.
Basu et al. 2014[69]	Case series	SJS: 2 patients (2 eyes)<br><br>Mean Age: 23.5	MMG + keratoprosthesis	Patient 1: BCVA improved from HM OS to 20/20 OS.<br><br>Patient 2: BCVA improved from LP OD to 20/20 OD.
De Oliveira et al. 2014[70]	Prospective interventional case series	SJS: 4 patients (4 eyes)<br><br>Mean Age: Not reported	Keratoprosthesis (Boston Type 1)<br><br>Topical therapy (steroid, ABX)	BCVA improved in all 4 eyes treated with keratoprosthesis.<br><br>Each of the 4 patients experienced a postoperative complication.<br><br>One retroprosthetic membrane, one corneal melt, one KPro extrusion, one glaucoma.
Heur et al. 2014[71]	Retrospective interventional case series	16 patients (27 eyes)<br><br>Mean Age: 40.3 ± 11.2 years	Prosthetic Replacement of the Ocular Surface Ecosystem (PROSE) scleral lens fitting	Significant improvements in mean visual acuity and ocular surface disease index (OSDI) scores were achieved.
Iyer et al. 2014[72]	Non-comparative retrospective interventional case series.	SJS: 232 patients (464 eyes)<br><br>Mean Age: 26.7 years	Punctal occlusion (160 eyes)<br><br>MMG (238 eyes)<br><br>Fornix reconstruction (24 eyes; 17 AMT, 4 MMG, 6 COMET)<br><br>Minor salivary gland transplant (MSGT: 3 eyes)<br><br>PROSE lens (36 eyes)<br><br>Keratoprosthesis (47 eyes)	BCVA improved in 59 eyes in the punctal occlusion group.<br><br>BCVA improved in 91 eyes in the MMG group.<br><br>BCVA improved in 12 eyes in the fornix reconstruction group.<br><br>Symptom improvement in 3 eyes and BCVA improvement in 1 eye in the MSGT group.<br><br>BCVA improved in 23 eyes in the PROSE group.<br><br>BCVA improved in 34 eyes in the keratoprosthesis group.
López-García et al. 2014[73]	Prospective interventional case series	TEN: 5 patients (9 eyes)<br><br>Mean Age: 41.2 ± 13.2 years	AMT	The VA, foreign body sensation, and corneal findings such as epithelial defects, periphery neovascularization, and tear instability improved significantly during the study (p = 0.006).<br><br>The ocular surface re-epithelialized in all patients, and the ulcers on eyelid skin and eyelid margins healed without scars after the second week.<br><br>At one-year follow-up, 2 eyes with severe involvement in the acute phase showed mild peripheral corneal neovascularization, and 1 eye showed a corneal epithelial defect.
Pruet et	Case report	SJS: 1 patient	Novel Technique: Symblepharon lysis	20/20 vision was maintained bilaterally.

al. 2014[74]		(2 eyes)<br><br>Age: 27 years	followed by placement of AM wrapped around a symblepharon ring	No recurrent symblepharon apart from temporal canthus.
Sotozono et al. 2014[75]	Non-comparative, retrospective, interventional case series	Total: 42 patients (53 eyes) divided into 3 groups based on BCVA<br><br>BCVA worse than 20/2000 (11 eyes)<br><br>BCVA ranging from 20/200 to 20/2000 (31 eyes)<br><br>BCVA of 20/200 or better (11 eyes)<br><br>Mean Age: 51.8 ± 13.9 years	Tear-exchangeable limbal rigid contact lens	BCVA improved from 1.61 to 0.86 logMAR at 3 months after fitting CL use.<br><br>Improvement in BCVA in groups 1, 2, and 3 was 0.95 logMAR, 0.82 logMAR, and 0.37 logMAR, respectively.<br><br>Mean 25-item National Eye Institute Visual Function Questionnaire composite score for the 11 subscales improved from 37.6 ± 16.0 to 58.4 ± 17.4 (P = 0.000001).<br><br>No serious adverse events attributable to limbal CL use occurred.
Sotozono et al. 2014[76]	Retrospective Interventional case series	SJS: 3 patients (3 eyes)<br><br>Mean Age: 33.3 years	COMET<br><br>Medical management (steroid, cyclosporine)<br><br>Topical therapy (steroid, ABX, lubricant)<br><br>Contact lens	BCVA improved in 2 of 3 eyes.<br><br>Complete epithelialization occurred in the three eyes by the 24-week follow-up.
Wang et al. 2014[77]	Case series	SJS: 10 patients (10 eyes)<br><br>Mean Age: 48.8 years	Modified small tectonic keratoplasty (MSTK) with minimal corneal graft<br><br>IV steroids<br><br>Topical therapy (steroid, ABX, lubricant)	Visual acuity improved in six eyes (60%), unchanged in three eyes (30%), and declined in one eye (10%).<br><br>Corneal perforation healed and global integrity was achieved in all eyes.<br><br>Retraction of conjunctival flap occurred in one eye, which was treated with an additional procedure.
Agrawal et al. 2015[78]	Prospective interventional case series	SJS: 8 patients (14 eyes)<br><br>Mean Age: 34 years	AMT<br><br>Topical therapy (steroid, ABX, lubricant)	In all fourteen eyes of eight patients, the amniotic membrane uptake was satisfactory, with significant reduction in inflammation and symptomatic relief from dry eye.
Alexander et al. 2015[79]	Retrospective comparative consecutive case series	SJS: 26 patients (27 eye)<br><br>Mean Age: 50	Keratoprosthesis	BCVA:<br><br>At Presentation: < 20/200 (all eyes).

		years		At Follow-Up: ≥ 20/200 (22 eyes).<br><br>In patients with SJS, repeat keratoprosthesis implantation was a significantly more common indication than in patients without SJS (33% vs. 8%, P < 0.001).<br><br>Retention failure occurred in a significantly higher percentage of eyes in patients with SJS (52%) than in eyes of patients without SJS (15%; P < 0.001).<br><br>Several postoperative complications were more common in patients with SJS, including sterile corneal stromal necrosis (59% vs. 8%, P < 0.001), persistent corneal epithelial defects (59% vs. 24%, P < 0.001), and suspected and confirmed infectious corneal infiltrates (30% vs. 10%, P = 0.009).
Papakostas et al. 2015[80]	Retrospective cohort study	SJS/TEN: 86 patients (167 eyes)<br><br>Median Age: 36 years	Prosthetic Replacement of the Ocular Surface Ecosystem (PROSE) scleral lens fitting	Significant improvements in median visual acuity and NEI VFQ-25 scores were noted.
Catt et al. 2016[81]	Retrospective case series	Total: 36 patients (20 SJS, 7 TEN, 9 SJS/TEN)<br><br>Mean Age: 8.8 years	Topical therapy (lubricant, steroid; 25 patients)<br><br>AMT (3patients)<br><br>PROSE (4 patients)<br><br>Punctal occlusion (1 patient)	Over 90% of children maintained a visual acuity of 20/40 or better in each eye at a mean follow-up of 1.4 years (only 3 patients did not achieve 20/40 or better in each eye).
Cheung et al. 2016[82]	Case report	SJS/TEN: 1 patient (2 eyes)<br><br>Age: 61 years	Novel technique using AM suture-fixated onto custom-designed symblepharon rings	VA was 20/20 in right eye and 20/25 in left eye. Both eyes were noted to have small symblephara temporally.<br><br>No epithelial defects at follow-up.
Gregory, D.G., 2016[83]	Prospective case series	Total: 79 patients (158 eyes)<br><br>Mild + moderate: 41 patients (82 eyes)<br><br>Severe + extremely severe: 38 patients (76 eyes)<br><br>Mean Age: Not reported	Mild and Moderate Cases: Topical therapy (steroid, ABX, lubricant)<br><br>Severe and Extremely Severe Cases: Topical therapy + AMT	Mild and Moderate Cases:<br><br>BCVA of 20/20 in all 82 eyes with no dry eye symptoms or scarring sequelae at 3-month follow-up.<br><br>Severe and Extremely Severe Cases:<br><br>BCVA of 20/20 in 74 eyes, with mild–moderate tarsal scarring in 16 eyes and moderate dry eye in 6 eyes.<br><br>All severe or extremely severe cases had epithelial defects at presentation. No epithelial defects were noted at follow-up.<br><br>Symptom improvement in 152 eyes (no dry eye symptoms at follow up).
Iyer et al. 2016[84]	Retrospective chart review	Punctal Cautery: 231 eyes<br>MMG: 393	Group II (2005-2015)<br>Punctal cautery<br>MMG	Ocular surface stabilization was beneficial in preventing chronic ocular deterioration.<br><br>In end-stage disease, visual rehabilitation can be

		eyes<br>PROSE Lens: 46 eyes<br>Keratoprosthesis: 61 eyes<br><br>Mean Age: 27.4 ± 24.7 years	PROSE lens<br>Keratoprosthesis	accomplished via keratoprosthesis.<br><br>Improved BCVA in 129 eyes in the MMG group; 347 had improved symptoms in the MMG group; 130 eyes in the MMG group had improved fluorescein staining.<br><br>Improved BCVA in 106 eyes in the punctal occlusion group; 123 eyes in the punctal occlusion group had improved fluorescein staining.<br>Improved BCVA in 32 eyes in the PROSE group (all patients had improved symptoms).<br><br>Improved BCVA in 40 eyes in the keratoprosthesis group.
La Porta Weber et al. 2016[85]	Prospective interventional case series	SJS: 22 eyes<br><br>Mean Age: 39.5 ± 12.2 years	Scleral contact lens	All patients who were fitted with SCLs had an improved BCVA, defined as a gain of 2 or more Snellen lines.<br><br>Ocular surface symptoms assessed by OSDI score were<br>significantly better after 12 months of SCL wear<br>($P < .001$).
Ma et al. 2016[86]	Retrospective interventional case series	SJS: 9 patients (9 eyes)<br><br>Mean Age: 11.4 years	Novel surgical technique using a single large sheet of AM and a custom-made forniceal ring for AMT vs. traditional technique of AMT using multiple pieces of AM	All eyes showed stability or improvement in visual acuity and in their ocular exam findings at final follow-up.<br><br>8/9 patients had epithelial defects at presentation that re-epithelialized by the end of follow-up (Table 1).
Sharma et al. 2016[87]	Prospective randomized controlled clinical trial	50 patients (100 eyes)<br><br>AMT + MT Group = 25 patients<br><br>MT Alone Group = 25 patients<br><br>Mean Age AMT Group: 31.7 ± 16.7 years<br><br>Mean Age MT Group: 27.9 ± 12.5 years	AMT + medical therapy (MT) vs. medical therapy alone	Compared to the eyes in the MT group, AMT+MT eyes achieved better mean BCVA, longer mean TBUT, greater mean Schirmer test results, and better ocular surface outcomes.
Ahmad et al. 2017[88]	Retrospective interventional case series	SJS: 31 patients (61 eyes)<br><br>Median Age: 10 years	AMT (two types, some patients received both)<br><br>Amniograft (30 patients)<br><br>ProKera (26 patients)<br><br>Topical therapy (steroid, ABX, lubricant,	BCVA (improved in 22 eyes):<br><br>At Presentation: 31/61 eyes > 20/40, 30/61 eyes < 20/40.<br><br>At Follow-Up: 53/61 eyes > 20/40, 8/61 eyes < 20/40.<br><br>There were no adverse effects related to AMT in either group.

			cyclosporine)	
Ma et al. 2017[89]	Retrospective case series	SJS: 13 patients (14 eyes)<br><br>Mean Age: 61.5 ± 17.3 years	Keratoprosthesis	Thirteen eyes (92.9%) achieved a best corrected visual acuity of 20/200 or better, and eight eyes (57.1%) achieved a best corrected visual acuity of 20/40 or better, after surgery.<br><br>However, 71.4% (10/14) experienced visual decline because of different postoperative complications. Common complications included corneal melting (10/14), glaucoma (3/14), vitritis (5/14), superficial tissue overgrowth (2/14), and retroprosthetic membrane (4/14).
Pinna et al. 2017[90]	Retrospective interventional case series	TEN: 9 patients (18 eyes)<br><br>Mean Age: 63.8 ± 24.7 years	Plasmapheresis and human IVIg	The use of plasmapheresis and human IVIg in severe acute TEN with ocular complications may be lifesaving and contributes to the reduction in ocular surface inflammation.
Barry et al. 2018[91]	Case report	TEN: 1 patient (2 eyes)<br><br>Age: 38 years	Ocular lubricants, topical steroids, topical antimicrobial therapy, bilateral ProKera AMT	Despite intensive topical therapy, the patient developed bilateral complications (symblepharon, ankyloblepharon, shortening of the inferior conjunctival fornix OS).<br><br>Following ProKera AMT, current Snellen visual acuity is 6/9 right eye and 6/6 left eye.
Basu et al. 2018[92]	Retrospective comparative case series	284 pediatric patients (568 eyes) categorized into 4 groups<br><br>- No keratopathy (NK)<br><br>- Non-lid-related keratopathy (NLRK)<br><br>-Lid-related keratopathy (LRK)<br><br>- End-stage keratopathy (ESK)<br><br>440 eyes were treated conservatively<br><br>128 eyes received therapeutic treatment<br><br>Mean Age: Not reported	Conservative<br><br>PROSE<br><br>PROSE/MMG<br><br>Allo-SLET/ keratoprosthesis	For patients managed conservatively, BCVA decreased significantly over time in all groups except those with ESK.<br><br>NLRK Group:<br><br>PROSE lenses (36 eyes) significantly improved BCVA in the NLRK group. BCVA remained stable at a median of 20/30, compared to 20/60 in the control group.<br><br>LRK Group:<br><br>PROSE lenses (33 eyes), BCVA improved from a median of 20/100 to 20/50 in the LRK group compared to 20/100 in the control group; P =.002.<br><br>MMG (39 eyes): There was greater improvement in median BCVA with MMG, from a median of 20/100 to 20/32 (P < .0001).<br><br>MMG followed by PROSE (19 eyes): An even greater degree of improvement in BCVA was noted, from a median of 20/100 to 20/25 (P < .0001).<br><br>ESK Group:<br><br>Allo-SLET (9 eyes): Median BCVA improved from hand motions to 20/320 in the Allo-SLET (9 eyes) group.<br><br>LVP KPro (11 eyes): median BCVA improved from

				hand motions to 20/100 (11 eyes) (P < .0001).
Chan et al. 2018[93]	Case report	SJS: 1 patient (2 eyes)<br><br>Age: 23 years	Debridement of corneal epithelium, bandage contact lenses, topical ABX (moxifloxacin), topical steroid (prednisolone)	BCVA Presentation: 20/150 OD and 20/200 OS.<br><br>BCVA Follow-Up: 20/30 OD and 20/25 OS.<br><br>Cornea epithelialized on day 3 of treatment.
Filippi et al. 2018[94]	Case report	SJS: 2 patients (4 eyes)<br><br>Mean Age: 6 years	Topical propranolol	Patient 1:<br><br>BCVA Presentation: Snellen visual acuity 4/10 OU.<br><br>BCVA Follow-Up: 6/10 OD and 7/10 OS.<br><br>Significant reduction in photophobia, corneal neovascular congestion, and eyelid hyperemia.<br><br>Patient 2:<br><br>BCVA could not be measured due to corneal leukoma and calcification.<br><br>Significant reduction in photophobia.
Gu et al. 2018[95]	Case report	SJS: 1 patient (1 eye)<br><br>Age: 50 years	Keratoprosthesis (Boston Type 1)	Postoperatively, the patient had a VA of 20/100, which was maintained for 12 months with Boston Type 1.<br><br>Prior treatment for SJS included autologous submandibular gland transplantation and punctal occlusion.
Kalhorn et al. 2018[96]	Case series	SJS: 1 patient (1 eye)<br><br>Age: 2 years	Maternal serum drops<br><br>Previous treatment AMT + topical lubricant	Persistent epithelial defect, which was present for 7 weeks prior to treatment, resolved on day 17 of treatment with maternal serum drops.<br><br>BCVA Presentation: Light perception.<br><br>BCVA Follow-Up (5 months): Light perception.
Nguyen et al. 2018[97]	Retrospective, observational case series	SJS: 1 patient (1 eye)<br><br>Age: 21 years	Scleral lens (Eye Print Pro)	Pt had reduction in vision and severe conjunctival<br><br>chemosis and hyperemia with habitual scleral lens.<br><br>Patient's conjunctivalization over corneal surface remained stable over follow-up period.<br><br>BCVA Presentation: 20/60.<br><br>BCVA Follow-Up: 20/30.
Osaki et al. 2018[98]	Retrospective interventional case series	SJS: 40 eyes (63.5% of patients)<br><br>Mean Age: 44.7 ± 21.5 years	Labial mucous membrane graft (MMG)	All eyes had stable or improved visual acuity 1 month postoperatively except for 1 patient.<br><br>97.7% of patients reported improvement of the ocular<br><br>hyperemia and foreign body sensation after the procedure.

				Partial resolution of the upper eyelid margin<br><br>to anatomic position, with good aesthetic appearance and relief of foreign body sensation, was observed in the most severe patients, and all of those were secondary to SJS (17.5%).<br><br>During the follow-up period, there was recurrence of entropion in four SJS patients (recurrence 6 and 12 months after the procedure).<br><br>No postoperative infection, failure of graft survival, or other complications were observed in this series.
Sato et al. 2018[99]	Retrospective case series	SJS: 9 patients<br><br>TEN: 3 patients<br><br>Mean Age: 8.4 years	Oral steroids (9 SJS and 1 TEN patients)<br><br>Steroid pulse therapy (2 TEN patients)<br><br>IVIG (1 SJS and 3 TEN patients)<br><br>Oral cyclosporine (1 TEN patient)<br><br>Plasmapheresis (1 TEN patient)	SJS: 8 patients recovered with no complications; 1 patient had chronic dry eye.<br><br>TEN: 1 patient recovered with no complications; 1 patient had chronic conjunctival scarring, trichiasis; and 1 patient had severe corneal injury.
Sevik et al. 2018[100]	Prospective interventional case series	SJS: 1 patient (2 eyes)<br><br>Age: 50 years	Topical regenerating agent (RGTA; Calcicol)<br><br>Previous treatment topical antibiotics and artificial tears	Epithelial defect persisted for 15 days. RGTA was added to treatment regimen, and the epithelial defect was completely healed in 8 days.
Bas et al. 2019[101]	Case report	TEN: 1 patient (2 eyes)<br><br>Age: 12 months	AMT (sutureless technique using a symblepharon ring)	Cornea completely epithelized by post-treatment week 6 in the right eye and week 8 in the left eye.<br><br>2-Year Follow-Up: Both eyes were quiet, with no symblepharon, scar formation, or limbal stem cell deficiency.
Choe et al. 2019[102]	Retrospective interventional case series	SJS: 4 patients (4 eyes)<br><br>Mean Age: 58.5 years	MMG (circumferentially trephined)	BCVA: 3 patients had improvements in BCVA, 1 patient had a decrease in BCVA.<br><br>Limbal Neovascularization: Decreased in 3 patients and increased in one patient.<br><br>Corneal Surface Erosion: Decreased in 3 patients and stayed the same in 1 patient.<br><br>Corneal Opacity: Decreased in 2 patients, stayed the same in 1 patient, and increased in one patient.<br><br>MMG was successful in three patients (60%) over the entire follow-up period. In one patient, central macular edema developed along with a recurrence

				of herpes endotheliitis.
Gopakumar et al. 2019[103]	Retrospective interventional case series	Corneal epithelization<br><br>SJS: 3 patients (4 eyes)<br><br>Mean Age: Corneal Group: 26.3 years<br><br>Fornix reconstruction<br><br>SJS: 8 patients (8 eyes)<br><br>Mean Age Fornix Group: 25.3 years	Autologous cultivated oral mucosal epithelial transplantation (COMET)	Corneal Epithelization:<br><br>Surface epithelized and stable in 3 eyes by day 20.<br><br>Partial epithelization in 1 eye.<br><br>BCVA improved to ≥ 20/200 in all eyes.<br><br>Fornix Reconstructions:<br><br>Successful reconstruction in 6 eyes.<br><br>Partial reconstruction in 2 eyes.<br><br>Forniceal shortening/failure in 1 eye.
Hashimoto et al. 2019[104]	Case report	SJS: 1 patient (2 eyes)<br><br>Age: 40 years	Steroid pulse therapy<br><br>Plasma exchange	At presentation, the patient had severe keratoconjunctivitis with an ocular surface epithelial defect and pseudomembrane formation.<br><br>Despite treatment, ocular complications including blurred vision and dry eye persisted for more than a year.
Iyer et al. 2019[105]	Retrospective interventional case series	SJS: 8 patients (8 eyes)<br><br>Mean Age: 46.7 years	Keratoprosthesis (Boston KPro 2)	BCVA:<br><br>All SJS patients improved in vision to 20/20, except in 1 patient who had no improvement in vision beyond 20/400 due to glaucomatous optic atrophy.<br><br>Retention:<br><br>KPro was retained in 7 out of 8 patients with SJS. One patient required KPro replacement.<br><br>One patient developed endophthalmitis following KPRO surgery.
Kara et al. 2019[106]	Retrospective case series	SJS: 1 patient (2 eyes)<br><br>SJS/TEN: 1 patient (2 eyes)<br><br>TEN: 1 patient (2 eyes)<br><br>Mean Age: 9.3 years	IVIG (all patients)<br><br>Oral clarithromycin (TEN patient)	SJS/TEN patient experienced a persistent corneal defect.<br><br>SJS and TEN patients resolved with no complications.
Lee et al. 2019[107]	Retrospective interventional case series	SJS: 8 patients (8 eyes)<br><br>Mean Age:	Corneoscleral contact lens	Successful lens fitting in 6 patients.<br><br>Lens fitting failed in 2 patients.<br><br>BCVA improved in all patients who were

		35.7 ± 15.4 years		successfully fitted.<br><br>Refractory PED (persistent epithelial defect) in the 2 SJS eyes improved completely.<br><br>CFS scores improved significantly in the 5 SJS eyes with punctate epithelial erosion (PEE).
Shanbhag et al. 2019[108]	Retrospective interventional case series	SJS/TEN: 4 patients (8 eyes)<br><br>Mean Age: 16.8 years	AMT (sutureless technique using a symblepharon ring)	Seven eyes retained BCVA of ≥ 20/40 at the last follow-up.<br><br>One eye had a BCVA of 20/80 secondary to symblepharon extending to the corneal surface and neovascularization.
Shanbhag et al. 2019[109]	Retrospective cohort study	48 patients (96 eyes) total divided into 2 groups<br><br>Group 1 (tx before 2008; 18 eyes)<br><br>SJS: 1 patient<br><br>SJS/TEN: 1 patient<br><br>TEN: 7 patients<br><br>Mean Age Group 1: 34.2 ± 19 years<br><br>Group 2 (tx after 2008; 78 eyes)<br><br>SJS: 11 patients<br><br>SJS/TEN: 9 patients<br><br>TEN: 19 patients<br><br>Mean Age Group 2: 29.1 ± 18.7 years	Topical treatment alone (Group 1)<br><br>Topical treatment ± AMT/ProKera (Group 2)	Presentation: All eyes where BCVA could be measured in Groups 1 and 2 had BCVA ≥ 20/40.<br><br>Follow-Up: A significantly higher proportion of eyes retained BCVA ≥ 20/40 in Group 2 compared to<br><br>Group 1 at last follow-up ($p < 0.001$). A significantly higher proportion of eyes in Group 1 had BCVA < 20/200 as compared to eyes in Group 2 at last follow-up ($p < 0.001$).<br><br>The percentage of eyes with vision-threatening complications in the chronic phase was<br><br>significantly higher in Group 1 (67%, 12/18) versus Group 2 (17%, 13/78, $p = 0.002$).
Wang et al. 2019[110]	Retrospective interventional case series	SJS: 49 patients (94 eyes)<br><br>Mean Age: 6.4 years	PROSE	BCVA Presentation: Median BCVA was 0.6 logMAR (Snellen 20/80, range, 20/20 to HM; n = 83 eyes).<br><br>BCVA Follow-Up: Median BCVA final visit recorded was 0.18 logMAR (Snellen 20/30, range, 20/20<br><br>to HM, n = 61 eyes), which was a significant improvement compared to the initial BCVA (unpaired t test, $P = 0.0004$).<br><br>Fifteen of forty-nine patients (30 eyes; 30.6%) failed PROSE treatment.

				Nine patients who were categorized as fit or training failures and six patients who were categorized as wearing failures.
Xiang et al. 2019[111]	Retrospective cohort study	SJS/TEN: 39 patients divided into two groups<br><br>Irrigation group (n = 21 cases; 42 eyes) received lacrimal passage irrigation with dexamethasone drops twice a week for 4 weeks while in the acute stage<br><br>Mean Age Irrigation Group: 8.1 ± 5.8 years<br><br>Control group (n = 18 cases; 36 eyes) received no irrigation in the acute stage<br><br>Mean Age Control Group: 8.9 ± 6.1 years	Lacrimal system irrigation with dexamethasone drops	Only 2 of 21 (9.52%) patients in the irrigation group were found to have lacrimal system obstructions, compared to 14 of 18 (77.78%) patients in the control group. The rate of lacrimal system obstruction in the chronic stages of SJS or TEN in the irrigation group was much lower than in the control group ($p < 0.01$).<br><br>A significant difference was also found between two groups in the rates of epiphora during the chronic stage ($p = 0.047$).
Yoon et al. 2019[112]	Prospective interventional case series	SJS: 8 patients (8 eyes) with corneal neovascularization<br><br>Mean Age: 61.3 ± 16.7 years	Photodynamic therapy with verteporfin (6 mg/m2) combined with intrastromal bevacizumab injection (2.5 mg/0.1 mL)	Three- and Six-Month Follow-Up: At 3 and 6 months after treatment, all eyes showed regression of corneal neovascularization. Complete regression was achieved in five eyes (62.5%) and partial regression in three eyes (37.5%).<br><br>One-Year Follow-Up (5 Patients): Two eyes maintained complete regression and one eye maintained partial regression. Two eyes with severe chronic ocular manifestation showed revascularization.
Abrol et al. 2020[113]	Prospective case series	Total: 27 patients (54 eyes)<br><br>Mean Age: Not reported	Topical antibiotics, corticosteroids, and artificial tears	Eight patients (16 eyes; 29.6%) developed chronic sequelae of ocular manifestations.<br><br>Severe Dry Eye Disease: 3 patients.<br><br>Trichiasis: 2 patients.<br><br>Diminution of Vision: 2 patients.<br><br>Severe Photophobia: 1 patient.
Alvarado-Villacorta et al.	Case report	SJS: 1 patient (1 eye)	Allogeneic limbal epithelial stem cell transplantation with	At the first week after the procedure, the corneal epithelium had fully epithelized. At month 3, visual

2020[114]		Age: 59 years	solid activated platelet-rich plasma (PRP)	acuity improved from hand motion to 20/70.
De la Sen-Corcuera et al. 2020[115]	Retrospective case series	SJS: 3 patients (5 eyes) with cicatrizing conjunctivitis<br><br>Mean Age: 59.7 ± 16.5 years	Immunosafe plasma rich in growth factors (isPRGF)	Degree of Inflammation:<br><br>At Presentation:<br><br>Two eyes with moderate inflammation (grade of 2).<br><br>One eye with severe inflammation (grade of 3).<br><br>Two eyes with very severe inflammation (grade of 4).<br><br>At End of the Follow-Up:<br><br>All eyes with mild inflammation (grade of 1).<br><br>Statistically significant decrease in inflammation (p = 0.004).<br><br>BCVA:<br><br>Improved in 1 eye (LogMAR 3.0 to LogMAR 0.155).<br><br>Remained the same in 3 eyes.<br><br>Worsened in 1 eye (LogMAR 0.699 to LogMAR 1.0).<br><br>Regarding safety, no adverse effects were reported<br><br>during the study.
Itoi et al. 2020[116]	Prospective interventional case series	SJS: 10 patients (10 eyes)<br><br>Mean Age: 45.5 ± 17.0 years	Limbal-supported contact lens	BCVA:<br><br>Compared to BCVA with spectacle correction at baseline (0.99 log MAR), BCVA after 3 months with limbal CL correction (0.47 log MAR) showed significantly improvement (P = 0.0039), and in 8 eyes (80.0%) the BCVA improvement was more than 0.2 log MAR.<br><br>NEI-VFQ 25:<br><br>In all subjects, the NEI VFQ-25 score improved. However, only in ocular pain and mental health was this improvement statistically significant (P= 0.0078 and 0.0039).<br><br>Adverse Events:<br><br>There were 2 cases of conjunctival epithelial erosion. In those 2 eyes, the small conjunctival epithelial erosion was healed within several days after the removal of the CL. No other complications occurred.
Nakatsuka et al. 2020[117]	Case report	SJS: 1 patient (2 eyes)<br><br>Age: 14 months	AMT (with a modified symblepharon ring) and ProKera	Patient was noted to have some scarring of the palpebral conjunctiva, no symblepharon was noted, and the eyelid margins appeared healthy.
Shanbhag et al.	Retrospective	SJS/TEN: 29 patients (55	AMT: 31 eyes	BCVA:

2020[118]	case series	eyes)<br><br>Median Age: 23 years	ProKera: 24 eyes	At Presentation: 82% of eyes had BCVA ≥ 20/40.<br><br>At Last Follow-Up: 87% of eyes had BCVA ≥ 20/40.<br><br>Forty percent of eyes (22/55) required a repeat AMT or PD.<br><br>Complications:<br><br>Meibomian Gland Disease: 78% (43) of eyes.<br><br>Dry Eye: 58% (32) of eyes.<br><br>Severe Eyelid Margin Keratinization: 31% (17) of eyes.<br><br>Persistent Epithelial Defect: 15% (8) of eyes.<br><br>Sterile Corneal Perforation: 2% (1) of eyes.<br><br>Infectious Keratitis 4% (2) of eyes.
Shanbhag et al. 2020[119]	Retrospective comparative case series	Total: 401 patients (705 eyes)<br><br>81 children (134 eyes)<br>320 adults (571 eyes) divided into two treatment groups<br><br>Conservative Treatment: n = 363<br><br>Definitive Treatment: n = 342<br><br>Mean Age: Not reported	Conservative Treatment: Topical lubricants, steroids, epilation, electrolysis, and punctal cautery<br><br>Definitive Treatment: MMG, PROSE, MMG + PROSE	BCVA:<br><br>Conservative Treatment Group: BCVA decreased significantly over time, dropping from a median of<br><br>20/80 to 20/500 over a 10-year period (363 eyes; P= 0.0017).<br><br>MMG and PROSE: There was improvement in BCVA from baseline with MMG (P= 0.003) and PROSE (P=0.009) over 5 years of follow-up.<br><br>Among children, there was a significantly greater improvement in BCVA with MMG (Figure 4), from a median of 20/100 to 20/30 (19 eyes), compared with BCVA of 20/60 with PROSE (13 eyes; P = 0.01), and BCVA of 20/126 in the control group (82 eyes; P < 0.0001).<br><br>Among adults, PROSE resulted in significantly greater improvement in BCVA, from a median of 20/100 to 20/40 (77 eyes), compared with BCVA of 20/60 with MMG (81 eyes; P = 0.028) and BCVA of 20/160 (281 eyes; P < 0.0001),<br><br>MMG + PROSE: In 20 eyes of children and 132 eyes of adults, BCVA improved from a median of 20/100 to 20/25 (P < 0.0001).<br><br>At 5 years of follow-up (Figure 4), children who received MMG + PROSE had significantly better BCVA than those receiving MMG alone (P = 0.036), PROSE alone (P = 0.0002), or conservative therapy (P < .0001). Similarly, adults who received MMG + PROSE had significantly better BCVA than those receiving MMG alone (P = 0.012), PROSE alone (P = 0.0043), or conservative therapy (P < .0001).<br><br>Risk of Complications (in the first year after presentation):<br><br>Conservative Therapy: 18.3% (15/82) in children and 23.8% (67/281) in adults.<br><br>PROSE Alone: 7.7% (1/13) in children (P = 0.799)

				and 9.1% (7/77) in adults (P = 0.0086).<br><br>MMG Alone: 5.3% (1/19) in children (P = 0.161) and 11.1% (9/81) in adults (P = 0.023).<br><br>PROSE + MMG: 5% (1/20) in children (P = 0.14) and 5.3% (7/132) in adults (P < .0001).<br><br>Those who received conservative therapy had a 2.61 (82/363 vs. 26/342) times greater risk and 3.54 times (82/281 vs. 26/316) greater odds of developing corneal ulceration and/or perforation within the first year compared with those who received definitive therapy.
Shegaonkar 2020[120]	Case report	TEN: 1 patient (2 eyes)<br><br>Age: 70 years	Topical antibiotics and lubricants<br><br>Systemic antibiotics and steroid	Within a few hours, despite topical and systemic therapies and planned AMT, both eyes developed pan-ophthalmitis with restricted extraocular movements and mild proptosis and had to be eviscerated.<br><br>The patient died after 48 h of evisceration due to multi-organ failure secondary to TEN.
Shimazaki et al. 2020[121]	Retrospective case series	SJS: 35 patients (51 eyes)<br><br>Mean Age: 48.7 ± 17.6	Limbal stem cell transplantation<br><br>Auto-CLET n = 4<br>Allo-CLET n = 27<br>Lr-CLET n = 4<br>COMET n = 42	Successful Ocular Surface Reconstruction: 54.9% of eyes at last follow-up.<br><br>Clear Cornea: 64.7% of eyes at last follow-up.<br><br>BCVA ≥20/200: 16.1% of eyes at last follow-up.
Sudana et al. 2020[122]	Case report	SJS: 1 patient (1 eyes)<br><br>Age: 47 years	MMG + scleral lens (PROSE)	BCVA improved to 20/25 with scleral lens in the left eye, which was sustained over 1 year of follow-up.
Chan et al. 2021[123]	Case report	SJS: 1 patient (2 eyes)<br><br>Age: 15 years	AMT (with fibrin glue)	BCVA:<br><br>At Presentation: 20/30 unaided in each eye.<br><br>At 8-Week Follow-Up: 20/20 unaided bilaterally.<br><br>After the second round of treatment, the corneal epithelium was healed and did not show any symblephara.<br><br>At follow-up, the fornices were clear, with no pyogenic granuloma and only a minimal nasal symblepharon present in the left eye. The corneas were clear, with no sign of limbal stem cell failure or dry eye symptoms.
Elhusseiny et al. 2021[124]	Case report	SJS/TEN: 1 patient (2 eyes)<br><br>Age: 2 months	AMT<br><br>IVIG<br><br>Topical ABX and lubricants	Two-Week Follow-Up: The entire ocular surface had healed, with no signs of conjunctival and/or corneal inflammation or ulceration.<br><br>Two-Month Follow-Up: The ocular examination showed normal anterior segment with no evidence of lid margin keratinization, entropion, trichiasis, or any other complication.
Hall et al. 2021[125]	Retrospective comparative cohort study	Total: 14 patients (28 eyes) divided into two	Systemic cyclosporine (CsA)	BCVA, chronic ocular surface complication score (COCS), meibomian gland dysfunction, limbal stem cell deficiency, and the need for mucous membrane grafting and scleral lenses were not significantly different between patients who received systemic

		groups<br><br>Patients who received CsA: n = 7<br><br>Patients who did not receive CsA: n = 7<br><br>Median Age: 29 years		CsA and patients who did not receive systemic CsA.
Huhtanen et al. 2021[126]	Case report	SJS: 1 patient (2 eyes)<br><br>Age: 29 years	Mini scleral contact lenses	BCVA:<br><br>At Presentation: Spectacle refraction OD −0.25 DS, OS −0.75 DS gave visual acuities of both right eye and left eye 6/6−1 in both eyes.<br><br>At Follow-Up: With the lenses, the patient achieved visual acuities of 6/6 in each eye.<br><br>Slit lamp examination at follow-up revealed a marked improvement in the integrity of the ocular surface.
Jabbour et al. 2021[127]	Retrospective case series	SJS: 91 patients (182 eyes)<br><br>Mean Age: 25.4 years	Contact lens wear (RGP or SCL): 49.5% (46 patients; 92 eyes)<br><br>Mucous membrane grafting: 10.0% (10 patients; 20 eyes)	BCVA:<br><br>At Presentation: Majority of patients had decimal vision worse than 0.33 (6/18 or worse).<br><br>At Follow-Up:<br><br>Contact Lenses: Final decimal visual acuity was 0.21 ± 0.10 (0.00 to 1.20), compared to 0.01 ± 0.00 (0.00 to 1.20) with those with no RGP or scleral lenses (p= 0.001).<br><br>MMG: Final decimal visual acuity was 0.31 ± 0.58 (0.00 to 1.50), compared to 0.56 ± 0.57 (0.01 to 1.20) at first visit (p= 0.236).<br><br>Authors found that, in the cohort, visual acuity at presentation was an important prognostic sign for long-term visual acuity, regardless of management and clinical course.<br><br>* A variety of SJS treatments were provided to this cohort of patients, but not all of them were commented on in the text by the authors. Only treatments whose outcomes were reported by the authors were included. *
Jovanovic et al. 2021[128]	Retrospective cohort study	SJS: 38 eyes divided into two groups<br><br>5-Fluorouracil: 9 eyes<br><br>No 5-Fluorouracil: 28 eyes<br><br>Age Ranges:<br><br>8–35: 7 patients (14	5-Fluorouracil (serial injections into fornices)	The 5-FU injection treatment variable was associated with reported overall improvement, decreased number of MMG/AMG surgeries, less corneal scarring on the last encounter, and improved trichiasis/districhiasis on the last encounter.<br><br>The total number of 5-FU injections showed association with reported overall improvement and number of MMG/AMG surgeries.

		eyes)<br><br>36–65: 25 patients (50 eyes)<br><br>≥65: 24 patients (48 eyes)		
Ma et al. 2021[129]	Retrospective cohort study	Total: 119 patients (236 eyes)<br><br>SJS: 87<br><br>SJS/TEN: 9<br><br>TEN: 23<br><br>Mean Age: 45.6 ± 22.7 years	Systemic etanercept + corticosteroids<br><br>AMT	Sotozono's Grading Score:<br><br>Etanercept:<br><br>Acute Stage: The average acute-stage score for patients who received additional systemic etanercept (1.32 ± 0.76 (0–3)) was not significantly different from that of patients who only received systemic steroids (1.49 ± 0.90 (0–3; p= 0.171)).<br><br>Chronic Stage: The average score for additional etanercept-treated patients was 1.64 ± 2.47 (0–14), which was significantly lower than the score for corticosteroid-only-treated patients (3.95 ± 5.76; range 0–33, p < 0.001).<br><br>AMT:<br><br>Chronic Stage: Patients receiving earlier AMT (< 7 days) were associated with less severe chronic ocular complications.
Ma et al. 2021[130]	Case series	Total: 5 patients (10 eyes)<br><br>SJS: 4 patients<br><br>TEN: 1 patient<br><br>Mean Age: 53.4 ± 9.3 years	Topical steroids, ABX, and lubricants (5 patients)<br><br>AMT (2 patients)	Patient 1 (AMT + Topical Treatment):<br><br>At Presentation: 20/400 OD, 20/200 OS.<br><br>At Follow-Up: Not reported.<br><br>Complications: Dry eye but no superficial punctate keratopathy, OU trichiasis (+), tarsal scarring.<br><br>Patient 2 (AMT + Topical Treatment):<br><br>At Presentation: 20/100 OD, 20/400 OS.<br><br>At Follow-Up: 20/40 OD, 20/400 OS.<br><br>Complications: Dry eye with fine superficial punctate keratopathy, OU tarsal scarring.<br><br>Complete corneal and conjunctival re-epithelialization was achieved after AMT.<br><br>Patient 3 (Topical Treatment):<br><br>BCVA not reported, likely lost to follow-up.<br><br>Patient 4 (Topical Treatment):<br><br>BCVA not reported.<br><br>Clear cornea OU.<br><br>Patient 5:<br><br>At Presentation: 20/40 OD, 20/25 OS.

				At Follow-Up: Not reported.<br><br>Clear cornea OU.
Mahmood et al. 2021[131]	Case report	SJS/TEN: 1 patient (2 eyes)<br><br>Age: 24 years	Systemic steroids<br><br>Systemic cyclosporine A<br><br>Subcutaneous (SC) etanercept<br><br>Topical steroids and lubricants<br><br>IVIG<br><br>Bandage contact lenses<br><br>ProKera	After extensive multimodal treatment, the patient's symptoms further improved, and on her last visit to the clinic, she achieved visual acuity of 6/9 in both eyes.<br><br>Examination showed quiet eyes and a wet ocular surface with only a few superficial punctate keratopathy. No new symblepharon or ankyloblepharon formation was noted. The lid margin anatomy and irregularity improved significantly.<br><br>Despite treatment, the patient did have signs of meibomian gland dysfunction and keratinization. The patient also still had symptoms of dry eyes that were managed sufficiently with lubricating eye drops and ointments.
Maqsood et al. 2021[132]	Cohort study	SJS: 10 patients (10 eyes)<br><br>Mean Age: 54.8 ± 24.2 years	AMT (Omnigen)	Persistent Epithelial Defect:<br><br>Completely Healed: 2 patients (20.0%).<br><br>Partially Healed: 5 (50.0%).<br><br>Defects with No Improvement: 3 (30.0%).
Mieno et al. 2021[133]	Retrospective, comparative case series.	Total: 85 patients (170 eyes) divided into two groups<br><br>Group A (Patients who Received CPT within 4 Days after Disease Onset): n = 36<br><br>Mean Age Group A: 32 years<br><br>Group B (Patients Who Did Not Receive CPT within 4 Days after Disease Onset): n = 49<br><br>Mean Age Group B: 35 years	Corticosteroid pulse therapy (CPT)	BCVA in the worse eye (decimal) < 0.001.<br><br>≥ 1.0: Group A = 19 (52.8%), Group B = 7 (14.3%).<br><br>0.1-0.9: Group A = 8 (22.2%), Group B = 19 (38.8%).<br><br>< 0.1: Group A = 9 (25.0%), Group B = 23 (46.9%).<br><br>Visual prognosis was better in group A than in group B; p < 0.001.<br><br>Complications: The number of severe ocular sequelae (a worsening of BCVA and corneal and conjunctival complications) was significantly less in group A than in group B.<br><br>Regardless of the treatment administered at the acute stage, dry eye was highly prevalent at the chronic stage of the disease.
Mimouni et al. 2021[134]	Retrospective case series	SJS: 2 patients (2 eyes)	AMT + bandage contact lenses	Patient 1:<br><br>BCVA at Presentation: 20/40.

		Mean Age: 57.5 years		BCVA at Follow-Up: 20/30.<br><br>Time to Epithelialization of PED: 14 days.<br><br>Patient 2:<br><br>BCVA at Presentation: 20/50.<br><br>BCVA at Follow-Up: 20/50.<br><br>Time to Epithelialization of PED: 35 days.
Moon et al. 2021[135]	Prospective interventional case series	SJS: 2 patient (4 eyes)<br><br>Mean Age: 33 years	Scleral lens (LK)	Outcomes for only 1 SJS patient were explicitly reported.<br><br>Patient 1:<br><br>BCVA logMAR:<br><br>At Presentation: 0.82 OD, 0.52 OS.<br><br>At Follow-Up: 0.15 OD, OS not reported.<br><br>The corneal fluorescein staining score did not show<br><br>any significant difference from baseline.
Ngowyutagon et al. 2021[136]	Case report	SJS: 1 patient (1 eye) with complete ankyloblepharon<br><br>Age: 55 years	Stage 1: AMT + MMG<br><br>Stage 2: Simple oral mucosal epithelial transplantation (SOMET)	BCVA<br><br>At Presentation: Light perception OD.<br><br>At Follow-Up: Counting fingers (1 foot) OD.<br><br>Complete epithelialization of cornea-like epithelium was observed within 6 weeks after SOMET was performed.
Pushker et al. 2021[137]	Randomized control study	SJS: 20 patients (40 eyes) divided into two groups<br><br>Fibrin glue MMG (n = 10)<br><br>Polygalactin suture MMG (n = 10)<br><br>Mean Age: 20.5 ± 3.7 years	MMG (two techniques)	The BCVA showed significant improvement from the preoperative period to the postoperative 6 months in both of the study groups. The median BCVA in the FG and PS groups, in logMAR units, was 0.385 (0–2.48, range) and 1.78 (0–2.48, range) at baseline (p= 0.06), respectively, and 0.30 (0–2.48) and 0.39 (0–2.48) at 6 months (p= 0.05), respectively.<br><br>The dry eye parameters (TBUT and Schirmer-1 test scores) showed statistically significant improvement following MMG in both of the study groups, with no significant intergroup difference.<br><br>Graft displacement:<br><br>Fibrin Glue MMG: NA.<br><br>Polygalactin Suture MMG: 1 eyelid.<br><br>As MMG with sutures involves significantly longer intraoperative time and delay in corneal surface healing, fibrin glue is a viable option for the apposition of mucous membrane grafts in the correction of lid margin pathologies with similar clinical outcomes.
Sant'Anna et al.	Prospective interventional	SJS: 11 patients (11 eyes) with	Minor salivary gland transplantation +	All patients showed improvement in eyelid positioning and dry eye signs and symptoms after

2021[138]	case series	severe entropion<br><br>Mean Age: 32 years	MMG	surgery.
Santamaria et al. 2021[139]	Case report	SJS: 1 patient (2 eyes)<br><br>Age: 18 years	Surgical debridement<br><br>AMT<br><br>Bandage contact lens (Kontour)<br><br>Topical treatment (steroids, ABX, lubricants)	At presentation, patient had complete fusion of the bilateral upper and lower eyelids, precluding visualization of the cornea or sclera.<br><br>BCVA:<br><br>At Presentation: Light perception OU.<br><br>At Follow-Up: 20/100 OU.<br><br>Significant dry eye persisted despite treatment.
Shahraki et al. 2021[140]	Case report	SJS: 1 patient (2 eyes)<br><br>Age: 45 years	IV steroids<br><br>IV ABX<br><br>Topical treatment (steroid, ABX, lubricant, autologous serum)	BCVA:<br><br>At Presentation: Counting fingers OU.<br><br>At Follow-Up: 20/30 OD and 20/20 OS.<br><br>Complications: A scar formed in the superotemporal area of the cornea OD. Meibomian glands were severely disrupted, and an irregular eyelid margin stained with fluorescein was observed in both eyes.
Utine et al. 2021[141]	Case series	TEN: 1 patient (2 eyes) with severe corneal melt following penetrating keratoplasty (PK)<br><br>Age: 38 years	TNF-alpha inhibitor (IV infliximab infusion)<br><br>Prior Treatment: PK, AMT, punctal cautery	Six months after initiation of infliximab therapy, ocular surface was healed, with complete epithelialization and corneal neovascularization, and with no signs of melting.
Venugopal et al. 2021[142]	Prospective interventional case series	SJS: 41 patients (45 eyes)<br><br>Mean Age: 25.4 ± 11.6 years	Cultivated oral mucosal epithelial transplantation (COMET; fornix reconstruction)	Mean Corrected-Distance Visual Acuity (CDVA):<br><br>At Presentation: 2.68 ± 0.47 logMAR.<br><br>At Follow-Up (12 months): 1.54 ± 0.74 logMAR.<br><br>At Follow-Up (24 months): 1.49 ± 0.98 logMAR.<br><br>There was a significant improvement in mean CDVA at follow-up compared to presentation.<br><br>Overall, 82.2% of eyes (37/45) had improvement in visual acuity, and 13.3% (6/45) experienced no change, whereas 2 eyes (4.4%) had worsening of visual acuity.<br><br>Mean Ocular Surface Severity Score:<br><br>At Presentation: 29.1 ± 9.7.<br><br>At Follow-Up (2 years): 18.7 ± 7.2.<br><br>Two eyes developed corneal persistent epithelial defects, progressing to corneal melting in one eye and microbial keratitis in the second one, at postoperative week 2.

Yang et al. 2021[143]	Retrospective case series	Total: 16 patients (32 eyes)<br><br>Mean Age: 27.2 ± 21.5 years	AMT	BCVA:<br><br>At Presentation (available in 16 eyes):<br><br>BCVA ≥20/40 = 4/16 eyes.<br><br>BCVA b/w 20/40 & 20/200 = 7/16 eyes.<br><br>BCVA ≤20/400 = 5/16 eyes.<br><br>At Follow-Up (Final):<br><br>BCVA ≥20/40 = 21/32 (65%) eyes.<br><br>BCVA b/w 20/40 & 20/200 = 7 (22%) eyes.<br><br>BCVA ≤20/400 = 4 (12%) eyes.<br><br>The four eyes that had BCVA ≤20/400 at final follow-up all had delayed AMT.<br><br>Complications: Trichiasis (12/32), lid margin keratinization (28/32), lid entropion (7/32), LSCD (8/32),<br><br>distichiasis (9/32), dry eye (26/32), tarsal conjunctival scarring (18/32), symblepharon (18/32), ankyloblepharon (2/32).<br><br>A significant proportion of chronic complications occurred after 6 months of AMT.
Aziza et al. 2022[144]	Case series	SJS: 3 patients (5 eyes)<br><br>Mean Age: 58.3 years	Patient 1: Ankyloblepharon lysis + oral steroids + topical treatment (steroids, ABX, lubricants)<br><br>Patient 2: Symblepharon separation + mitomycin C+ AMT+ IV steroids + oral steroids + oral Immunosuppressant + topical treatment (steroids, ABX, lubricants) + limbal rigid contact lenses<br><br>Patient 3:<br><br>Symblepharon lysis + mitomycin C +AMT + keratolimbal allograft + COMET + IV steroids + oral steroid + oral immunosuppressant	Patient 1:<br><br>BCVA:<br><br>At Presentation: 2.8 logMAR OU.<br><br>At Follow-Up: 0.7 OD and 0.4 OS logMAR.<br><br>Patient 2:<br><br>BCVA:<br><br>At Presentation: Light perception OU.<br><br>At Follow-Up: 0.5 OD and 1.2 OS logMAR (with contact lenses).<br><br>Complete epithelialization at 2 months.<br><br>Patient 3:<br><br>BCVA:<br><br>At Presentation: 2.8 OD logMAR.<br><br>At Follow-Up: 1.1 logMAR (with contact lenses).<br><br>Complete epithelialization at 3 months post-COMET.
Booranapong et al.	Case series	SJS: 3 patients (6	COMET	Patient 1:

2022[145]		eyes)<br><br>Mean Age: 46.2 years		BCVA:<br><br>At Presentation: Hand motion OU.<br><br>At Follow-Up (1 year): Count fingers (1 foot) OU.<br><br>Overall Grade of Surgical Outcomes: Good.<br><br>Patient 2:<br><br>BCVA:<br><br>At Presentation: Count fingers (1 foot) OU.<br><br>At Follow-Up (1 year): Count fingers (1 foot) OU.<br><br>Overall Grade of Surgical Outcomes: Poor.<br><br>Patient 3:<br><br>BCVA:<br><br>At Presentation: Light perception OU.<br><br>At Follow-Up (1 year): Hand motion OU.<br><br>Overall Grade of Surgical Outcomes: Poor.<br><br>Two patients with SJS had corneal ulcers at 2 weeks postoperatively.
Iannetti et al. 2022[146]	Retrospective case series	SJS/TEN: 7 eyes<br><br>Mean Age: 31.0 ± 7.7 years	Keratoprosthesis	BCVA:<br><br>At Presentation: 2.69 ± 0.30 logMAR.<br><br>At Follow-Up (60 months): 0.11 ± 0.21 logMAR (7 eyes).<br><br>At Follow-Up (360 months): 0.11 ± 0.11 logMAR (3 eyes).
Kanazawa et al. 2022[147]	Case report	SJS: 1 patient (2 eyes)<br><br>Age: 41 years	IVIG<br><br>IV steroids	Patient recovered after treatment but had chronic ocular complications. Notably, corneal opacification caused visual impairment.
Katz et al. 2022[148]	Retrospective case series	SJS: 1 patient (2 eyes)<br><br>Age: 60 years	Topical treatment (Triple Play: steroids, pooled human immune globulin, and serum tears)	BCVA:<br><br>Baseline: Hand motion OD, 20/300 OS.<br><br>Post-Treatment: 20/125 OD, 20/100 OS.<br><br>Ocular Discomfort Score:<br><br>Baseline: 10 OU.<br><br>Post-Treatment: 2 OU.<br><br>Ocular Surface Disease Index:<br><br>Baseline: 90.<br><br>Post-Treatment: 35.

				Conjunctival Redness:<br>Baseline: 50 OU.<br>Post-Treatment: 40 OU.<br>Subjective Global Assessment Post-Treatment: Improved OU.<br>Over two months, epithelial defects healed.
Komai et al. 2022[149]	Retrospective case series	SJS: 1 patient (2 eyes)<br>Age: 52 years	Cultivated oral mucosal epithelial transplantation (COMET; fornix reconstruction)	Fornix reconstruction with COMET was successful (patient followed for 7 years post-COMET).
Liao et al. 2022[150]	Retrospective case series	SJS: 1 patient (2 eyes)<br>TEN: 1 patient (2 eyes)<br>Mean Age: 22.5 years	PROSE (scleral lenses)	Patient 1:<br>BCVA:<br>At Presentation: 20/20 OD, 20/30^{-1}.<br>At Follow-Up (8 months): 20/20^{+2} OD, 20/15^{-3} OS.<br>Patient 2:<br>BCVA:<br>At Presentation: 20/50^{+1} OD, 20/100^{-1} OS.<br>At Follow-Up: 20/30^{-2} OD, 20/60 OS.<br>Regression of neovascularization and clearing of corneal<br>opacification occurred in both patients.
Mitani et al. 2022[151]	Case report	SJS: 1 patient (2 eyes)<br>Age: 15 years	IV steroids (pulse therapy)<br>Topical treatment (steroid, ABX)	Ocular lesions improved without serious visual impairment.
Pradeep et al. 2022[152]	Retrospective case series	SJS/TEN: 5 patients (10 eyes)<br>Mean Age: 27.5 years	AMT	BCVA:<br>At Presentation: Not reported.<br>At Follow-Up (1 year): 9 eyes achieved a BCVA of ≥ 6/12; 1 eye had a BCVA of 6/36.<br>Complications (at 1-year follow-up):<br>3/5 patients had lid margin keratinization;<br>0/5 patients had symblepharon;<br>0/5 patients had ocular surface keratinization;<br>4/5 patients had dry eye;<br>1/5 patients had corneal vascularization.<br>* Only treatments whose outcomes were explicitly discussed in the text were included in the table*.

Ryu et al. 2022[153]	Case report	SJS: 1 patient (2 eyes)<br><br>Age: 64 years	IV steroids<br><br>Oral steroids<br><br>Topical treatment (steroid, ABX, lubricants)<br><br>Punctal occlusion	BCVA:<br><br>At Presentation: Counting fingers OU.<br><br>At Follow-Up: 20/30 OD, 20/50 OS.<br><br>Complications:<br><br>Punctate epithelial erosions (minimal);<br><br>Meibomian gland dysfunction;<br><br>Decreased tear meniscus height;<br><br>Mild eyelid keratinization.
Sharma et al. 2022[154]	Case report	SJS: 1 patient (2 eyes)<br><br>Age: 30 years	AMT + MMG + electroepilation	BCVA:<br><br>At Presentation: 20/25 OU.<br><br>At Follow-Up: 20/20 OU.<br><br>In the first year after AMT, the patient developed eyelid-related complications such as trichiasis, distichiasis, eyelid margin keratinization (LMK), and cicatricial entropion. Consequently, she underwent MMG and electroepilation in all 4 eyelids.
Sims et al. 2022[155]	Case report	SJS: 1 patient (2 eyes)<br><br>Age: 20 years	AMT (sutureless)	BCVA:<br><br>At Presentation: 20/20 OU.<br><br>At Follow-Up: 20/20 OU.<br><br>Outpatient follow-up demonstrated preserved vision without corneal scarring or ulceration.<br><br>Complications:<br><br>Inferior forniceal banding of the left eye;<br><br>Obliterated meibomian glands;<br><br>Dry eye (managed well with scleral lenses).
Susiyanti et al. 2022[156]	Prospective interventional case series	SJS: 5 patients (5 eyes)<br><br>Median Age: 46 years (22–71)	Umbilical cord serum eye drops	After four weeks of treatment, ocular surface disease index (OSDI) score, Schirmer I, and keratoepitheliopathy scores improved significantly, while Non-Invasive Tear Breakup Time (NIBUT) scores improved insignificantly.<br><br>Three of five eyes treated with UCS drops had complete corneal reepithelization after 4 weeks.<br><br>No adverse effects were reported.
Arboleda et al. 2023[157]	Case report	SJS: 1 patient (1 eye)<br><br>Age: 67 years	Staged treatment approach<br><br>1. Systemic immunosuppressant (methotrexate)<br><br>2. Minor salivary gland transplant	BCVA :<br><br>At Presentation: No light perception OD.<br><br>At Follow-Up (24 months post-KPro): 20/60 OD.

			(MSGT)<br><br>3. Lid margin mucous membrane graft (MMG)<br><br>4. Keratoprosthesis with AMT (Boston Type 1)	
Asghari et al. 2023[158]	Case series	SJS: 1 patient (1 eye)<br><br>Age: 35 years	PROSE (scleral lens)	BCVA:<br><br>At Presentation: 20/100 OD.<br><br>At Follow-Up: 20/20 OD.<br><br>No adverse effects were reported.
Bourke et al. 2023[159]	Case report	SJS: 1 patient (2 eyes)<br><br>Age: 15 years	IV steroids<br><br>Oral steroids<br><br>IV infliximab<br><br>Oral immunosuppressant<br><br>AMT<br><br>Autologous serum eye drops	BCVA:<br><br>At Presentation: 6/15 OD, 6/9 unaided (6/6 with pinhole) OS.<br><br>At Follow-Up: 6/12 OD, 6/9 OS.<br><br>Complete epithelialization of both ocular surfaces<br><br>occurred within 1 week of AMT.<br><br>Complications:<br><br>Trichiasis and distichiasis OU;<br><br>Severe forniceal shortening OU.
Ceylan et al. 2023[160]	Retrospective interventional case series	Total: 13 patients (26 eyes)<br><br>TEN: 9 patients (18 eyes)<br><br>SJS: 4 patients (8 eyes)<br><br>Mean Age: 38.4 ± 17.6 years	AMT (sutureless)	AMT was performed once for 8 patients and twice for 5 patients.<br><br>Ocular Findings:<br><br>At Presentation:<br><br>Lid margin inflammation = 22 (84.6%);<br><br>Early symblepharon = 6 (23.1%);<br><br>Conjunctival ulceration and inflammation = 26 (100%);<br><br>Pseudomembranous conjunctivitis = 15 (57.6%);<br><br>Corneal epithelial defect = 20 (76.9%);<br><br>Conjunctival scarring = 0;<br><br>Meibomian gland dysfunction = 0;<br><br>Eyelid margin keratinization = 0;<br><br>Trichiasis = 0/<br><br>At Last Follow-Up:<br><br>Lid margin inflammation = 0;

				Early symblepharon = 0;<br><br>Conjunctival ulceration and inflammation = 0;<br><br>Pseudomembranous conjunctivitis = 0;<br><br>Corneal epithelial defect = 0;<br><br>Conjunctival scarring = 7 (26.9%);<br><br>Meibomian gland dysfunction = 11 (42.3%);<br><br>Eyelid margin keratinization = 5 (19.2%);<br><br>Trichiasis = 1 (3.8%).
Chiu et al. 2023[161]	Retrospective case series	SJS: 3 patients (6 eyes)<br><br>Mean Age: 70.3 years	ProKera (AMT)<br><br>Topical treatment (lubricants, steroids)<br><br>Symblepharon<br><br>Release (patients 1 and 2)	Patient 1:<br><br>BCVA:<br><br>At Presentation: OD 6/30, OS 6/15.<br><br>At Follow-Up (10 months): 6/15 OU.<br><br>Symblepharon and inflammation resolved.<br><br>Complications: Meibomian gland dysfunction.<br><br>Patient 2:<br><br>BCVA:<br><br>At Presentation: 6/12 OD, counting fingers OS.<br><br>At Follow-Up (7 months): 6/6 OD, 3/60 OS.<br><br>Symblepharon and inflammation resolved.<br><br>Complications: Meibomian gland dysfunction OU,<br><br>corneal conjunctivalization with neovascular formation OS.<br><br>Patient 3:<br><br>BCVA:<br><br>At Presentation: 6/7.5 OD, 6/10 OS.<br><br>At Follow-Up (6 months): 6/6 OU.<br><br>Complete remission of symptoms and signs of eyelid/conjunctiva/cornea.
Doctor et al. 2023[162]	Case report	SJS: 1 patient (1 eyes)<br><br>Age: 18 years	Simple oral mucosal epithelial<br><br>transplantation (SOMET) + MMG + PROSE	BCVA:<br><br>At Presentation: Light perception OS.<br><br>At Follow-Up (1 year): 20/250 OS improving to 20/100 with PROSE contact lenses.<br><br>Cornea completely epithelized by 3 weeks post-MMG + SOMET.
Gupta et al. 2023[163]	Retrospective interventional	SJS: 65 (135 eyes)	Punctal occlusion (30 eyes)	BCVA (1 year follow-up):

	case series	Median Age: 29 years	Epilation/electrolysis (26 eyes)<br><br>Entropion correction<br><br>surgery (5 eyes)<br><br>Mucous membrane<br><br>grafting (44 eyes)<br><br>Topical lubricants (120 eyes)<br><br>Tissue adhesives (12 eyes)<br><br>Tarsorrhaphy (20 eyes)<br><br>AMT (40 eyes)	Improvement: 19 eyes (14.62%).<br><br>BCVA Maintained: 99 eyes (76.15%).<br><br>Worsened: 12 eyes (9.23%) (all 12 correspond to the eyes<br><br>that underwent tissue adhesive application for corneal perforation).
Hooshmandi et al. 2023[164]	Case report	SJS: 1 patient (2 eyes)<br><br>Age: 13 years	AMT OS<br><br>Scleral lenses OU	BCVA:<br><br>At Presentation: Counting fingers OU.<br><br>At Follow-Up (6 months): 20/40 OU with scleral lenses.<br><br>At follow-up, there was no sign of orbital cyst recurrence or symblepharon formation. The inferior fornix had acceptable depth and normal ocular movements.
Matsumoto et al. 2023[165]	Retrospective case series	Total: 10 patients (20 eyes)<br><br>SJS: 9 patients (18 eyes)<br><br>TEN: 1 patient (2 eyes)<br><br>Mean Age: 36.0 ± 23.7 years	Systemic steroids (Oral/IV) + topical steroids	At final follow-up, 16 eyes of 8 patients had a logMAR visual acuity of ≤ 0; LogMAR VA of > 0 was observed in 4 eyes of 2 patients.<br><br>No serious ocular sequelae were observed.<br><br>In SJS/TEN, ocular surface inflammation continues even after systemic inflammation has improved following steroid pulse therapy. Administration of high-dose topical steroids in addition to systemic steroids is key to preventing vision loss in SJS/TEN patients with ocular involvement in the acute stage.
Mimouni et al. 2023[166]	Retrospective interventional case series	SJS: 4 patients (4 eyes)<br><br>Mean Age: 48.3 years	Keratolimbal allograft (KLAL)	Three SJS patients had a successful KLAL. The ocular surface was stable, with a limbal stem cell deficiency (LSCD) grade of 0. All 3 patients had keratinized lid margins requiring the use of bandage contact lenses.<br><br>One SJS patient had a failed KLAL and developed a fungal keratitis with subsequent corneal melting, requiring a tectonic PKP and keratoprosthesis (Boston type 1).<br><br>BCVA:<br><br>Patient 1:<br><br>At Presentation: Counting fingers.

				At Follow-Up: 20/60.<br><br>Patient 2:<br><br>At Presentation: Hand movement.<br><br>At Follow-Up: 20/200.<br><br>Patient 3:<br><br>At Presentation: 20/300.<br><br>At Follow-Up: 20/30.<br><br>Patient 4:<br><br>At Presentation: Hand movement.<br><br>At Follow-Up: 20/400.
Mortensen et al. 2023[167]	Retrospective case series	SJS/TEN: 43 patients (85 eyes)<br><br>Mean Age: 38 years	AMT (two techniques: cryopreserved AMT and dehydrated AMT)	Mean BCVA<br><br>At Presentation: 20/92 (with 21% of eyes worse than 20/200 BCVA).<br><br>At Last Follow-Up: 20/33 (with 32% of eyes achieving 20/20 BCVA and 72% achieving 20/40 or better).<br><br>Complications:<br><br>Punctate Epithelial Erosions: 65% of eyes.<br><br>Lid Margin Keratinization: 54% of eyes.<br><br>Symblepharon formation, madarosis, and meibomian gland dysfunction were significantly more common in the cryopreserved AMT group.
Ravindra et al. 2023[168]	Comparative interventional study	SJS: 44 eyes divided into two groups<br><br>Group 1 (Retinol Palmitate): n = 11 patients (22 eyes)<br><br>Median Age Group 1: 25 years<br><br>Group 2 (Conventional Treatment): n = 12 patients (22 eyes)<br><br>Median Age Group 2: 24.5 years	Topical retinol palmitate	Median BCVA:<br><br>Group 1:<br><br>At Presentation: 2 logMAR.<br><br>At Follow-Up: 1.778 logMAR.<br><br>Group 2:<br><br>At Presentation: 1.778 logMAR.<br><br>At Follow-Up: 1.778 logMAR.<br><br>There was no statistically significant difference between the groups' visual acuity two-sample t-test (p =0.68).<br><br>A statistically significant improvement was seen in patients who received topical retinol palmitate at 12 weeks for the following outcome measures: Schirmer-1 test (p=<0.01), tear prism height on ASOCT (p =0.02), Rose Bengal staining score of cornea (p =0.01) and conjunctiva (p < 0.01), reduction in ocular surface keratinization on impression cytology (p =0.01), and subjective evaluation using OSDI questionnaire (p =0.04).

Shamloul et al. 2023[169]	Case report	SJS: 1 patient (2 eyes)<br><br>Age: 60 years	IV steroids, topical treatment (ABX)	Follow-up one week after discharge, the patient's eyes were almost fully healed, and her vision remained unaffected.
Shlager et al. 2023[170]	Case report	SJS: 1 patient (1 eyes)<br><br>Age: 51 years	PROSE + ophthalmic vitamin A ointment	BCVA:<br><br>At Presentation: 20/25.<br><br>At Follow-Up: $20/20^{-2}$.<br><br>Following re-initiation of vitamin A ointment, there was no progressive corneal keratinization or worsened neovascularization (4-year follow-up).
Shree et al. 2023[171]	Retrospective interventional case series	SJS: 22 patients (22 eyes)<br><br>Mean Age: 41.9 years	MMG	BCVA:<br><br>Preoperative (logMAR) = 1.29 ± 0.95.<br><br>Postoperative (logMAR) = 0.99 ± 0.88.<br><br>Postoperative visual acuity improvement was noted in 50% of the eyes (n = 13), no improvement in 39% of the eyes (n = 10), and vision worsened in 12% of the eyes (n = 3).<br><br>Subjective Assessment of Symptoms:<br><br>Sixteen patients had a subjective improvement in symptoms;<br><br>Five patients had no improvement in symptoms;<br><br>One patient had worsening of symptoms.<br><br>Objective Assessment of Entropion:<br><br>Sixteen patients had corrected entropion;<br><br>Six patients had a recurrent entropion.
Singh et al. 2023[172]	Prospective interventional case series	SJS: 7 patients (8 eyes)<br><br>Mean Age: 33.9 years	5-Fluorouracil (local injection)	All 8 eyes demonstrated a visible reduction in the conjunctival congestion and scarring over the lobar area. The mean OSDI scoring improved from 65.3 to 51.1.<br><br>None of the patients developed any corneal epithelial defects or worsening of dry eye symptoms during the follow-up period after receiving injection.<br><br>5-FU can be tried in SJS patients without any ocular surface side effects. It improves the degree of periglandular congestion; however, it does not improve the tear secretion significantly, especially in patients with zero Schirmer values.
Singh et al. 2023[173]	Prospective interventional case series	SJS: 6 eyes<br><br>Mean Age; 37.8 ± 14 years	MMG + anterior<br><br>lamellar recession (ALR)	MMG was successful in all 6 eyes of SJS patients.<br><br>All patients reported subjective improvement in symptoms.
Suzuki et al. 2023[174]	Case report	SJS: 1 patient (2 eyes)<br><br>Age: 45 years	IVIG<br><br>Topical steroids	The patient had a recurrence of pseudomembranes, and symblepharon was observed on day 6 after hospitalization.<br><br>Eyelid adhesions, trichiasis, and severe dry eye

				persisted as ocular sequelae.
Tsai et al. 2023[175]	Case report	SJS/TEN: 1 patient (2 eyes)<br><br>Age: 30 years	Allogeneic conjunctival–limbal stem cell transplantation (CLAL) + MMG+<br><br>Minor salivary gland transplant + AMT + hyperbaric oxygen therapy (HBOT) + topical treatment (steroid, lubricant, autologous serum) Scleral lens	BCVA:<br><br>At Presentation: Light perception OD, hand motion OS, counting fingers.<br><br>At Follow-Up: 0.4 (0.8 with additional scleral lens correction).<br><br>Complete re-epithelialization of the eye was noted at one-month follow-up.
Yoshikawa et al. 2023[176]	Retrospective case series	Total: 41 patients (50 eyes)<br><br>Mean Age: 21.4 ± 16.6 years	Limbal rigid contact lens (CL)	Visual Acuity (VA):<br><br>VA improved in 49 of the 50 eyes and remained unchanged in 1 eye.<br><br>Mean BCVA logMAR improved from 1.60 to 1.04 immediately after initiating CL wear therapy ($P < 0.01$).<br><br>Conjunctival Hyperemia:<br><br>At the final follow-up examination, there were no cases in which conjunctival hyperemia had worsened, and improvement was observed in 24 of the 50 eyes ($P < 0.01$).<br><br>Corneal Neovascularization:<br><br>At 4 and 5 years after initiation of limbal rigid<br><br>CL wear therapy, the mean score had significantly improved compared with at the start of the therapy. At the final follow-up examination, there were no cases in which corneal neovascularization had worsened.
Zhang et al. 2023[177]	Retrospective case series	Total: 5 patients (10 eyes) divided into two groups<br><br>Group A: Amniotic fornical ring (AFR)-assisted AMT; n = 3 patients (6 eyes)<br><br>Mean Age Group A: 23.0 ± 21.7 years<br><br>Group B: Sutured amniotic membrane transplant (SAMT); n = 2	AMT (two techniques)	BCVA:<br><br>Group A:<br><br>Mean Initial: 1.50 ±0.77.<br><br>Mean Final: 0.10 ± 0.13.<br><br>Mean VA Improvement: 1.40 ±0.82.<br><br>Group B:<br><br>Mean Initial: 1.60 ± 0.81.<br><br>Mean Final: 0.00 ± 0.00.<br><br>Mean VA Improvement: 0.90 ± 0.00.<br><br>No significant difference between groups. There was a significant improvement in VA for both groups.<br><br>Mean Percentage Healed Corneal Surface Area

		patients (4 eyes)<br><br>Mean Age Group B: 16.5 ± 0.7 years		(PHCA):<br><br>Group A: 89.92% ± 14.62%.<br><br>Group B: 100.00% ± 0.00%.<br><br>Complications:<br><br>Meibomian Gland Dysfunction: 4 patients.<br><br>Palpebral conjunctival scarring: 3 patients.<br><br>Symblepharon: 1 patient.
Zhu et al. 2023[178]	Retrospective interventional case series	SJS: 1 patient (1 eye)<br><br>Age: 26 years	Oral mucosal epithelial transplantation (OMET)	One-Month Follow-Up: Corneal surface epithelization was incomplete, and intensive neovascularization occurred.<br><br>Seven-Month Follow-Up: Corneal surface epithelization was completed, and the neovascularization was diminished.
Abulfateh et al. 2024[179]	Case report	TEN: 1 patient (2 eyes)<br><br>Age: 10 years	IV steroids<br><br>Oral steroids<br><br>IVIG<br><br>ProKera<br><br>Topical treatment (steroids, ABX, lubricants)	The patient had significant improvement in ocular surface healing and stabilization of conjunctival and corneal conditions.<br><br>BCVA:<br>At Presentation: Not reported.<br><br>At Follow-Up: 6/18 OU.
Aziza et al. 2024[180]	Retrospective case series	SJS: 16 patients (20 eyes)<br><br>Mean Age: 59 ± 15 years	Cultivated oral mucosal epithelial transplantation (COMET) + limbal rigid contact lens (CL)	BCVA:<br><br>The mean BCVA at baseline and at final follow-up was logMAR 1.9±0.5 and 1.3±0.7, respectively ($p<0.05$).<br><br>BCVA improved in 16 eyes.<br><br>BCVA remained the same in 2 eyes.<br><br>BCVA worsened in 2 eyes.<br><br>Ocular Surface Grading Scores (OSGSs):<br><br>OSGSs for symblepharon and upper and lower fornix shortening showed significant improvement<br><br>at each follow-up.<br><br>OSGS improved in 18 eyes.<br><br>OSGS worsened in 2 eyes.
Bai et al. 2024[181]	Case report	SJS: 1 patient (2 eyes)<br><br>Age: 52 years	IV steroids<br><br>Oral steroids<br><br>Topical treatment (ABX, steroids)	BCVA:<br><br>At Presentation: 1.0 OD, 0.8 OS.<br><br>At Discharge: 1.2 OD, 1.0 OS.<br><br>After 12 days of treatment, the corneas of both eyes became transparent, without any edema or crude

				epithelia.
Chen et al. 2024[182]	Case report	SJS: 1 patient (2 eyes)<br><br>Age: 21 years	ProKera<br><br>Topical treatment (ABX, steroid)	BCVA:<br><br>At Presentation: 20/630 OD, 20/320 OS.<br><br>At Follow-Up: 20/25 OU.<br><br>Corneal re-epithelization was observed in both eyes after two weeks. However, a conjunctival growth on the AM and presence of symblepharon developed on the upper lid margin of the left eye three weeks after ProKera.<br><br>Consequently, the patient underwent symblepharon release and removal of ProKera and the adhered vascularized tissue.<br><br>At the 3-month follow-up, corneal epithelial defect healed, with minimal subepithelial fibrosis. Both fornices were deep, without formation of symblepharon.
Hui Xian Foo et al. 2024[183]	Retrospective cohort study	Total: 18 patients (36 eyes); SJS/TEN patients divided into two groups<br><br>IVIG Group: 8 patients<br><br>CsA Group: 10 patients<br><br>Mean Age: 43 years (31–69)	IVIG<br><br>Systemic cyclosporin (CsA)	The CsA group had a trend towards worse overall chronic ocular surface complication scores compared to the IVIG group (median [IQR]: 2 [0–3] vs. 1 [0–6.5], p = 0.27).In the CsA group, 2/10 had to undergo further ocular surgical interventions.<br><br>In the IVIG group, 0/8 patients required long-term surgical interventions.<br><br>Complications:<br><br>CsA Group:<br><br>In the CsA group, 6/10 patients had a poor ocular surface requiring punctal plugs or topical CsA.<br><br>In the CsA group, 4/10 patients had eyelid complications including trichiasis, distichiasis, cicatricial entropion, and symblepharon.<br><br>IVIG Group:<br><br>In the IVIG group, 1/8 patients developed distichiasis and symblepharon.<br><br>Compared to those who received IVIG, SJS/TEN patients who received CsA at the acute disease stage seemed to have worse acute corneal and chronic corneal and eyelid complications.
Gueudry et al. 2024[184]	Case series	TEN: 3 patients (4 eyes)<br><br>Mean Age: 45.3 years	Topical treatment (Wharton's jelly eye drops)	Corneal ulcer improvement was noticed from 24 to 48 h after the onset of the Wharton's jelly eye drops. Complete healing was assessed after 6–8 days.
Kwong et al. 2024[185]	Case report	SJS: 1 patient (2 eyes)<br><br>Age: 10 years	IVIG<br>IV steroid<br><br>Oral cyclosporin<br><br>AMT	Re-epithelization occurred on day 13/<br><br>At the last follow-up, at 7 weeks from the disease onset, he had no ocular sequelae.

			Etanercept (subcutaneous injection)	
Pan et al. 2024[186]	Retrospective cohort study	Total: 58 patients (116 eyes) divided into 2 groups<br><br>Etanercept Group: 29 patients (58 eyes)<br><br>Mean Age Etanercept Group: 52.8 ± 16.4 years<br><br>Prednisolone Group: 29 patients (58 eyes)<br><br>Mean Age Prednisolone Group: 51.9 ± 24.2 years	Etanercept (subcutaneous)<br><br>IV steroid (prednisolone)	The etanercept group had better best corrected visual acuity and Schirmer test values (Table 1) than the prednisolone group. Sotozono et al.'s mean total chronic<br><br>OSGS in the etanercept group was considerably lower than in the prednisolone group.<br><br>Those treated with etanercept had better outcomes than those treated with prednisolone in nearly all aspects of the OSGS, best corrected visual acuity, and Schirmer test.
Peng et al. 2024[187]	Retrospective case series	SJS: 18 patients (24 eyes)<br><br>Mean Age: 36.7 ± 17.1 years	Keratoplasty (n = 9)<br><br>Keratolimbal allograft (KLAL) (n = 8)<br>Combined surgery (n = 12)<br><br>Additional treatment for corneal epithelialization<br><br>Autologous serum eye drops (n = 10 eyes)<br><br>Contact lens (n<br><br>= 15 eyes)<br><br>AMT (n = 6 eyes)<br><br>Tarsorrhaphy (n = 8 eyes)	The success rate of the interventions was 86.7%.<br><br>BCVA:<br><br>The mean optimal visual acuity (VA) (0.74 ± 0.60 logMAR) and mean endpoint VA (1.06 ± 0.82 logMAR) were both significantly better than the mean preoperative VA (1.96 ± 0.43 logMAR) (95% CI, p = 0.000).<br><br>Preoperatively, the VA of 14 patients was in the low vision spectrum, and 9 patients were blind. After surgeries, 57.1% patients (8/14) were no longer in the low vision spectrum, and 88.9% patients (8/9) were no longer blind.<br><br>Mean epithelialization time was 7.1 ± 7.6 (range,<br><br>1–28).<br><br>Complications:<br><br>After epithelialization, 13 eyes had a stable corneal epithelium, whereas superficial punctate keratopathy was recorded in 9 eyes.<br><br>One graft rejection was observed.
Rashad et al. 2024[188]	Retrospective cohort study	Total: 23 patients (45 eyes) divided into two groups<br><br>AMT Suture: 14 patients (27 eyes)<br><br>Mean Age	AMT (2 techniques: sutured and sutureless)	Eleven out of twenty-seven eyes (40.7%) in the suture group developed a severe ocular complication, versus seven out of eighteen eyes (38.9%) in the sutureless group, with no significant difference (p=1.000).<br><br>The was no difference in mean BCVA at the most recent visit between groups (p=0.5112).<br><br>The sutureless technique for AMT is as effective as the sutured technique in stabilizing the ocular

		Suture Group: 28.9 ± 12.6 years<br><br>AMT Sutureless: 9 patients (18 eyes)<br><br>Mean Age Sutureless Group: 28.3 ± 21.9 years		surface and mitigating chronic ocular complications in SJS/TEN.
Sharma et al. 2024[189]	Ambispective interventional case series	SJS: 20 patients (20 eyes)<br><br>Mean Age: 28.6 ± 13.1 years (range 12–70)	Minor salivary gland transplantation	BCVA:<br>There was a significant improvement in mean postoperative best corrected visual acuity (BCVA) in 10 of 20 patients (50 % cases).<br><br>Preoperative BCVA: 1.99 ±0.57 logMAR.<br><br>Postoperative BCVA (1 year): 1.76 ±0.41 logMAR (p = 0.002).<br><br>Schirmer test scores improved significantly in 15 eyes (75 %) (p=0.0004).<br><br>Hyperemia score improved from 2.3 to 1.4 and all eyes were non-inflamed at 1-year follow-up (p = 0.0004).<br><br>Corneal epithelial defect score showed significant improvement from its baseline value (p =0.0004).<br><br>Corneal opacification score decreased significantly (p = 0.001), which led to improved corneal transparency in 14 out of 20 eyes (70 % cases).<br><br>Corneal neovascularization score (p =0.001), corneal keratinization score (p =0.04), and palisades of Vogt score (p =0.007) also showed significant improvements.<br><br>No sight-threatening complications occurred after surgery.
Sharma et al. 2024[190]	Randomized controlled trial	SJS: 44 patients (88 eyes) divided into 2 groups<br><br>Group A (n=44 eyes): Topical CsA 1% + standard therapy<br><br>Mean Age Group A: 23.9 ± 15.1 years<br><br>Group B (n=44 eyes): Patients received topical saline drops + standard	Topical cyclosporine A 1% (CsA)	At 6 months, both groups showed a significant improvement in the mean severity grades of conjunctival hyperemia (A, p=0.001; B, p=0.0001), mucocutaneous<br><br>junction involvement (A, p=0.001; B, p=0.0001), and meibomian gland involvement (A, p = 0.0471; B, p=0.006).<br><br>Corneal neovascularization and corneal keratinization significantly worsened over 6 months in both groups.<br><br>Mean BCVA:<br><br>Group A:<br><br>At Presentation: 1.2±0.9 logMAR.<br><br>At Follow-Up (6 months): 1.11 ± 0.82 logMAR.

		therapy<br><br>Mean Age Group B: 26.0 ± 18.7 years		Group B:<br><br>At Presentation: 1.5±1.0 logMAR.<br><br>At Follow-Up (6 months): 1.34 ± 0.92 logMAR.<br><br>At baseline, both groups were comparable in terms of BCVA. At 6 months of follow-up, the values continued to be comparable. with no significant difference between the two groups.<br><br>Adjuvant treatment with topical CsA is not superior to standard therapy in cases of acute SJS.
Soma et al. 2024[191]	Non-randomized single-arm clinical study	TEN: 1 patient (1 eye)<br><br>Age: 39 years	Limbal stem cell transplant (induced pluripotent stem cell-derived corneal epithelium)	BCVA:<br><br>At Presentation: 1.68 logMAR.<br><br>At Follow-Up (52 weeks): 1.40 logMAR.<br><br>LSCD Severity:<br><br>At Presentation: 3.<br><br>At Follow-Up (52 weeks): 2B.<br><br>Corneal Opacification:<br><br>At Presentation: 3.0.<br><br>At Follow-Up (52 weeks): 1.6.<br><br>No serious adverse events.
Subramanian et al. 2024[192]	Case report	SJS: 1 patient (1 eye)<br><br>Age: 21 years	Scleral lens + prosthetic type-D contact lens (piggyback system; scleral contact lenses over a prosthetic contact lens)	Ocular Surface Disease Index (OSDI) Questionnaire:<br><br>At Presentation: 50 (indicated severe dry eye).<br><br>At Follow-Up (3 months): 37.<br><br>Subjective improvement in comfort, cosmesis, and reduction in photophobia.
Wróblewska-Czajka et al. 2024[193]	Retrospective interventional case series	SJS: 4 patients (4 eyes)<br><br>Mean Age: 56.8 ± 14.2 years	Keratoprosthesis (Boston Type 1)	BCVA:<br>After 2 years post-KPro, 50% (2/4 patients) achieved a BCVA better than 0.1.<br><br>Complications:<br><br>One out of four patients had corneal melting;<br><br>Four out of four patients had retroprosthetic membrane.
Yao et al. 2024[194]	Retrospective case series	SJS: 2 patients (2 eyes)<br><br>Mean Age: 46 years	MMG (tarsal patch graft)	BCVA<br><br>Patient 1:<br><br>At Presentation: 6/48 OD.<br><br>At Follow-Up: 6/12 OD.<br><br>Patient 2:<br><br>At Presentation: 6/90 OS.

				At Follow-Up: 6/30 OS.<br><br>No recurrences of corneal epithelial defects or local tarsal conjunctival pathologies.<br><br>The procedure was well tolerated, with no reported complications to graft or donor sites. All patients reported symptomatic improvement.

HM = Hand motion, LP = Light Perception, BCVA = best corrected visual acuity,

Supplemental References (all 194 citations) REFERENCES

1. Tseng SCG, Prabhasawat P, Barton K, Gray T, Meiler D. Amniotic membrane transplantation with or without limbal allografts for corneal surface reconstruction in patients with limbal stem cell deficiency. *Archives of Ophthalmology*. 1998;116(4):431-441. doi:10.1001/archopht.116.4.431

2. Dua HS, Azuara-Blanco A. Allo-limbal transplantation in patients with limbal stem cell deficiency. *Br J Ophthalmol*. 1999;83(4):414-9. doi:10.1136/bjo.83.4.414

3. Rao SK, Rajagopal R, Sitalakshmi G, Padmanabhan P. Limbal allografting from related live donors for corneal surface reconstruction. *Ophthalmology*. 1999;106(4):822-8. doi:10.1016/S0161-6420(99)90173-2

4. Tsubota K, Shimazaki J. Surgical treatment of children blinded by Stevens-Johnson syndrome. *Am J Ophthalmol*. 1999;128(5):573-81. doi:10.1016/s0002-9394(99)00224-x

5. Honavar SG, Bansal AK, Sangwan VS, Rao GN. Amniotic membrane transplantation for ocular surface reconstruction in Stevens-Johnson syndrome. *Ophthalmology*. 2000;107(5):975-9. doi:10.1016/s0161-6420(00)00026-9

6. Romero-Rangel T, Stavrou P, Cotter J, Rosenthal P, Baltatzis S, Foster CS. Gas-permeable scleral contact lens therapy in ocular surface disease. *American Journal of Ophthalmology*. 2000;130(1):25-32. doi:10.1016/S0002-9394(00)00378-0

7. Rosenthal P, Cotter JM, Baum J. Treatment of persistent corneal epithelial defect with extended wear of a fluid-ventilated gas-permeable scleral contact lens. *Am J Ophthalmol*. 2000;130(1):33-41. doi:10.1016/s0002-9394(00)00379-2

8. Shimazaki J, Shimmura S, Fujishima H, Tsubota K. Association of preoperative tear function with surgical outcome in severe Stevens-Johnson syndrome. *Ophthalmology*. 2000;107(8):1518-23. doi:10.1016/s0161-6420(00)00214-1

9. Daya SM, Ilari FA. Living related conjunctival limbal allograft for the treatment of stem cell deficiency. *Ophthalmology*. 2001;108(1):126-33; discussion 133. doi:10.1016/s0161-6420(00)00475-9

10. Koizumi N, Inatomi T, Suzuki T, Sotozono C, Kinoshita S. Cultivated corneal epithelial stem cell transplantation in ocular surface disorders. *Ophthalmology*. 2001;108(9):1569-74. doi:10.1016/s0161-6420(01)00694-7

11. Tappin MJ, Pullum KW, Buckley RJ. Scleral contact lenses for overnight wear in the management of ocular surface disorders. *Eye (Lond)*. 2001;15(Pt 2):168-72. doi:10.1038/eye.2001.54

12. John T, Foulks GN, John ME, Cheng K, Hu D. Amniotic membrane in the surgical management of acute toxic epidermal necrolysis. *Ophthalmology*. 2002;109(2):351-360. doi:10.1016/S0161-6420(01)00900-9

13. Samson CM, Nduaguba C, Baltatzis S, Foster CS. Limbal stem cell transplantation in chronic inflammatory eye disease. *Ophthalmology*. 2002;109(5):862-868. doi:10.1016/S0161-6420(02)00994-6

14. Shimazaki J, Aiba M, Goto E, Kato N, Shimmura S, Tsubota K. Transplantation of human limbal epithelium cultivated on amniotic membrane for the treatment of severe ocular surface disorders. *Ophthalmology*. 2002;109(7):1285-90. doi:10.1016/s0161-6420(02)01089-8

15. Solomon A, Meller D, Prabhasawat P, et al. Amniotic membrane grafts for nontraumatic corneal perforations, descemetoceles, and deep ulcers. *Ophthalmology*. 2002;109(4):694-703. doi:10.1016/s0161-6420(01)01032-6

16. Tsubota K, Shimmura S, Shinozaki N, Holland EJ, Shimazaki J. Clinical application of living-related conjunctival-limbal allograft. *Am J Ophthalmol*. 2002;133(1):134-5. doi:10.1016/s0002-9394(01)01208-9

17. Geerling G, Liu CS, Dart JK, Sieg P, Herold J, Collin JR. Sight and comfort: complex procedures in end-stage Stevens-Johnson syndrome. *Eye (Lond)*. 2003;17(1):89-91. doi:10.1038/sj.eye.6700264

18. Gomes JA, Santos MS, Ventura AS, Donato WB, Cunha MC, Höfling-Lima AL. Amniotic membrane with living related corneal limbal/conjunctival allograft for ocular surface reconstruction in Stevens-Johnson syndrome. *Arch Ophthalmol*. 2003;121(10):1369-74. doi:10.1001/archopht.121.10.1369

19. Park EH, Korn TS, Vasani SN, Kikkawa DO. Autologous allogeneic amniotic membrane grafting in Stevens-Johnson syndrome. *Ophthalmic Plastic and Reconstructive Surgery*. 2003;19(3):250-251. doi:10.1097/01.IOP.0000066652.91232.39

20. Solomon A, Espana EM, Tseng SCG. Amniotic membrane transplantation for reconstruction of the conjunctival fornices. *Ophthalmology*. 2003;110(1):93-100. doi:10.1016/S0161-6420(02)01441-0

21. Goldberg D, Panigrahi D, Barazi M, Abelson M, Butrus S. A case of rofecoxib-associated stevens-johnson syndrome with corneal and conjunctival changes. *Cornea*. 2004;23(7):736-7. doi:10.1097/01.ico.0000126330.77228.a3

22. Kaido M, Goto E, Dogru M, Tsubota K. Punctal occlusion in the management of chronic Stevens-Johnson syndrome. *Ophthalmology*. 2004;111(5):895-900. doi:10.1016/j.ophtha.2003.09.034

23. Lam NS, Yang YH, Wang LC, Lin YT, Chiang BL. Clinical characteristics of childhood erythema multiforme, Stevens-Johnson syndrome and toxic epidermal necrolysis in Taiwanese children. *Journal of microbiology, immunology, and infection = Wei mian yu gan ran za zhi*. 2004;37(6):366-370.

24. Nakamura T, Inatomi T, Sotozono C, Amemiya T, Kanamura N, Kinoshita S. Transplantation of cultivated autologous oral mucosal epithelial cells in patients with severe ocular surface disorders. *Br J Ophthalmol*. 2004;88(10):1280-4. doi:10.1136/bjo.2003.038497

25. Fukuda M, Nakao A, Hamada S, Liu C, Shimomura Y. A case of severe Stevens-Johnson syndrome successfully treated by osteo-odonto-keratoprosthesis surgery. *Jpn J Ophthalmol*. 2005;49(5):423-4. doi:10.1007/s10384-004-0215-3

26. Yip LW, Thong BY, Tan AW, Khin LW, Chng HH, Heng WJ. High-dose intravenous immunoglobulin in the treatment of toxic epidermal necrolysis: A study of ocular benefits. *Eye*. 2005;19(8):846-853. doi:10.1038/sj.eye.6701653

27. Kobayashi A, Yoshita T, Sugiyama K, et al. Amniotic membrane transplantation in acute phase of toxic epidermal necrolysis with severe corneal involvement. *Ophthalmology*. 2006;113(1):126-132. doi:10.1016/j.ophtha.2005.09.001

28. Nakamura T, Inatomi T, Sotozono C, et al. Transplantation of autologous serum-derived cultivated corneal epithelial equivalents for the treatment of severe ocular surface disease. *Ophthalmology*. 2006;113(10):1765-72. doi:10.1016/j.ophtha.2006.04.030

29. Ang LP, Sotozono C, Koizumi N, Suzuki T, Inatomi T, Kinoshita S. A comparison between cultivated and conventional limbal stem cell transplantation for Stevens-Johnson syndrome. 2007;143(1):178-180.

30. Chang YS, Huang FC, Tseng SH, Hsu CK, Ho CL, Sheu HM. Erythema multiforme, Stevens-Johnson Syndrome, and toxic epidermal necrolysis: Acute ocular manifestations, causes, and management. *Cornea*. 2007;26(2):123-129. doi:10.1097/ICO.0b013e31802eb264

31. Victoria De Rojas M, Dart JKG, Saw VPJ. The natural history of Stevens-Johnson syndrome: Patterns of chronic ocular disease and the role of systemic immunosuppressive therapy. *British Journal of Ophthalmology*. 2007;91(8):1048-1053. doi:10.1136/bjo.2006.109124

32. Tandon A, Cackett P, Mulvihill A, Fleck B. Amniotic membrane grafting for conjunctival and lid surface disease in the acute phase of toxic epidermal necrolysis. *Journal of AAPOS*. 2007;11(6):612-613. doi:10.1016/j.jaapos.2007.04.020

33. Yip LW, Thong BY, Lim J, et al. Ocular manifestations and complications of Stevens-Johnson syndrome and toxic epidermal necrolysis: An Asian series. *Allergy: European Journal of Allergy and Clinical Immunology*. 2007;62(5):527-531. doi:10.1111/j.1398-9995.2006.01295.x

34. Jain V, Shome D, Natarajan S. Nevirapine-induced Stevens-Johnson syndrome in an HIV patient. *Cornea*. 2008;27(3):366-7. doi:10.1097/ICO.0b013e31815b941b

35. Sayegh RR, Ang LP, Foster CS, Dohlman CH. The Boston keratoprosthesis in Stevens-Johnson syndrome. *Am J Ophthalmol*. 2008;145(3):438-44. doi:10.1016/j.ajo.2007.11.002

36. Uy HS, Chan PS, Ang RE. Topical bevacizumab and ocular surface neovascularization in patients with stevens-johnson syndrome. *Cornea*. 2008;27(1):70-3. doi:10.1097/ICO.0b013e318158f6ad

37. Araki Y, Sotozono C, Inatomi T, et al. Successful Treatment of Stevens-Johnson Syndrome with Steroid Pulse Therapy at Disease Onset. *American Journal of Ophthalmology*. 2009;147(6):1004-1011.e1. doi:10.1016/j.ajo.2008.12.040

38. Sotozono C, Ueta M, Koizumi N, et al. Diagnosis and Treatment of Stevens-Johnson Syndrome and Toxic Epidermal Necrolysis with Ocular Complications. *Ophthalmology*. 2009;116(4):685-690. doi:10.1016/j.ophtha.2008.12.048

39. Tougeron-Brousseau B, Delcampe A, Gueudry J, et al. Vision-Related Function After Scleral Lens Fitting in Ocular Complications of Stevens-Johnson Syndrome and Toxic Epidermal Necrolysis. *American Journal of Ophthalmology*. 2009;148(6):852-859.e2. doi:10.1016/j.ajo.2009.07.006

40. Iyer G, Pillai VS, Srinivasan B, Guruswami S, Padmanabhan P. Mucous membrane grafting for lid margin keratinization in Stevens–Johnson syndrome: results. *Cornea*. 2010;29(2):146-51. doi:10.1097/ICO.0b013e3181ae2691

41. Marinho DR, Burmann TG, Kwitko S. Labial salivary gland transplantation for severe dry eye due to chemical burns and Stevens-Johnson syndrome. *Ophthalmic Plast Reconstr Surg*. 2010;26(3):182-4. doi:10.1097/IOP.0b013e3181b8c3ad

42. Shammas MC, Lai EC, Sarkar JS, Yang J, Starr CE, Sippel KC. Management of Acute Stevens-Johnson Syndrome and Toxic Epidermal Necrolysis Utilizing Amniotic Membrane and Topical Corticosteroids. *American Journal of Ophthalmology*. 2010;149(2):203-213.e2. doi:10.1016/j.ajo.2009.08.040

43. Shay E, Khadem JJ, Tseng SCG. Efficacy and limitation of sutureless amniotic membrane transplantation for acute toxic epidermal necrolysis. *Cornea*. 2010;29(3):359-361. doi:10.1097/ICO.0b013e3181acf816

44. Das JK, Medhi J, Chakravarty R, Soibam R. Mucous membrane grafting for the post-Steven-Johnson syndrome symblepharon: a case report. *Indian J Ophthalmol*. 2011;59(3):231-3. doi:10.4103/0301-4738.81039

45. Finkelstein Y, Soon GS, Acuna P, et al. Recurrence and outcomes of Stevens-Johnson syndrome and toxic epidermal necrolysis in children. *Pediatrics*. 2011;128(4):723-8. doi:10.1542/peds.2010-3322

46. Gregory DG. Treatment of acute stevensjohnson syndrome and toxic epidermal necrolysis using amniotic membrane: A review of 10 consecutive cases. *Ophthalmology*. 2011;118(5):908-914. doi:10.1016/j.ophtha.2011.01.046

47. Lau B, Mutyala D, Dhaliwal D. A case report of doxycycline-induced Stevens-Johnson syndrome. *Cornea*. 2011;30(5):595-7. doi:10.1097/ICO.0b013e3181f05773

48. Liu J, Sheha H, Fu Y, Giegengack M, Tseng SCG. Oral mucosal graft with amniotic membrane transplantation for total limbal stem cell deficiency. *American Journal of Ophthalmology*. 2011;152(5):739-74.e1. doi:10.1016/j.ajo.2011.03.037

49. Onaran Z, Usta G, Koçak M, Örnek K, Büyükkoçak U. Topical ophthalmic cyclosporine in the treatment of toxic epidermal necrolysis. *Case Reports in Medicine*. 2011;2011((Onaran Z., drzaferonaran@yahoo.com; Usta G., dr.gulsah_usta@hotmail.com; Örnek K., kemalornek@hotmail.com) Department of Ophthalmology, Krkkale University, School of Medicine, Krkkale 71100, Turkey(Koçak M., mukadderkocak@gmail.com) Department of Dermato)doi:10.1155/2011/416842

50. Pujari S, Siddique SS, Dohlman CH, Chodosh J. The boston keratoprosthesis type II: The massachusetts eye and ear infirmary experience. *Cornea*. 2011;30(12):1298-1303. doi:10.1097/ICO.0b013e318215207c

51. Satake Y, Higa K, Tsubota K, Shimazaki J. Long-term outcome of cultivated oral mucosal epithelial sheet transplantation in treatment of total limbal stem cell deficiency. *Ophthalmology*. 2011;118(8):1524-30. doi:10.1016/j.ophtha.2011.01.039

52. Sc R, I T, T TD. Osteo-odonto keratoprosthesis in Stevens-Johnson syndrome: a case report. *Int J Ophthalmol*. 2011;4(2):212-5. doi:10.3980/j.issn.2222-3959.2011.02.23

53. Uy HS, Yu EN, Sua AS. Histologic findings of bevacizumab-treated human conjunctiva in Stevens-Johnson syndrome. *Cornea*. 2011;30(11):1273-6. doi:10.1097/ICO.0b013e3181cb83f2

54. Yagi T, Sotozono C, Tanaka M, et al. Cytokine storm arising on the ocular surface in a patient with Stevens - Johnson syndrome. *British Journal of Ophthalmology*. 2011;95(7):1030-1031. doi:10.1136/bjo.2010.196295

55. Barua A, McKee HD, Barbara R, Carley F, Biswas S. Toxic epidermal necrolysis in a 15-month-old girl successfully treated with amniotic membrane transplantation. *Journal of AAPOS*. 2012;16(5):478-480. doi:10.1016/j.jaapos.2012.05.011

56. Hess TM, Chew HF. Successful treatment of acute ocular involvement in Stevens-Johnson syndrome with amniotic membrane transplantation: A case report. *Can J Ophthalmol*. 2012;47(6):e44-e46. doi:10.1016/j.jcjo.2012.04.016

57. Hsu M, Jayaram A, Verner R, Lin A, Bouchard C. Indications and outcomes of amniotic membrane transplantation in the management of acute stevens-johnson syndrome and toxic

epidermal necrolysis: A case-control study. *Cornea*. 2012;31(12):1394-1402. doi:10.1097/ICO.0b013e31823d02a8

58. Huang Y, Dong Y, Wang L, et al. Long-term outcomes of MICOF keratoprosthesis in the end stage of autoimmune dry eyes: an experience in China. *Br J Ophthalmol*. 2012;96(1):28-33. doi:10.1136/bjo.2010.193029

59. Kesarwani S, Sahu SK, Basu S. Bilateral response after unilateral subconjunctival bevacizumab injection in a child with Stevens-Johnson syndrome. *J AAPOS*. 2012;16(3):309-11. doi:10.1016/j.jaapos.2011.12.153

60. Sant' Anna AE, Hazarbassanov RM, de Freitas D, Gomes J. Minor salivary glands and labial mucous membrane graft in the treatment of severe symblepharon and dry eye in patients with Stevens-Johnson syndrome. *Br J Ophthalmol*. 2012;96(2):234-9. doi:10.1136/bjo.2010.199901

61. Ciralsky JB, Sippel KC. Prompt versus delayed amniotic membrane application in a patient with acute Stevens-Johnson syndrome. *Clinical Ophthalmology*. 2013;7((Ciralsky J.B., jessciralsky@gmail.com; Sippel K.C.) Department of Ophthalmology, Weill Cornell Medical College, New York, NY, United States):1031-1034. doi:10.2147/opth.s45054

62. Kim KH, Park SW, Kim MK, Wee WR. Effect of age and early intervention with a systemic steroid, intravenous immunoglobulin or amniotic membrane transplantation on the ocular outcomes of patients with Stevens-Johnson syndrome. *Korean journal of ophthalmology : KJO*. 2013;27(5):331-340. doi:10.3341/kjo.2013.27.5.331

63. Kolomeyer AM, Do BK, Tu Y, Chu DS. Placement of ProKera in the management of ocular manifestations of acute Stevens-Johnson syndrome in an outpatient. *Eye Contact Lens*. 2013;39(3):e7-11. doi:10.1097/ICL.0b013e318255124f

64. Ling JD, Gire A, Pflugfelder SC. PROSE therapy used to minimize corneal trauma in patients with corneal epithelial defects. *Am J Ophthalmol*. 2013;155(4):615-619, 619.e1. doi:10.1016/j.ajo.2012.09.033

65. Md Noh UK, Then KY. Spontaneous bilateral corneal perforation in stevens- johnsons syndrome-a challenge in management. *Malays J Med Sci*. 2013;20(1):84-7.

66. Prabhasawat P, Tesavibul N, Karnchanachetanee C, Kasemson S. Efficacy of cyclosporine 0.05% eye drops in Stevens Johnson syndrome with chronic dry eye. *J Ocul Pharmacol Ther*. 2013;29(3):372-7. doi:10.1089/jop.2012.0009

67. Sotozono C, Inatomi T, Nakamura T, et al. Visual improvement after cultivated oral mucosal epithelial transplantation. *Ophthalmology*. 2013;120(1):193-200. doi:10.1016/j.ophtha.2012.07.053

68. Tomlins PJ, Parulekar MV, Rauz S. "Triple-TEN" in the treatment of acute ocular complications from toxic epidermal necrolysis. *Cornea*. 2013;32(3):365-369. doi:10.1097/ICO.0b013e318243fee3

69. Basu S, Sureka S, Shukla R, Sangwan V. Boston type 1 based keratoprosthesis (Auro Kpro) and its modification (LVP Kpro) in chronic Stevens Johnson syndrome. *BMJ Case Rep*. 2014;2014doi:10.1136/bcr-2013-202756

70. de Oliveira LA, Pedreira Magalhães F, Hirai FE, de Sousa LB. Experience with Boston keratoprosthesis type 1 in the developing world. *Can J Ophthalmol*. 2014;49(4):351-7. doi:10.1016/j.jcjo.2014.05.003

71. Heur M, Bach D, Theophanous C, Chiu GB. Prosthetic replacement of the ocular surface ecosystem scleral lens therapy for patients with ocular symptoms of chronic Stevens-Johnson syndrome. *Am J Ophthalmol*. 2014;158(1):49-54. doi:10.1016/j.ajo.2014.03.012

72. Iyer G, Srinivasan B, Agarwal S, Kamala Muralidharan S, Arumugam S. Comprehensive approach to ocular consequences of Stevens Johnson Syndrome - the aftermath of a systemic condition. *Graefes Arch Clin Exp Ophthalmol*. 2014;252(3):457-67. doi:10.1007/s00417-014-2568-8

73. López-García JS, Rivas L, García-Lozano I, Conesa E, Elosua I, Murube J. Amniotic membrane transplantation in acute toxic epidermal necrolysis: Histopathologic changes and ocular surface features after 1-year follow-up. *European Journal of Ophthalmology*. 2014;24(5):667-675. doi:10.5301/ejo.5000434

74. Pruet CM, Queen JH, Kim G. Amnion doughnut: a novel method for sutureless fixation of amniotic membrane to the bulbar and palpebral conjunctiva in acute ocular-involving Stevens-Johnson syndrome. *Cornea*. 2014;33(11):1240-4. doi:10.1097/ICO.0000000000000254

75. Sotozono C, Yamauchi N, Maeda S, Kinoshita S. Tear Exchangeable Limbal Rigid Contact Lens for Ocular Sequelae Resulting From Stevens-Johnson Syndrome or Toxic Epidermal Necrolysis. *American Journal of Ophthalmology*. 2014;((Sotozono C., csotozon@koto.kpu-m.ac.jp; Kinoshita S.) Department of Ophthalmology, Kyoto Prefectural University of Medicine, Kyoto, Japan(Yamauchi N.; Maeda S.) Sun Contact Lens Co, Ltd, Kyoto, Japan)doi:10.1016/j.ajo.2014.07.012

76. Sotozono C, Inatomi T, Nakamura T, et al. Cultivated oral mucosal epithelial transplantation for persistent epithelial defect in severe ocular surface diseases with acute inflammatory activity. *Acta Ophthalmol*. 2014;92(6):e447-53. doi:10.1111/aos.12397

77. Wang F, Li S, Wang T, Gao H, Shi W. Modified tectonic keratoplasty with minimal corneal graft for corneal perforation in severe Stevens--Johnson syndrome: a case series study. *BMC Ophthalmol*. 2014;14:97. doi:10.1186/1471-2415-14-97

78. Agrawal A, Pratap VB. Amniotic membrane transplantation (AMT) without the use of sutures/fibrin glue. *Nepal J Ophthalmol*. 2015;7(14):173-7. doi:10.3126/nepjoph.v7i2.14969

79. Alexander JK, Basak SK, Padilla MD, Yu F, Aldave AJ. International Outcomes of the Boston Type I Keratoprosthesis in Stevens-Johnson Syndrome. *Cornea*. 2015;34(11):1387-94. doi:10.1097/ICO.0000000000000619

80. Papakostas TD, Le HG, Chodosh J, Jacobs DS. Prosthetic replacement of the ocular surface ecosystem as treatment for ocular surface disease in patients with a history of stevens-johnson syndrome/toxic epidermal necrolysis. *Ophthalmology*. 2015;122(2):248-253. doi:10.1016/j.ophtha.2014.08.015

81. Catt CJ, Hamilton GM, Fish J, Mireskandari K, Ali A. Ocular Manifestations of Stevens-Johnson Syndrome and Toxic Epidermal Necrolysis in Children. *American Journal of Ophthalmology*. 2016;166((Catt C.J.; Hamilton G.M.; Fish J.; Mireskandari K.; Ali A., asim.ali@sickkids.ca) Department of Ophthalmology and Vision Sciences (C.J.C., K.M., A.A.), Burn Unit, Division of Plastic Surgery (J.F.), The Hospital for Sick Children, Faculty of Medicine (C.):68-75. doi:10.1016/j.ajo.2016.03.020

82. Cheung CSY, Ali A, Chew HF. Successful treatment of acute ocular-involving toxic epidermal necrolysis using amniotic membrane suture fixated to custom designed symblepharon rings. *Cornea*. 2016;35(4):578-581. doi:10.1097/ICO.0000000000000770

83. Gregory DG. New Grading System and Treatment Guidelines for the Acute Ocular Manifestations of Stevens-Johnson Syndrome. *Ophthalmology*. Aug 2016;123(8):1653-1658. doi:10.1016/j.ophtha.2016.04.041

84. Iyer G, Srinivasan B, Agarwal S, Pillai VS, Ahuja A. Treatment Modalities and Clinical Outcomes in Ocular Sequelae of Stevens-Johnson Syndrome Over 25 Years--A Paradigm Shift. *Cornea*. 2016;35(1):46-50. doi:10.1097/ICO.0000000000000680

85. La Porta Weber S, Becco de Souza R, Gomes JÁ P, Hofling-Lima AL. The Use of the Esclera Scleral Contact Lens in the Treatment of Moderate to Severe Dry Eye Disease. *Am J Ophthalmol*. 2016;163:167-173.e1. doi:10.1016/j.ajo.2015.11.034

86. Ma KN, Thanos A, Chodosh J, Shah AS, Mantagos IS. A Novel Technique for Amniotic Membrane Transplantation in Patients with Acute Stevens-Johnson Syndrome. *Ocular Surface*. 2016;14(1):31-36. doi:10.1016/j.jtos.2015.07.002

87. Sharma N, Thenarasun SA, Kaur M, et al. Adjuvant Role of Amniotic Membrane Transplantation in Acute Ocular Stevens-Johnson Syndrome: a Randomized Control Trial. 2016;123(3):484-491. doi:10.1016/j.ophtha.2015.10.027

88. Ahmad MS, Frank GS, Hink EM, Palestine AG, Gregory DG, McCourt EA. Amniotic membrane transplants in the pediatric population. *J AAPOS*. 2017;21(3):215-218. doi:10.1016/j.jaapos.2017.04.002

89. Ma X, Xiang R, Meng X, et al. Russian Keratoprosthesis in Stevens-Johnson Syndrome. *Cornea*. 2017;36(3):304-309. doi:10.1097/ICO.0000000000001094

90. Pinna A, Nuvoli E, Blasetti F, Posadinu MA, Boscia F. Plasmapheresis, intravenous immunoglobulins, and autologous serum eyedrops in the acute eye complications of toxic epidermal necrolysis. *European Journal of Ophthalmology*. 2017;27(6):658-663. doi:10.5301/ejo.5000923

91. Barry RJ, Zanetto U, Kolli S, Morjaria R. Toxic epidermal necrolysis: the red eye and red herrings in casualty. *BMJ Case Rep*. 2018;2018doi:10.1136/bcr-2018-225861

92. Basu S, Shanbhag SS, Gokani A, Kedar R, Bahuguna C, Sangwan VS. Chronic Ocular Sequelae of Stevens-Johnson Syndrome in Children: Long-term Impact of Appropriate Therapy on Natural History of Disease. *Am J Ophthalmol*. 2018;189:17-28. doi:10.1016/j.ajo.2018.01.028

93. Chan F, Benson MD, Plemel DJA, Mahmood MN, Chan SM. A diagnosis of Stevens-Johnson Syndrome (SJS) in a patient presenting with superficial keratitis. *Am J Ophthalmol Case Rep*. 2018;11:167-169. doi:10.1016/j.ajoc.2018.06.004

94. Filippi L, de Libero C, Zamma Gallarati B, Fortunato P, Piozzi E. Propranolol eye drops in patients with corneal neovascularization. *Medicine (Baltimore)*. 2018;97(45):e13002. doi:10.1097/MD.0000000000013002

95. Gu J, Zhai J, Liao G, Chen J. Boston Type I Keratoprosthesis Implantation Following Autologous Submandibular Gland Transplantation for End Stage Ocular Surface Disorders. *Ocul Immunol Inflamm*. 2018;26(3):452-455. doi:10.1080/09273948.2016.1234624

96. Kalhorn AJ, Tawse KL, Shah AA, Jung JL, Gregory DG, McCourt EA. Maternal Serum Eye Drops in the Management of Pediatric Persistent Corneal Epithelial Defects: A Case Series. *Cornea*. 2018;37(7):912-915. doi:10.1097/ICO.0000000000001512

97. Nguyen MTB, Thakrar V, Chan CC. EyePrintPRO therapeutic scleral contact lens: indications and outcomes. *Can J Ophthalmol*. 2018;53(1):66-70. doi:10.1016/j.jcjo.2017.07.026

98. Osaki TH, Sant'Anna AE, Osaki MH, et al. Management of Severe Cicatricial Entropion With Labial Mucous Membrane Graft in Cicatricial Ocular Surface Disorders. *J Craniofac Surg*. 2018;29(6):1531-1534. doi:10.1097/SCS.0000000000004584

99. Sato S, Kanbe T, Tamaki Z, et al. Clinical features of Stevens-Johnson syndrome and toxic epidermal necrolysis. *Pediatr Int*. 2018;60(8):697-702. doi:10.1111/ped.13613

100. Sevik MO, Turhan SA, Toker E. Topical Treatment of Persistent Epithelial Defects with a Matrix Regenerating Agent. *J Ocul Pharmacol Ther*. 2018;34(9):621-627. doi:10.1089/jop.2018.0025

101. Baş Z, Uçakhan Gündüz Ö. Sutureless Amniotic Membrane Transplantation in a Pediatric Patient with Acute Toxic Epidermal Necrolysis. *Turk J Ophthalmol*. 2019;49(6):356-360. doi:10.4274/tjo.galenos.2019.13333

102. Choe HR, Yoon CH, Kim MK. Ocular Surface Reconstruction Using Circumferentially-trephined Autologous Oral Mucosal Graft Transplantation in Limbal Stem Cell Deficiency. *Korean J Ophthalmol*. 2019;33(1):16-25. doi:10.3341/kjo.2018.0111

103. Gopakumar V, Agarwal S, Srinivasan B, Krishnakumar S, Krishnan UM, Iyer G. Clinical Outcome of Autologous Cultivated Oral Mucosal Epithelial Transplantation in Ocular Surface Reconstruction. *Cornea*. 2019;38(10):1273-1279. doi:10.1097/ICO.0000000000002082

104. Hashimoto H, Miyachi H, Kataoka K, Maru Y, Togawa Y, Matsue H. Case of fertility treatment-induced Stevens-Johnson syndrome with a severe ocular complication. *J Dermatol*. 2019;46(11):1042-1045. doi:10.1111/1346-8138.15072

105. Iyer G, Srinivasan B, Agarwal S, et al. Boston Type 2 keratoprosthesis- mid term outcomes from a tertiary eye care centre in India. *Ocul Surf*. 2019;17(1):50-54. doi:10.1016/j.jtos.2018.08.003

106. Kara A, Devrim İ, Çağlar İ, et al. Stevens-Johnson syndrome and toxic epidermal necrolysis: a report of six cases. *Turk J Pediatr*. 2019;61(4):538-543. doi:10.24953/turkjped.2019.04.010

107. Lee SM, Kim YJ, Choi SH, Oh JY, Kim MK. Long-term effect of corneoscleral contact lenses on refractory ocular surface diseases. *Cont Lens Anterior Eye*. 2019;42(4):399-405. doi:10.1016/j.clae.2018.10.011

108. Shanbhag SS, Chodosh J, Saeed HN. Sutureless amniotic membrane transplantation with cyanoacrylate glue for acute Stevens-Johnson syndrome/toxic epidermal necrolysis. *Ocul Surf*. 2019;17(3):560-564. doi:10.1016/j.jtos.2019.03.001

109. Shanbhag SS, Rashad R, Chodosh J, Saeed HN. Long-Term Effect of a Treatment Protocol for Acute Ocular Involvement in Stevens-Johnson Syndrome/Toxic Epidermal Necrolysis. *Am J Ophthalmol*. 2019;208:331-341. doi:10.1016/j.ajo.2019.07.006

110. Wang Y, Rao R, Jacobs DS, Saeed HN. Prosthetic Replacement of the Ocular Surface Ecosystem Treatment for Ocular Surface Disease in Pediatric Patients With Stevens-Johnson Syndrome. *Am J Ophthalmol*. 2019;201:1-8. doi:10.1016/j.ajo.2019.01.006

111. Xiang Q, Gao X, Fang J, et al. Lacrimal passage irrigation in children with Stevens-Johnson syndrome or toxic epidermal necrolysis: a five-year retrospective study. *BMC Ophthalmol*. 2019;19(1):22. doi:10.1186/s12886-018-1014-9

112. Yoon HJ, Kim MK, Seo KY, Ueta M, Yoon KC. Effectiveness of photodynamic therapy with verteporfin combined with intrastromal bevacizumab for corneal neovascularization in Stevens-Johnson syndrome. *Int Ophthalmol*. 2019;39(1):55-62. doi:10.1007/s10792-017-0786-x

113. Abrol A, Gulanikar A, Thakre S, Patel A. Study of Ocular Manifestations of Stevens-Johnson Syndrome/Toxic Epidermal Necrolysis. *Indian Dermatol Online J*. 2020;11(4):570-574. doi:10.4103/idoj.IDOJ_377_19

114. Alvarado-Villacorta R, García-Carmona KP, Martínez-Pardo ME, Vázquez-Maya L. Allogeneic Limbal Epithelial Transplantation Modified With Solid Platelet-Rich Plasma for

Bilateral Limbal Stem Cell Deficiency. *Cornea*. 2020;39(10):1311-1314. doi:10.1097/ICO.0000000000002321

115. de la Sen-Corcuera B, Montero-Iruzubieta J, Sánchez-Ávila RM, et al. Plasma Rich in Growth Factors for the Treatment of Cicatrizing Conjunctivitis. *Clin Ophthalmol*. 2020;14:1619-1627. doi:10.2147/OPTH.S252253

116. Itoi M, Ueta M, Ogino K, et al. Clinical trial to evaluate the therapeutic benefits of limbal-supported contact lens wear for ocular sequelae due to Stevens-Johnson syndrome/toxic epidermal necrolysis. *Cont Lens Anterior Eye*. 2020;43(6):535-542. doi:10.1016/j.clae.2020.05.007

117. Nakatsuka AS, Lin A. Ocular management of acute Stevens-Johnson syndrome in a 14-month-old child. *Can J Ophthalmol*. 2020;55(6):e213-e214. doi:10.1016/j.jcjo.2020.05.006

118. Shanbhag SS, Hall L, Chodosh J, Saeed HN. Long-term outcomes of amniotic membrane treatment in acute Stevens-Johnson syndrome/toxic epidermal necrolysis. *Ocul Surf*. 2020;18(3):517-522. doi:10.1016/j.jtos.2020.03.004

119. Shanbhag SS, Shah S, Singh M, Bahuguna C, Donthineni PR, Basu S. Lid-Related Keratopathy in Stevens-Johnson Syndrome: Natural Course and Impact of Therapeutic Interventions in Children and Adults. *Am J Ophthalmol*. 2020;219:357-365. doi:10.1016/j.ajo.2020.07.006

120. Shegaonkar SH. Bilateral panophthalmitis following toxic epidermal necrolysis: A case report. *Indian J Ophthalmol*. 2020;68(3):538-540. doi:10.4103/ijo.IJO_1208_19

121. Shimazaki J, Satake Y, Higa K, Yamaguchi T, Noma H, Tsubota K. Long-term outcomes of cultivated cell sheet transplantation for treating total limbal stem cell deficiency. *Ocul Surf*. 2020;18(4):663-671. doi:10.1016/j.jtos.2020.06.005

122. Sudana P, Basu S, Shanbhag SS. Oral mucous membrane grafts for total symblepharon and lid margin keratinisation post Stevens-Johnson syndrome. *BMJ Case Rep*. 2020;13(10)doi:10.1136/bcr-2020-239383

123. Chan S, Gole GA, Lee GA. Amniotic Membrane-Covered Conformer and Fibrin Glue for Toxic Epidermal Necrolysis. *Cornea*. 2021;40(4):525-528. doi:10.1097/ICO.0000000000002591

124. Elhusseiny AM, Gise R, Scelfo C, Mantagos IS. Amniotic membrane transplantation in a 2-month-old infant with toxic epidermal necrolysis. *Am J Ophthalmol Case Rep*. 2021;21:101017. doi:10.1016/j.ajoc.2021.101017

125. Hall LN, Shanbhag SS, Rashad R, Chodosh J, Saeed HN. The effects of systemic cyclosporine in acute Stevens-Johnson syndrome/toxic epidermal necrolysis on ocular disease. *Ocul Surf*. 2021;19:128-132. doi:10.1016/j.jtos.2020.05.003

126. Huhtanen A, Lindsay RG. Management of Stevens-Johnson syndrome using a mini-scleral contact lens. *Clin Exp Optom*. 2021;104(2):233-236. doi:10.1111/cxo.13118

127. Jabbour S, Din N, Logeswaran A, Taberno Sanchez S, Ahmad S. Clinical Characteristics of Patients With Chronic Stevens-Johnson Syndrome Treated at a Major Tertiary Eye Hospital Within the United Kingdom. *Front Med (Lausanne)*. 2021;8:644795. doi:10.3389/fmed.2021.644795

128. Jovanovic N, Russell WW, Heisel CJ, Hood CT, Kahana A. Direct Injection of 5-Fluorouracil Improves Outcomes in Cicatrizing Conjunctival Disorders Secondary to Systemic Disease. *Ophthalmic Plast Reconstr Surg*. 2021;37(2):145-153. doi:10.1097/IOP.0000000000001717

129. Ma DH, Tsai TY, Pan LY, et al. Clinical Aspects of Stevens-Johnson Syndrome/Toxic Epidermal Necrolysis With Severe Ocular Complications in Taiwan. *Front Med (Lausanne)*. 2021;8:661891. doi:10.3389/fmed.2021.661891

130. Ma KS, Saeed HN, Chodosh J, et al. Ocular manifestations of anti-neoplastic immune checkpoint inhibitor-associated Stevens-Johnson syndrome/toxic epidermal necrolysis in cancer patients. *Ocul Surf*. 2021;22:47-50. doi:10.1016/j.jtos.2021.06.010

131. Mahmood AH, Alharbi AS, Almanea BA, Alsaati AF. Sutureless Amniotic Membrane (ProKera®) and Intravenous Immunoglobulin in the Management of Ocular Complications of Stevens-Johnson Syndrome-Toxic Epidermal Necrolysis Overlap. *Cureus*. 2021;13(8):e16989. doi:10.7759/cureus.16989

132. Maqsood S, Elsawah K, Dhillon N, et al. Management of Persistent Corneal Epithelial Defects with Human Amniotic Membrane-derived Dry Matrix. *Clin Ophthalmol*. 2021;15:2231-2238. doi:10.2147/OPTH.S299141

133. Mieno H, Ueta M, Kinoshita F, Teramukai S, Kinoshita S, Sotozono C. Corticosteroid Pulse Therapy for Stevens-Johnson Syndrome and Toxic Epidermal Necrolysis Patients With Acute Ocular Involvement. *Am J Ophthalmol*. 2021;231:194-199. doi:10.1016/j.ajo.2021.06.015

134. Mimouni M, Trinh T, Sorkin N, et al. Sutureless dehydrated amniotic membrane for persistent epithelial defects. *Eur J Ophthalmol*. 2021:11206721211011354. doi:10.1177/11206721211011354

135. Moon J, Lee SM, Hyon JY, Kim MK, Oh JY, Choi HJ. Large diameter scleral lens benefits for Asians with intractable ocular surface diseases: a prospective, single-arm clinical trial. *Sci Rep*. 2021;11(1):2288. doi:10.1038/s41598-021-82010-z

136. Ngowyutagon P, Prabhasawat P, Chirapapaisan C, et al. Successful Ocular Surface Reconstruction in Complete Ankyloblepharon With the Simple Oral Mucosal Epithelial Transplantation Technique: A Case Report. *Cornea*. 2021;40(11):1482-1486. doi:10.1097/ICO.0000000000002638

137. Pushker N, Gorimanipalli B, Sharma N, Kashyap S, Bajaj MS. Mucous membrane grafting (fibrin glue vs. suture) for lid margin pathologies in Stevens-Johnson syndrome: randomized comparative study. *Eye (Lond)*. 2021;35(7):1985-1992. doi:10.1038/s41433-020-01203-4

138. Sant'Anna A, Sant'Anna É BPP, Osaki TH, Pereira Gomes J. A new option for treatment of severe cicatricial entropion in patients with Stevens-Johnson syndrome. *Ocul Surf*. 2021;22:80-82. doi:10.1016/j.jtos.2021.07.005

139. Santamaria JA, Cancio LC, Reed D, et al. Complete Fusion of Both Eyelids in Stevens-Johnson Syndrome: Case Report. *J Burn Care Res*. 2021;42(5):1023-1025. doi:10.1093/jbcr/irab024

140. Shahraki T, Hassanpour K, Arabi A, Ansari I, Sadoughi MM. Corona virus disease 2019-associated Stevens-Johnson syndrome: a case report. *BMC Ophthalmol*. 2021;21(1):274. doi:10.1186/s12886-021-02033-y

141. Utine CA, Birlik M, Özizmirliler D, Karakaş A, Akbulut B, Durak I. TNF-α Inhibitors for the Management of Intractable Corneal Melt: Report of Three Cases and Review of the Literature. *Eye Contact Lens*. 2021;47(6):372-377. doi:10.1097/ICL.0000000000000770

142. Venugopal R, Nagpal R, Mohanty S, et al. Outcomes of Cultivated Oral Mucosal Epithelial Transplantation in Eyes With Chronic Stevens-Johnson Syndrome Sequelae. *Am J Ophthalmol*. 2021;222:82-91. doi:10.1016/j.ajo.2020.08.022

143. Yang Y, Fung SSM, Chew H, Mireskandari K, Ali A. Amniotic membrane transplantation for Stevens-Johnson syndrome/toxic epidermal necrolysis: the Toronto experience. *Br J Ophthalmol*. 2021;105(9):1238-1243. doi:10.1136/bjophthalmol-2020-316056

144. Aziza Y, Harada K, Ueta M, Fukuoka H, Kinoshita S, Sotozono C. Challenges in the management of bilateral eyelid closure in Stevens-Johnson Syndrome. *Am J Ophthalmol Case Rep*. 2022;26:101473. doi:10.1016/j.ajoc.2022.101473

145. Booranapong W, Kosrirukvongs P, Duangsa-Ard S, Kasetsinsombat K, Sa-Ngiamsuntorn K, Wongkajornsilp A. Transplantation of autologous cultivated oral mucosal epithelial sheets for limbal stem cell deficiency at Siriraj Hospital: a case series. *J Med Case Rep*. 2022;16(1):298. doi:10.1186/s13256-022-03502-8

146. Iannetti L, Liberali M, Armentano M, et al. Osteo-odonto-keratoprosthesis According to Strampelli Original Technique: A Retrospective Study With Up to 30 Years of Follow-up. *Am J Ophthalmol*. 2022;242:56-68. doi:10.1016/j.ajo.2022.05.015

147. Kanazawa M, Tominaga K, Kanamori A, et al. A Case of Stevens-Johnson Syndrome Complicated with Multimatrix System Mesalamine in Ulcerative Colitis. *Medicina (Kaunas)*. 2022;58(2)doi:10.3390/medicina58020276

148. Katz EA, Sunshine S, Mun C, et al. Combinatorial therapy with immunosuppressive, immunomodulatory and tear substitute eyedrops ("Triple Play") in Recalcitrant Immunological Ocular Surface Diseases. *Ocul Surf*. 2022;23:1-11. doi:10.1016/j.jtos.2021.11.002

149. Komai S, Inatomi T, Nakamura T, et al. Long-term outcome of cultivated oral mucosal epithelial transplantation for fornix reconstruction in chronic cicatrising diseases. *Br J Ophthalmol*. 2022;106(10):1355-1362. doi:10.1136/bjophthalmol-2020-318547

150. Liao J, Asghari B, Carrasquillo KG. Regression of corneal opacity and neovascularization in Stevens-Johnson syndrome and Toxic Epidermal Necrolysis with the use of prosthetic replacement of the ocular surface ecosystem (PROSE) treatment. *Am J Ophthalmol Case Rep*. 2022;26:101520. doi:10.1016/j.ajoc.2022.101520

151. Mitani K, Hida S, Fujino H, Sumimoto S. Rare case of Stevens-Johnson syndrome with bronchiolitis obliterans as a chronic complication. *BMJ Case Rep*. 2022;15(4)doi:10.1136/bcr-2022-249224

152. Pradeep TG, Shetti SA. Ocular manifestations in acute stage Stevens-Johnson syndrome/toxic epidermal necrolysis - A retrospective study in a tertiary hospital in South India. *Taiwan J Ophthalmol*. 2022;12(2):184-190. doi:10.4103/tjo.tjo_13_21

153. Ryu S, Jun I, Kim TI, Seo KY, Kim EK. Pembrolizumab-induced Stevens-Johnson Syndrome with Severe Ocular Complications. *Ocul Immunol Inflamm*. 2022;30(6):1533-1535. doi:10.1080/09273948.2021.1896006

154. Sharma S, Singh S, Basu S, Shanbhag SS. Chronic Ocular Sequelae and Subsequent Surgical Interventions in Stevens-Johnson Syndrome After Amniotic Membrane Transplantation. *Cornea*. 2022;41(5):632-634. doi:10.1097/ICO.0000000000002816

155. Sims JR, Kozlova A, Ostrovsky A. Modified technique for sutureless amniotic membrane transplantation in acute Stevens-Johnson syndrome using fibrin sealant. *Ocul Surf*. 2022;25:89-91. doi:10.1016/j.jtos.2022.05.006

156. Susiyanti M, Kurnia DA, Fasha I, et al. Treatment of Severe Dry Eye in Stevens-Johnson Syndrome with Umbilical Cord Serum Eye Drops. *Clin Ophthalmol*. 2022;16:4089-4095. doi:10.2147/OPTH.S385078

157. Arboleda A, Phansalkar R, Amescua G, et al. Preparing the Ocular Surface for a Boston Keratoprosthesis Type 1 Through En Bloc Minor Salivary Gland Transplantation and Mucous Membrane Grafting in End-Stage Stevens-Johnson Syndrome. *Cornea*. 2023;42(7):912-916. doi:10.1097/ICO.0000000000003262

158. Asghari B, Carrasquillo KG, Kwok A, Sippel KC. Use of PROSE for long-term ocular surface support in patients with a permanent keratoprosthesis. *Am J Ophthalmol Case Rep*. 2023;32:101919. doi:10.1016/j.ajoc.2023.101919

159. Bourke CM, Cummings BK, Hurley DJ, Murphy CC, Chamney S. Isolated Ocular Stevens-Johnson Syndrome Caused by Lymecycline in a Patient with Underlying Ulcerative Colitis. *J Clin Med*. 2023;12(16)doi:10.3390/jcm12165259

160. Ceylan A, Mergen B, Aydin FO, Avci E, Yildirim Y. Sutureless Amniotic Membrane Transplantation Using Pediatric Nasogastric Tube for Patients With Acute Stevens-Johnson Syndrome/Toxic Epidermal Necrolysis. *Eye Contact Lens*. 2023;49(5):199-203. doi:10.1097/ICL.0000000000000986

161. Chiu HI, Tsai CC. Self-Retained, Sutureless Amniotic Membrane Transplantation for the Management of Ocular Surface Diseases. *J Clin Med*. 2023;12(19)doi:10.3390/jcm12196222

162. Doctor MB, Rajagopal RN, Basu S. Simple oral mucosal epithelial transplantation (SOMET) for ocular surface reconstruction in Stevens-Johnson Syndrome: A case report. *Int J Surg Case Rep*. 2023;110:108643. doi:10.1016/j.ijscr.2023.108643

163. Gupta N, Singh G, Bashir H, Sangwan V, Mathur U. Managing chronic inflammation in ocular sequelae of Stevens Johnson Syndrome to restore vision. *Ocul Surf*. 2023;28:40-41. doi:10.1016/j.jtos.2023.01.008

164. Hooshmandi S, Hassanpour K, Veisi A, et al. Management of Large Conjunctival Cysts in a Patient with Stevens-Johnson Syndrome: A Case Report and Review of the Literature. *Case Rep Ophthalmol*. 2023;14(1):528-534. doi:10.1159/000533648

165. Matsumoto K, Ueta M, Inatomi T, et al. Topical Betamethasone Treatment of Stevens-Johnson Syndrome and Toxic Epidermal Necrolysis with Ocular Involvement in the Acute Phase. *Am J Ophthalmol*. 2023;253:142-151. doi:10.1016/j.ajo.2023.05.010

166. Mimouni M, Cole E, Kim SJ, et al. Outcomes of keratolimbal allograft from ABO compatible donors for severe bilateral limbal stem cell deficiency. *Ocul Surf*. 2023;27:48-53. doi:10.1016/j.jtos.2022.11.002

167. Mortensen XM, Shenkute NT, Zhang AY, Banna H. Clinical Outcome of Amniotic Membrane Transplant in Ocular Stevens-Johnson Syndrome/Toxic Epidermal Necrolysis at a Major Burn Unit. *Am J Ophthalmol*. 2023;256:80-89. doi:10.1016/j.ajo.2023.07.026

168. Ravindra AP, Sinha R, Bari A, et al. Retinol palmitate in management of chronic Steven-Johnson Syndrome with ocular surface keratinization. *Ocul Surf*. 2023;30:160-167. doi:10.1016/j.jtos.2023.09.002

169. Shamloul G, Desai M, Laslett N. An Unusual Case of Stevens-Johnson/Toxic Epidermal Necrolysis Overlap Syndrome in HER2 (Human Epidermal Growth Factor Receptor 2)-Positive Breast Cancer Patient Treated With Docetaxel. *Cureus*. 2023;15(4):e37590. doi:10.7759/cureus.37590

170. Shlager G, Nakhla MN, Pritchett D, Brocks D. Case report: Concomitant use of nightly vitamin A ointment with daily PROSE wear for ocular surface disease associated with chronic Stevens-Johnson syndrome. *Am J Ophthalmol Case Rep*. 2023;32:101943. doi:10.1016/j.ajoc.2023.101943

171. Shree N, Das S, Arya D, Srivastava A, Singh A, Sangwan V. Single-Staged Surgical Correction of Eyelid Sequelae Along With Lid Margin Mucous Membrane Grafting in Stevens-Johnson Syndrome and Other Cicatricial Ocular Surface Diseases. *Cornea*. 2023;42(4):404-411. doi:10.1097/ICO.0000000000003021

172. Singh S, Basu S. 5-Fluorouracil as a targeted lacrimal gland therapy for chronic Stevens-Johnson syndrome: A pilot study. *Indian J Ophthalmol*. 2023;71(4):1626-1629. doi:10.4103/IJO.IJO_2647_22

173. Singh S, Basu S, Jakati S. Cicatricial Entropion in Chronic Cicatrizing Conjunctivitis: Potential Pathophysiologic Mechanisms and Long-Term Outcomes of a Modified Technique. *Ophthalmic Plast Reconstr Surg*. 2023;39(6):563-569. doi:10.1097/IOP.0000000000002400

174. Suzuki K, Watanabe Y, Imai Y, Yamaguchi Y. Eyelid and Vaginal Adhesions as Severe Sequelae of Toxic Epidermal Necrolysis. *Cureus*. 2023;15(7):e41496. doi:10.7759/cureus.41496

175. Tsai TY, Chang HT, Weng SW, et al. Ocular surface reconstruction of Steven Johnson syndrome / toxic epidermal necrolysis affected eye - A case report. *Heliyon*. 2023;9(1):e12590. doi:10.1016/j.heliyon.2022.e12590

176. Yoshikawa Y, Ueta M, Kinoshita S, Kida T, Sotozono C. Long-Term Benefits of Tear Exchangeable Limbal-Rigid Contact Lens Wear Therapy in Stevens-Johnson Syndrome Cases. *Eye Contact Lens*. 2023;49(6):247-253. doi:10.1097/ICL.0000000000000989

177. Zhang N, Geng X, Liu R, et al. Novel technique for amniotic membrane transplantation for acute Stevens-Johnson syndrome/toxic epidermal necrolysis patients. *Heliyon*. 2023;9(8):e18853. doi:10.1016/j.heliyon.2023.e18853

178. Zhu YF, Qiu WY, Xu YS, Yao YF. Clinical efficacy of a new surgical technique of oral mucosal epithelial transplantation for severe ocular surface disorders. *BMC Ophthalmol*. 2023;23(1):145. doi:10.1186/s12886-023-02879-4

179. Abulfateh FK, AlHaqbani YJ, Albuainain AS. Early Recognition and Management of Ocular Manifestations of Toxic Epidermal Necrolysis in a Pediatric Patient: A Case Report. *Cureus*. 2024;16(9):e70323. doi:10.7759/cureus.70323

180. Aziza Y, Imai K, Itoi M, et al. Strategic combination of cultivated oral mucosal epithelial transplantation and postoperative limbal-rigid contact lens-wear for end-stage ocular surface disease: a retrospective cohort study. *Br J Ophthalmol*. 2024;108(8):1177-1183. doi:10.1136/bjo-2023-323617

181. Bai H, Wang X, Wang Y, et al. Ornidazole induced Stevens-Johnson syndrome without body surface involved: A case report. *Medicine (Baltimore)*. 2024;103(5):e37164. doi:10.1097/MD.0000000000037164

182. Chen YK, Chi CL, Lai CH, Wu PL. Conjunctival squamous metaplasia on amniotic membrane in Stevens-Johnson syndrome: a case report. *BMC Ophthalmol*. 2024;24(1):484. doi:10.1186/s12886-024-03700-6

183. Foo VHX, Yueh LH, Mehta JS, Ong HS. Acute and chronic ocular outcomes in SJS/TEN patients treated with oral ciclosporin vs intravenous immunoglobulin. *Front Med (Lausanne)*. 2024;11:1398506. doi:10.3389/fmed.2024.1398506

184. Gueudry J, Terkmane MN, Tétart F, Muraine M, Ingen-Housz-Oro S. Promising response to Wharton's jelly eye drops in severe ocular involvement during acute phase of epidermal necrolysis and erythema multiforme major. *J Eur Acad Dermatol Venereol*. 2024;doi:10.1111/jdv.20113

185. Kwong EYL, Kuok MCI, Lam KF, Chan WKY. Case Report: Multi-targeted therapy in the treatment of severe toxic epidermal necrolysis. *Front Pediatr*. 2024;12:1460579. doi:10.3389/fped.2024.1460579

186. Pan LY, Wang CW, Tsai TY, et al. Post hoc Analysis of Role of Etanercept in Ocular Sequelae of Stevens-Johnson Syndrome/Toxic Epidermal Necrolysis. *Ophthalmology*. 2024;131(7):864-866. doi:10.1016/j.ophtha.2024.03.023

187. Peng R, Chi M, Xiao G, et al. The outcomes of corneal sight rehabilitating surgery in Stevens-Johnson syndrome: case series. *BMC Ophthalmol*. 2024;24(1):205. doi:10.1186/s12886-024-03461-2

188. Rashad R, Kwan JT, Shanbhag SS, et al. Long-term outcomes of glued (sutureless) amniotic membrane transplantation in acute Stevens-Johnson syndrome/toxic epidermal necrolysis: a comparative study. *Br J Ophthalmol*. 2024;108(11):1508-1513. doi:10.1136/bjo-2023-324076

189. Sharma N, Kumar V, Bari A, et al. The clinical outcomes of minor salivary gland transplantation for severe dry eye disease secondary to chronic Stevens-Johnson syndrome. *Ocul Surf*. 2024;34:277-282. doi:10.1016/j.jtos.2024.08.010

190. Sharma N, Venugopal R, Nagpal R, et al. Evaluation of adjuvant role of topical cyclosporine 1% in acute Stevens-Johnson syndrome: a randomised control trial. *Br J Ophthalmol*. 2024;doi:10.1136/bjo-2023-324901

191. Soma T, Oie Y, Takayanagi H, et al. Induced pluripotent stem-cell-derived corneal epithelium for transplant surgery: a single-arm, open-label, first-in-human interventional study in Japan. *Lancet*. 2024;404(10466):1929-1939. doi:10.1016/S0140-6736(24)01764-1

192. Subramanian M, Balaji J. Piggyback Scleral Contact Lens to Enhance Cosmesis and Comfort in Uniocular Stevens-Johnson Syndrome. *Eye Contact Lens*. 2024;doi:10.1097/ICL.0000000000001143

193. Wróblewska-Czajka E, Dobrowolski D, Wylęgała A, Jurkunas UV, Wylęgała E. Outcomes of Boston Keratoprosthesis Type I Implantation in Poland: A Retrospective Study on 118 Patients. *J Clin Med*. 2024;13(4)doi:10.3390/jcm13040975

194. Yao A, Singh S, Bagga B, Patel B, Malhotra R. Oral mucous membrane tarsal patch grafting: broadening indications and long-term outcomes. *Orbit*. 2024:1-6. doi:10.1080/01676830.2024.2429570
